# O_2_‐Accessible Fe–N_4_ Active Site Density Boosts Efficient Oxygen Reduction to Fuel‐Cell Level

**DOI:** 10.1002/adma.202521600

**Published:** 2026-02-16

**Authors:** Tianyu Zhang, Chen Liang, Shilun Sun, Shibo Xi, Zhongbin Zhuang, Jinliang Yuan, Zheng Xiao Guo, Junfeng Liu

**Affiliations:** ^1^ Faculty of Maritime and Transportation Ningbo University Ningbo China; ^2^ State Key Laboratory of Chemical Resource Engineering Beijing University of Chemical Technology Beijing China; ^3^ Institute of Sustainability for Chemicals Energy and Environment Agency for Science Technology and Research Singapore Singapore; ^4^ State Key Laboratory of Organic‐Inorganic Composites and Beijing Advanced Innovation Center for Soft Matter Science and Engineering Beijing University of Chemical Technology Beijing China; ^5^ Hong Kong Quantum AI Lab AIR@InnoHK of Hong Kong Government Hong Kong SAR China; ^6^ Department of Chemistry The University of Hong Kong Hong Kong SAR China

**Keywords:** fuel cell, O_2_ mass transport, oxygen reduction, single‐atom catalysts, structural design

## Abstract

Not all sites with intrinsic activity show efficacy in practical catalysis due to inaccessibility or diffusion limitation, necessitating rational design of well‐connected hierarchical nanostructures to guarantee accessibility. Herein, the case is thoroughly investigated by way of atomically dispersed Fe–NC catalysts for the dominant O_2_ gas‐consuming reduction (ORR). A pH‐dependent nanostructure manipulation strategy was developed to form solid, yolk‐shell, and hollow Fe–NC structures with similar overall density of quasi‐homogeneous Fe–N_4_ sites, providing a comparative platform to investigate O_2_ mass transport during ORR. Despite similar Fe loading, y‐Fe/NC structures achieve optimized O_2_‐accessible active site density (ASD) due to fine‐tuned porosity and connectivity for sufficient O_2_ accessibility. This observation is re‐affirmed by the observation of a relatively high *j_d_* for the y‐Fe/NC, which exceeds the theoretical value of a laminar flow pattern. This can be attributed to the increased O_2_‐accessible ASD, originated from the local recirculation effect induced by the unique structure. Consequently, the y‐Fe/NC exhibits half‐wave potential of 0.82 V and *j_d_* of 7.66 mA cm^−2^, outperforming counterparts and state‐of‐the‐art catalysts. Moreover, the optimized y‐Fe/NC remains effective in fuel cell with power density of 1.03 W cm^−2^, demonstrating the essential roles of rationally designed nanostructures.

## Graphical abstract

This study investigates the role of hierarchical nanostructure design in optimizing oxygen mass transport for the oxygen reduction. Using atomically dispersed Fe–N–C catalysts with solid, yolk‐shell, and hollow structures, it demonstrates that the yolk‐shell architecture provides superior O_2_ accessibility to active Fe–N_4_ sites. This results in an enhanced diffusion current and exceptional catalytic performance in both acidic electrolyte and a practical fuel cell, highlighting the critical link between nanostructure and reactivity.

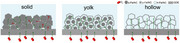

## 1 Introduction

Proton‐exchange membrane fuel cells (PEMFC), which efficiently convert hydrogen into electricity, represent an appealing solution for green energy supply [1, 2, 3]. However, the commercialization of precious‐group metal (PGM) based cathode to compensate for sluggish oxygen reduction reaction (ORR) is still limited by the earth scarcity of Pt, necessitating low‐cost alternatives [4]. Recently, single‐atom catalysts (SACs) become strong contenders due to their unique properties [5, 6, 7, 8], including Sn [9], Mn [10], Co [11], and Fe [12]. Particularly, single iron atoms coordinated with nitrogen dispersed on a carbon matrix (Fe–NC) yield encouraging ORR performance [13, 14, 15, 16]. However, Fe–NC still falls short of the adequate activity required to replace Pt, highlighting the necessity for further improvement [17]. Since limited success has been achieved so far in enhancing the intrinsic activity of Fe–NC [18], an alternative approach is to increase the catalytic site density (SD) of atomically dispersed Fe–N_x_ moieties [19, 20]. However, as the loading of single atoms increases, the corresponding utilization efficiency shows a significant decline, indicating that not all Fe–N_x_ sites are active in ORR; only those located at the triple‐phase boundary of electrode, electrolyte (proton) and O_2_ actively participate in the ORR as active sites [21, 22, 23, 24].

In PEMFC, while the proton supply through the membrane is sufficient, efficient oxygen mass transport (OMT) becomes the key process in determining site accessibility and availability [25, 26, 27]. Specifically, only a small fraction of Fe–N_x_ near the external surface (less than 5%) in Fe/NC nanoparticles are active [28, 29], while the majority are buried within the dense carbon matrix and remain inaccessible and inactive due to limited O_2_ diffusivity in the structure [30]. Therefore, an effective ORR catalyst requires well‐connected hierarchical porosities to address the challenge of inadequate O_2_ penetration into the micropores, thereby maximizing O_2_‐accessible active SD (ASD) [31, 32].

The rotating disc electrode (RDE) is a widely employed electrochemical technique for investigating the ORR kinetics and assessing catalyst activity. However, the translation of catalyst activity observed in RDE studies into MEA applications encounters a significant disparity. The crucial performance evaluation descriptors, including half‐wave potential (*E_1/2_*) and diffusion‐limited current density (*j_d_*) in RDE, cannot be directly correlated to the voltage loss related to the ohmic region and the power density in PEMFC [33]. In comparison to RDE experiments conducted in liquid electrolytes, the thicker catalyst layers employed at cathodes within MEAs significantly inhibit the transport of both O_2_ and protons. Notably, when transitioning from RDE‐level testing to PEMFC‐level evaluation, very few Fe–NC catalysts demonstrate comparable activity [34, 35, 36]. Such activity inconsistency could be attributed to the different reaction environments of the catalyst in RDE and PEMFC, particularly the variation in local O_2_ concentration around active sites caused by OMT [37]. Moreover, the catalyst layer needs to be much thicker compared with the PGM catalysts due to the lower density of Fe–N_x_ moieties, resulting in significant OMT resistance [38]. Given the demand for high current densities at low polarization voltages, a substantial influx of O_2_ around the active sites becomes crucial. Therefore, constructing O_2_‐accessible Fe–N_x_ active sites on hierarchical porous carbon supports via nanostructure optimization to enhance OMT within PEMFCs is an imperative need for fuel‐cell applications [39].

Moreover, the identification of O_2_‐accessible ASD for Fe/NC remains a huge challenge, requiring accurate measurements of both mass‐specific SD (MSD) [40] and active surface area. For example, X‐ray absorption spectroscopy (XAS) [41], aberration‐corrected high‐angle annular dark‐field scanning transmission electron microscopy (AC‐HAADF‐STEM) [42], and Mössbauer spectroscopy [43], are inherently bulk techniques and incapable of differentiating between electrochemically accessible and inaccessible Fe sites. Traditional probe chemisorption techniques to identify MSD using CO, NO, or CN^−^ often lack chemical specificity and exhibit poor reproducibility due to complex electrochemical conditions [44]. The electrochemical active surface area (ECSA) [45], determined through in situ electrochemical CO stripping or H/metal underpotential desorption, is considered a precise metric for assessing the active surface area of PGMs. However, these methods are ineffective for Fe/NC systems [46]. Furthermore, traditional approaches of Brunner‐Emmet‐Teller (BET) analysis and double layer capacitance (C_dl_)‐based ECSA assessment are unable to eliminate the influence of porous carbon support [47]. In short, accurately evaluating Fe/NC performance is challenging, requiring comprehensive and in‐depth analysis [48].

Herein, we developed an etching‐pyrolysis procedure to precisely tune the structure of hierarchical porous carbon nanoreactors, from solid (s‐) to yolk‐shell (y‐) to hollow (h‐) structures, thereby elucidating distinct OMT mechanisms. Tannic acid (TA), as a weak organic acid and cheap natural polyphenol, was selected as a pH regulator, etchant, and surface modifier to achieve nanostructure regulation, while maintaining the homogeneity of the SAC microenvironment. Meanwhile, the post‐loading strategy of Fe ensures the consistency of the total loading. Starting from the inconsistent RDE‐based ORR activity, we investigate the significant roles of O_2_ diffusion by regulating the porosity and connectivity of nanoreactors, thereby achieving the maximum O_2_‐accessible Fe–N_x_ active sites in y‐Fe/NC. The O_2_‐accessible Fe–N_x_ ASD was comprehensively analyzed using a combination of multiple techniques, including BET analysis, C_dl_ measurement, CO cryoprobe technique, and NO probe technique, alongside the mathematical model‐based parameter fitting. It is worth noting that *j_d_* in RDE exhibits a positive correlation with O_2_‐accessible Fe–N_x_ ASD when considering the structure‐induced local recirculation effect, a factor that is commonly overlooked in RDE studies, even under standard procedural controls.

Moreover, the OMT resistance in regulated Fe–N_x_ nanostructures was thoroughly examined through diffusion kinetics experiments, in situ bubble pump consumption chronoamperometry (BPCC) strategy, and finite element method (FEM) simulations. All experimental results and simulations consistently reveal that the Fe–N_4_ sites anchored on y‐Fe/NC exhibit maximum O_2_‐accessible Fe–N_4_ ASD as well as a reduced O_2_ mass transfer resistance. Consequently, this catalyst demonstrates promising performance for acidic ORR with the highest *E_1/2_* of 0.82 V and the largest *j_d_* of 7.66 mA cm^−2^, outperforming those of s‐Fe/NC (*E_1/2_*, 0.78 V and *j_d_*, 5.16 mA cm^−2^), h‐Fe/NC (*E_1/2_*, 0.80 V and *j_d_*, 5.67 mA cm^−2^). Furthermore, y‐Fe/NC exhibited a competitive peak power density (*P_max_*) of 1.03 W cm^−2^ under H_2_‐O_2_ and 0.34 W cm^−2^ under H_2_‐Air, demonstrating the important roles of rationally designed hierarchical nanostructures in enhancing OMT and maximizing O_2_‐accessible ASD within PEMFC system.

## 2 Results and Discussion

### 2.1 Structural Differentiation of Fe/NC Nanoreactors

The Fe/NC nanoreactors were synthesized using a post‐loading strategy, and the manipulation of their structure was achieved through a pH‐dependent etching‐pyrolysis approach, as elaborated in Figure S1. First, ZIF‐8 (Figure S2) underwent etching and modification using TA to produce ZIF‐8@TA. By precisely controlling the etching rate of TA through pH modulation, we successfully achieved well‐regulated nanoreactor structures, ranging from solid to yolk‐shell to hollow architectures, depending on the specific pH values employed (Figures S3 and S4 and Note S1). Subsequent pyrolysis resulted in the formation of nitrogen‐doped carbon (NC) (Figure S5). The coordination of Fe was then achieved by ultrasonic treatment of NC samples in a FeCl_3_ solution for anchoring of Fe^3+^ ions [49]. Finally, the Fe/NC was obtained by subjecting the mixture to high‐temperature activation in the presence of urea.

**FIGURE 1:**
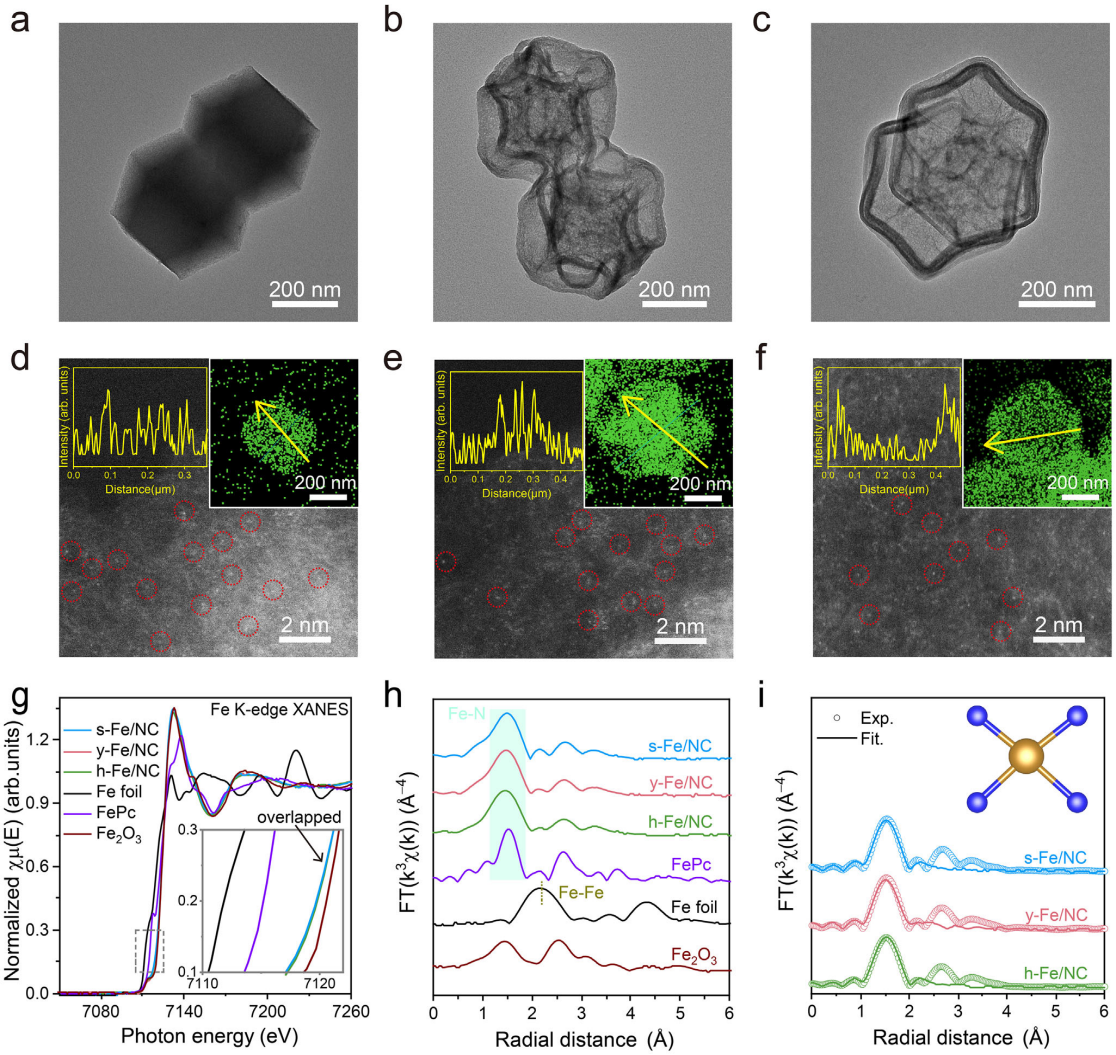
Structural characterizations of the s‐Fe/NC, y‐Fe/NC, and h‐Fe/NC. (a–c) TEM, (d–f) HAADF‐STEM and corresponding Fe elemental mapping (inset d–f) images of s‐Fe/NC (a,d), y‐Fe/NC (b,e), and h‐Fe/NC (c,f), respectively. (g–i) Fe K‐edge XANES spectra (g) (inset, magnified spectra), FT‐EXAFS spectra in the R space (h), and first‐shell fitting of the FT‐EXAFS (i) of the three SACs (inset, schematic model of Fe/NC: Fe (dark yellow) and N (blue)).

Transmission electron microscope (TEM) and scanning electron microscopy (SEM) images show the successful manipulation of the structures of Fe/NC nanoreactors, from solid (Figure 1a) to yolk‐shell (Figure 1b), and finally to hollow structures (Figure 1c), all exhibiting similar sizes (diameter of ∼400 nm) and uniform morphology as confirmed by Figures S6 and S7. No sub‐nanometer clusters or nanoparticles were detected via AC‐HAADF‐STEM (Figure 1d–f), except for discrete bright dots, providing evidence for the homogeneous dispersion of Fe atoms across the carbon matrix in all Fe/NC samples. The typical yolk‐shell structural features were clearly evidenced by the corresponding energy‐dispersive X‐ray spectroscopy (EDX) elemental mappings of Fe, N, and C on y‐Fe/NC, in contrast to its solid and hollow counterparts (inset Figure 1d–f; Figure S8). Through our post‐loading strategy, the overall level of Fe loading can be readily controlled, leading to similar levels of Fe loadings in s‐Fe/NC, y‐Fe/NC, and h‐Fe/NC, as determined by inductively coupled plasma atomic emission spectrometer analysis (Table S1).

### 2.2 Homogeneity of Fe–N_4_ Sites

Synchrotron‐based XAS was utilized to ascertain the coordinated environment of Fe single atoms in Fe/NC. A comparison of positions of Fe K‐edges for all Fe/NC with those of Fe foil, iron phthalocyanine (FePc), and Fe_2_O_3_ standards indicates that the valence states of Fe in Fe/NC is predominantly +3 (Figure 1g; Figure S9). It is worth noting that the spectra of s‐, y‐, and h‐Fe/NC are essentially overlapped, demonstrating the homogeneity of Fe–N sites. The absence of pre‐edge peak assigned to *1s*→*4p_z_* shakedown transition in Fe/NC reveals a broken *D_4h_* symmetry [50]. These non‐planar Fe sites with high spin possess superior ORR activity by facilitating oxygen adsorption [51]. Fourier‐transformed (FT) *k^3^*‐weighted extended X‐ray absorption fine structure (EXAFS) spectra of all Fe/NC samples (Figure 1h) verified that the three SAC samples are essentially free of Fe aggregates, as indicated by the absence of Fe–Fe scattering path, which was further confirmed by the wavelet transform analysis (Figure S10). Besides, the obvious peaks at ∼1.4 Å associated to Fe–N path were found in all Fe/NC samples, which is similar with the Fe–N_4_ contained in FePc [52]. To elucidate the local bonding environment of the Fe single sites, quantitative least‐square EXAFS curve‐fitting analysis was performed using fiducial distances in the standard crystal structure as scattering path (Figures S11–S13). The best‐fitting results of all Fe/NC confirmed that a Fe site coordinates with four N atoms (Figure 1i; Figure S14 and Table S2).

Subsequently, X‐ray photoelectron spectroscopy (XPS) was performed to investigate the chemical composition of Fe/NC nanoreactors. Negligible difference was found in the composition and proportion of N among Fe/NC samples (Figure S15a and Table S3), indicating the homogeneity of the Fe–N_x_ sites. The N coordinated structures were further analyzed by the soft XAS, which is sensitive to the local electronic configuration of the coordinated low‐z atoms. The N K‐edge spectra of all Fe/NC were dominated by two typical features: *1s*→π* transition and *1s*→σ* transition of the N─C bond [53]. After dividing the features in *1s*→π* region into aromatic C–N–C portion on the sites of pyridinic (∼399.5 eV) and pyrrolic (∼400.5 eV), and the N–3C bridging on the graphitic sites (∼402.5 eV), we confirm that all Fe/NC samples possess a highly consistent N species [54] (Figure S15b–d). Besides, the XPS Fe *2p_3/2_* peaks in all Fe/NC were located at 711.1 eV, indicating the valence states of all Fe atoms are +3 (Figure S15e) [55]. In addition, the peak at 700 cm^−1^ in Fourier‐transform infrared spectroscopy (FT‐IR) for y‐Fe/NC was assigned to Fe─N stretching, confirming the incorporation of Fe–N_x_ sites [56] (Figure S16a). Raman spectra of all three samples show similar ratios of D and G bands (I_D_/I_G_), indicating similar graphitization degree and defect levels of all Fe/NC samples (Figure S16b).

Moreover, density functional theory (DFT) simulations demonstrated that differences in the Fe microenvironment significantly affect the electronic structure, which can be detected by XAS, further demonstrating the homogeneity of our strategy (Figure S17) [57, 58]. Overall, our facile etching‐pyrolysis procedure allows us to precisely manipulate the nanostructure of Fe/NC‐based nanoreactors while maintaining the homogeneity of the Fe‐N_4_ sites.

### 2.3 ORR Activity of Fe/NC on RDE

**FIGURE 2:**
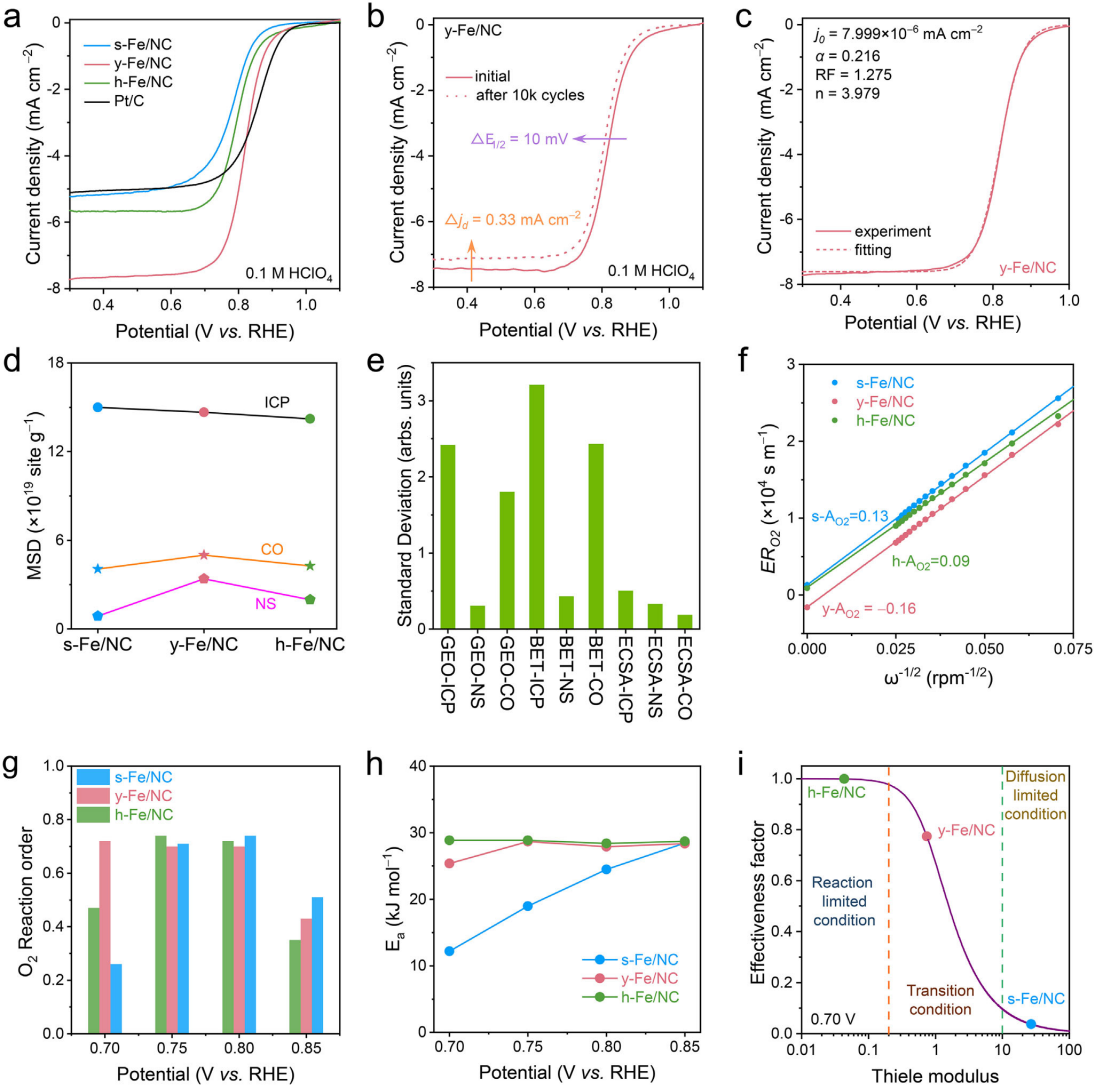
Acidic ORR activity of Fe/NC on RDE. (a) ORR polarization curves of all Fe/NC and Pt/C. (b) ORR polarization curves of y‐Fe/NC recorded before and after 10000 ADT cycles. (c) Experimental and fitting ORR polarization curves for y‐Fe/NC. (d) Measured MSD and (e) comparison on TOF at 0.8 V vs. RHE normalized by surface area and MSD of all Fe/NCs utilizing different strategies. Comparison of (f) experimental oxygen transfer resistance (ER_O2_) and structure correction value (A_O2_) and (g) O_2_ reaction order among all Fe/NCs. (h) The apparent activation energy of all Fe/NCs under 0.85 to 0.7 V versus. RHE. (i) The Thiele modulus at 0.7 V versus. RHE among all Fe/NCs.

The ORR performance‐structure relationship of Fe/NC‐based nanoreactors was investigated on RDE in O_2_‐saturated HClO_4_ (0.1 M) with commercial 20% Pt/C as a reference catalyst. After carefully screening a considerable number of catalyst loadings to fully unleash their catalytic potential (Figure S18 and Note S2), y‐Fe/NC exhibited promising 4e^−^ ORR activity (*E_1/2_* 0.82 V vs. RHE, Figure 2a; Figure S19), outperforming counterparts and most of state‐of‐the‐art catalysts (Table S4). Moreover, y‐Fe/NC demonstrated good stability, showing only a 10 mV decay after 10k cycles with no significant changes in morphology or valence state (Figure 2b; Figure S20). Notably, an interesting phenomenon was observed after employing standardized RDE testing protocols to eliminate the influence of experimental operations on *j_d_* [59, 60, 61]: the experimental *j_d_* of y‐Fe/NC (7.66 mA cm^−2^) significantly surpasses those of s‐Fe/NC (5.16 mA cm^−2^) and h‐Fe/NC (5.67 mA cm^−2^), even exceeding the corresponding theoretical value for a flat electrode (*j_td_*, 6.05 mA cm^−2^, Note S3).

To understand this discrepancy between *j_d_* and *j_td_* values in y‐Fe/NC, the ORR polarization mathematical model was first established (Note S4), and the experimental ORR polarization curves were fitted via Python‐based least squares method [62] (Figure S21). Here, we involved and expanded the concept of roughness factor (RF), which is defined as the ratio between the real tri‐phase interface area (A_real_) and the flat geometric area (A_geo_) of an electrode [63]. RF≤1 is very common in ORR due to insufficient catalyst loading and partial site blockage [64], making the assumption that reactants only move toward and across the electrode in laminar flow acceptable [65].

However, Figure 2c shows that the experimental RF value for y‐Fe/NC nanoreactors is as high as 1.27, which is significantly higher than that of Pt/C (0.86), s‐Fe/NC (0.85), and h‐Fe/NC (0.95) (Figure S22). This observation suggests that the laminar flow pattern may not applicable to y‐Fe/NC with enlarged area. Consequently, y‐Fe/NC exhibits better consistency with the porous RDE model (Note S5 and Figure S23) [66], which shows typical nonlinear dependence between current and rotating rate [67], indicating a local recirculation induced axial mass transport due to the unique yolk‐shell structure is the primary reason for the excessively high *j_d_* observed in y‐Fe/NC.

Considering the homogeneity of Fe–N_4_ active sites with identical intrinsic activity across all Fe/NC nanoreactors, the performance inconsistency is primarily attributed to the enlarged solid‐liquid‐gas tri‐phase interface resulting from the porosity of the nanostructures. The BET measurements first reveal that s‐Fe/NC exhibits the largest surface area, which is mainly attributed to the presence of numerous electrochemically inert micropores (Figure S24 and Table S5). However, C_dl_‐based ECSA assessments indicate that y‐Fe/NC possesses the highest ECSA value of 1190 cm_ESCA_^2^ (Figure S25 and Table S6).

Furthermore, MSD was quantitatively evaluated by ICP‐AES, gas diffusion‐dominated CO pulse chemisorption (Figure S26 and Table S7), and anion diffusion‐dominated nitrite sorption/desorption (NS) strategy (Figure S27). The results were summarized in Table S8 and represented in Figure 2d. Clearly, the MSD values from CO or NS strategy are much lower than those from ICP, indicate not all Fe–N_4_ sites are accessible and effective due to the diffusion limitation. Furthermore, the determined MSD_CO_ of all three samples was systematically larger than MSD_NS_, which can be attributed to the presence of Fe sites located within the gas‐accessible but electrolyte‐hindered pores [48]. It is worth noting that despite similar metal loading (as determined by MSD_ICP_), both the CO and NS strategies demonstrated that y‐Fe/NC exhibited higher MSD compared to h‐Fe/NC and s‐Fe/NC, indicating distinct mass transport behaviors arising from structure differences, leading more Fe sites are identifiable. In addition, the MSD differences observed through the NS strategy are more pronounced, indicating exceptionally rapid electrolyte transport behavior in the y‐Fe/NC. This observation aligns well with the previously discussed finding that y‐Fe/NC shows high perfusion rates in the porous RDE model [48].

Moreover, turnover frequency (TOF) values for all Fe/NCs were comprehensively studied (Figure S28 and Note S6). To avoid confusion, ASD and MSD are distinguished: ASD refers to Fe sites that can actually participate in ORR, namely sites that are accessible to O_2_ and can react, while MSD is the site density per catalyst mass quantified by a given method. ICP‐AES measures total bulk Fe and therefore includes inactive or inaccessible Fe, making it not a selective descriptor. CO pulse chemisorption probes gas‐accessible Fe sites and thus approximates O_2_‐accessible ASD, whereas nitrite stripping probes electrolyte‐accessible sites and may undercount sites in poorly wetted pores [48]. Since Fe sites in all nanoreactors show similar intrinsic activity, the optimal MSD method should minimize TOF dispersion after normalization. The TOF values at 0.8 V versus. RHE normalized by ECSA and MSD_CO_ exhibits the smallest standard deviation (Figure 2e), indicating that gas transport behavior is the primary factor distinguishing the apparent activity.

### 2.4 Enhanced OMT Effect

Based on the abundance of O_2_ surrounding the active sites, Fe–N_4_ can be categorized into two types: O_2_‐accsessible and O_2_‐inaccessible. The insufficient oxygen around certain Fe–N_4_ sites may be attributed to the inward migration of Fe atoms within porous NC particles during secondary pyrolysis, resulting in partially restricted oxygen access. Thus, we propose an additional indicator to describe O_2_ accessibility: the OMT resistance to the active sites, which results in significant variations in ORR performance due to the nanoreactor structures.

To verify the OMT effect, the experimental oxygen transfer resistance (*ER*_O2_) and structure correction value (*A*_O2_) of Fe/NC nanoreactors were quantified by regulating the rotating speed of RDE to evaluate the OMT resistance [68] (Figures S29 and S30 and Note S7). *A*_O2_ is a value only related to the structure of the Fe/NC nanoreactor with three types of OMT effect: limiting (*A*_O2_>0), facilitating (*A*_O2_<0), and zero (*A*_O2_ = 0). As shown in Figure 2f, y‐Fe/NC demonstrates a notable OMT facilitating effect with a negative *A*_O2_, suggesting permeability of electrolyte and oxygen flow within mesoporous and yolk‐shell structure to activate extra active surface in pores. However, both s‐Fe/NC and h‐Fe/NC exhibit OMT limiting effect. Note, the hollow cavity in h‐Fe/NC is easily flooding, renders it ineffective in providing enough active sites and tri‐phase boundary. Kinetic analysis was further performed to experimentally distinguish the role of OMT within the mix‐controlled region. The partial reaction order of O_2_ (*m_O2_*) was determined by performing ORR under different O_2_ partial pressures (Figure S31 and Note S8) [69]. *m_O2_* quantifies the extent to which the ORR rate is dependent on O_2_ concentration, with a higher *m_O2_* demonstrating a less diffusion limited in ORR [70]. In principle, *m_O2_* initially rise and then fall as potential decreases, reflecting the transition through kinetic‐, mixed‐, and diffusion‐controlled pathway of ORR. At the end of mixed‐controlled region, y‐*m_O2_* (0.72) is much larger than h‐*m_O2_* (0.47) and s‐*m_O2_* (0.26), suggesting the higher local O_2_ concentration of y‐Fe/NC with larger O_2_‐accessible ASD and enhanced OMT (Figure 2g).

Moreover, the apparent activation energies (E_a_) for ORR under mix‐controlled region were calculated using Arrhenius plots (Figure S32) to investigate the apparent reaction kinetics. As shown in Figure 2h, at the beginning of mix‐controlled region (0.85 V vs. RHE), all Fe/NCs were under kinetic control with similar apparent E_a_. As potential decreases, the apparent E_a_ of s‐Fe/NC significantly decreases while that of y‐Fe/NC and h‐Fe/NC remains essentially unchanged. Notably, the external O_2_ diffusion limitation can be excluded by using RDE with rotating speed of 1600 rpm [71]. Thus, the differences in apparent E_a_ demonstrate boosted OMT and smaller internal O_2_ diffusion blockage in y‐Fe/NC and h‐Fe/NC [72]. Moreover, the Thiele modulus *Ф* and effectiveness factor *η*, as indicators of internal diffusion, were then calculated to quantitively evaluate the internal OMT of Fe/NC nanoreactors at different potential (Figure 2i; Figure S33). At 0.7 V versus. RHE, s‐Fe/NC, y‐Fe/NC, and h‐Fe/NC were in diffusion limited condition, transition condition and reaction limited condition, respectively, demonstrating the enhanced OMT order: h‐Fe/NC>y‐Fe/NC>s‐Fe/NC.

Specifically, *η* of s‐Fe/NC was only 0.04, indicating the poor OMT prevented the O_2_ from moving to the interior of the active sites, caused the dead zone that cannot be utilized in the catalytic process. For h‐Fe/NC, the *η* was nearly 0.99, which effectively eliminating the internal OMT influence for all active sites. However, the lower active site number of h‐Fe/NC leads an unsatisfied performance (Table S8). The y‐Fe/NC with accessible *η* at 0.8 under transition conditions provides the optimized strategy with maximum tri‐phase boundaries and O_2_‐accessible site number for the ORR process. Moreover, the chemisorption behavior of O_2_ is further illustrated by O_2_ temperature program desorption (TPD). As shown in Figure S34, the higher signal area of y‐Fe/NC demonstrates denser and more exposed active sites. Besides, the peak position order (h‐Fe/NC<y‐Fe/NC<s‐Fe/NC) indicates that h‐Fe/NC exhibits lower diffusion resistance [30]. Overall, RDE‐based experiments confirm that both OMT and ASD are both important for efficient ORR, indicating the essential role of nanostructure regulation for further enhancing performance with structure‐induced local recirculation effect.

### 2.5 OMT Pathway via FEM Simulations and In Situ BPCC Strategy

**FIGURE 3:**
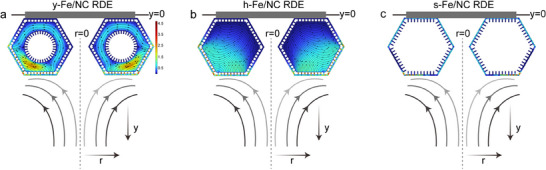
Illustration and modeling by CFD simulation. velocity field and streamlines of O_2_ around (a) y‐Fe/NC, (b) h‐Fe/NC, and (c) s‐Fe/NC.

High‐resolution computational fluid dynamics (CFD) simulations further elucidate the structure‐induced local recirculation. Figure 3 compares the velocity field and streamlines around all Fe/NC under identical RDE hydrodynamics (𝑦 = 0 at the electrode surface and *r* = 0 at the symmetry axis) [73, 74]. The defining distinction is whether the external shear can be transformed into internal convective renewal. As shown in Figure 3a, the inner core and outer shell create a confined core shell gap that induces a clear local recirculation cell near the lower rim, evidenced by curved/partly closed streamlines and locally intensified velocity. This recirculation continuously exchanges fluid between the bulk and the internal surfaces, enabling convective perfusion into the porous microenvironment. While for h‐Fe/NC (Figure 3b), partial internal flow exists but is weaker and more diffuse because the absence of a core reduces shear amplification within a narrow gap, leading to less effective internal renewal. For s‐Fe/NC, streamlines remain external with no internal circulation, indicating transport dominated by the classical boundary‐layer supply. Overall, the simulations support a structure‐driven transport hierarchy yolk shell > hollow > solid, where the yolk shell architecture uniquely promotes local recirculation, increasing oxygen renewal and the effective O_2_‐accessible active‐site utilization under RDE conditions.

**FIGURE 4:**
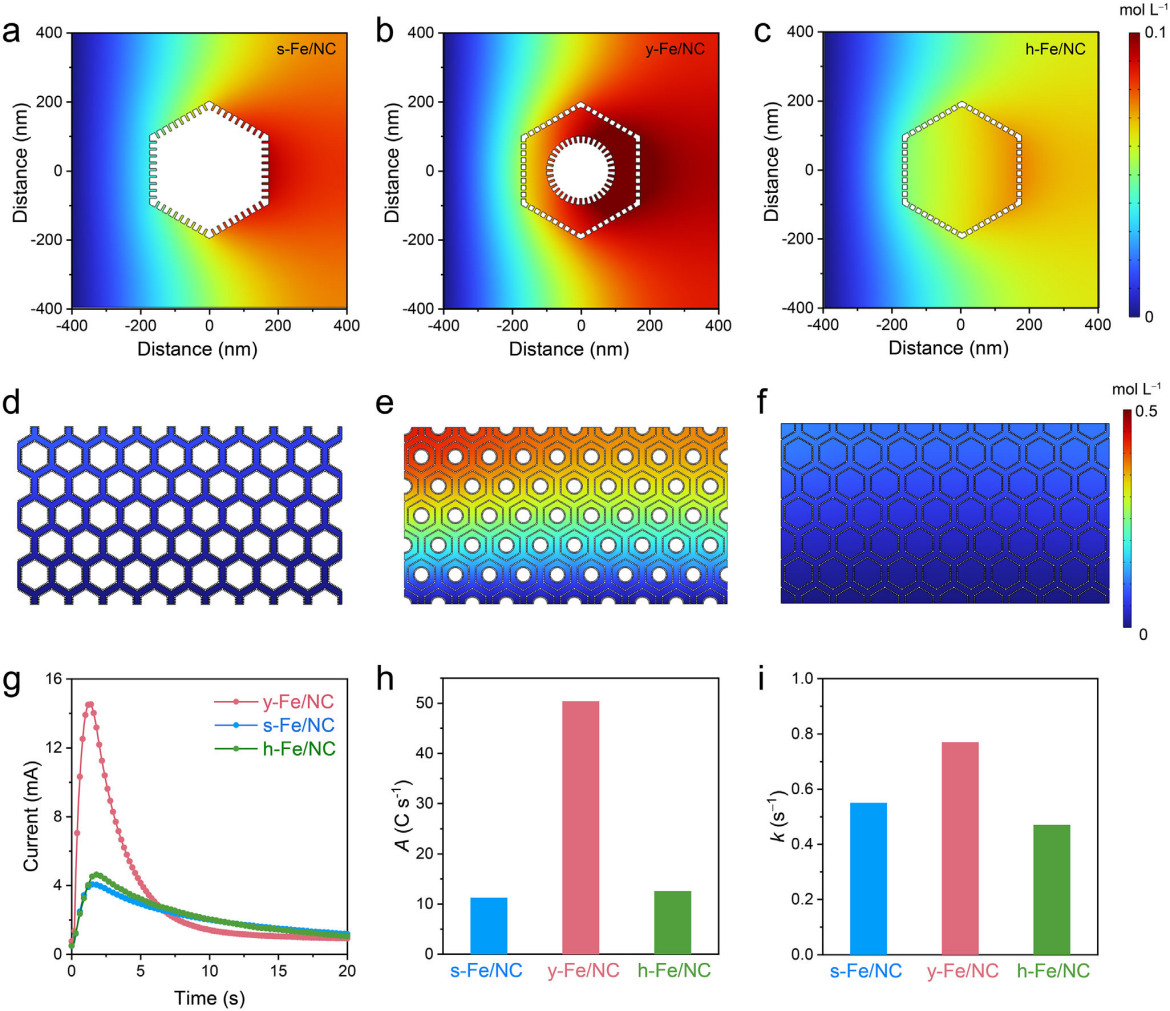
Illustration and modeling by FEM simulation. Distribution of the obtained H_2_O local concentration around the (a,d) s‐Fe/NC, (b,e) y‐Fe/NC, and (c,f) h‐Fe/NC under (a–c) single model or (d–f) array model. (g) ORR polarization curves for individual oxygen bubbles injected onto Fe/NC‐based GDE. Corresponding calculated (h) pre‐exponential factor (A) and (i) effective reaction rate constant (k) values derived from diffusion‐consumption expressions (i(t) = Akt·exp(–kt)) as a function of applied voltage.

Essentially, the above‐discussed Fe/NC film with high catalyst loading on RDE does not perfectly conform to the convective flow‐based RDE theory and is accompanied by non‐negligible vertical OMT. However, the OMT pathway through the cathode catalyst layer is crucial for the performance of PEMFC, especially in fully utilizing the inner catalyst layer. To elucidate the OMT behavior in gas‐diffusion electrodes (GDE) fabricated with designed Fe/NC nanoreactors, FEM simulations were conducted. The corresponding s‐, y‐, and h‐Fe/NC models were represented by different hexagons based on TEM imaging to simulate the distribution of local product, proton, and O_2_ concentration around a single model. As depicted in Figures S35–S37, both proton and O_2_ could diffuse to the catalyst surface and accumulate in voids, demonstrating that a more open structure enhances the OMT effect and facilitates kinetic access to active sites. However, y‐Fe/NC exhibited a higher product concentration compared to s‐Fe/NC and h‐Fe/NC, confirming that both SD and OMT are crucial in ORR (Figure 4a–c).

Impressively, significant product accumulation was observed in the voids of y‐Fe/NC, indicating the effectiveness on porosity regulation via nanostructural design on RDE‐level (Figure S38). Moreover, after scaling up the single‐particle model to a 5 × 9 hexagonal array model, we found that the three nanostructures consistently exhibited the same trends, demonstrating the effective regulation via nanostructural design at the GDE level. Although the h‐Fe/NC exhibited enhanced OMT behavior, the y‐Fe/NC sample demonstrated the highest product formation, which effectively balances both SD and OMT (Figure 4d–f; Figures S39–S41).

Subsequently, the oxygen bubble diffusion behavior and consumption dynamics were experimentally evaluated on Fe/NC‐based GDE, especially focusing on ASD and OMT coefficient. Ex situ droplet/gas‐bubble contact angle measurements showed that all Fe‐NCs are aerophilic, while y‐Fe/NC with yolk‐shell and hierarchical porous structures exhibits better gas affinity and accommodation due to the macropores in the void [75] (Figures S42–S44 and Note S9), suggesting that y‐Fe/NC is beneficial for the absorption of oxygen and further promote ORR process. Impressively, the gas capacity of y‐Fe/NC is higher than that of h‐Fe/NC, demonstrating the better resistance of the void against flooding. A long and stable current ruled out the possibility of bubbles stored in void being the cause of abnormally large current, demonstrating the continuously effective local recirculation effect (Figure S45). Moreover, in situ BPCC strategy [76] was introduced to precisely measure the ASD and OTM coefficient by observing evolution in current response to ORR of an injected O_2_ bubble on Fe/NC (Figures S46 and S47 and Note S10). The responsive ORR polarization of y‐Fe/NC exhibited the highest peak response current (Figure 4g), indicating the effectiveness of optimizing the balance between SD and OMT. Moreover, the pre‐exponential factor (*A*) and the effective reaction rate constant (*k*) derived from the extension‐consumption model represent OMT and the ASD, respectively (See details in Figure S48 and Note S10). The *A* value of y‐Fe/NC is 50 C s^−1^, much higher than that of s‐Fe/NC (11 C s^−1^) and h‐Fe/NC (13 C s^−1^), suggesting the enhanced OMT in y‐Fe/NC (Figure 4h). h‐Fe/NC failed to achieve OMT advantage in experiments as expected in simulation due to its excessive diffusion capacity leads flooding issue in BPCC, demonstrating strong resistance to flooding of yolk‐shell structure. Additionally, y‐Fe/NC exhibited a higher *k* value, suggesting a higher O_2_‐accessible ASD, consistent well with above analysis (Figure 4i).

### 2.6 ORR Performance of Fe/NC Nanoreactors in PEMFC

**FIGURE 5:**
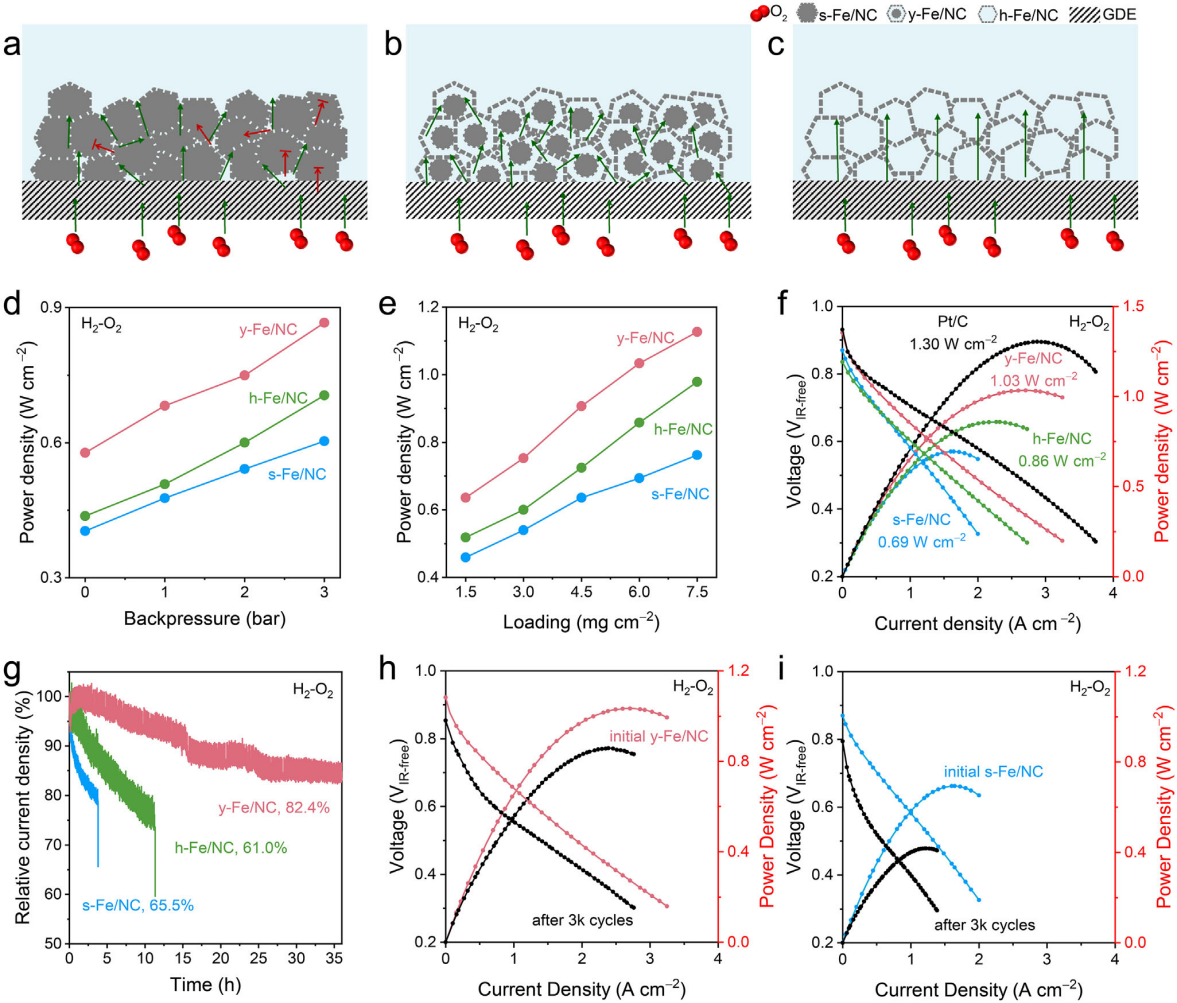
PEMFC performance. Schematic illustration of oxygen transport in (a) s‐Fe/NC, (b) y‐Fe/NC, and (c) h‐Fe/NC, where green arrows represent oxygen transport pathways and red arrows represent forbidden blocks. Relationship between powder density and (d) backpressure or (e) loading amount for all the Fe/NC. (f) Polarization and power density curves of Pt/C and Fe/NC nanoreactors under 2 bar H_2_‐O_2_. (g) Stability test of all Fe/NC nanoreactors at a constant cell potential of 0.65 V. Polarization and power density curves of (h) y‐Fe/NC and (i) s‐Fe/NC before and after 3000 square wave accelerated stress test. Test conditions: catalysts loading 6 mg cm^−2^ for Fe/NC nanoreactors and 0.1 mgPt cm^−2^ for Pt/C.

Inspired by the outstanding ORR performance benefit from the enlarged O_2_‐accessible ASD and enhanced OMT after nanoreactor structure design, the PEMFC performance of all Fe/NC was validated in the H_2_‐O_2_ and H_2_‐air fuel cells. Based on the above analysis, the OMT mechanism on s‐, y‐, and h‐Fe/NC nanoreactors‐based GDE was illustrated in Figure 5a–c, respectively. The enhanced OMT effect was first verified by measuring the performance of the as‐prepared electrode with low loading (3 mg cm^−2^) in H_2_‐O_2_ fuel cell under varying H_2_/O_2_ backpressures (i.e., 0, 1, 2, and 3 bar, Figure S49). In practical devices, backpressure is an effective method to regulate OMT. As backpressure increases, a significant rise in open‐circuit voltage (OCV) is observed in both s‐ and y‐Fe/NC, followed by a plateau, while the OCV of h‐Fe/NC remains essentially unchanged. This suggests that O_2_ distribution is insufficient in s‐Fe/NC and y‐Fe/NC, leading to the underutilized active sites (Figure S50). Simultaneously, the *P_max_* increases with the rise in backpressure, with a notably faster growth rate observed in y‐ and h‐Fe/NC, indicating the OMT is accelerated after nanostructure design (Figure 5d). The OMT process was further analyzed by equivalent resistance analysis based on electrochemical impedance spectroscopy (EIS), including charge transfer (Rct) and mass transport resistance (Rmt). The semicircles of y‐Fe/NC‐based MEA exhibited much smaller resistances, implying the enhanced OMT behavior of y‐Fe/NC (Figures S51 and S52 and Table S9).

Moreover, the enhanced O_2_‐accessible ASD and OMT were further verified by measuring the performance of the H_2_‐O_2_ fuel cell under varying catalyst loading (i.e., 1.5, 3.0, 4.5, 6.0, and 7.5 mg cm^−2^, Figure S53). No obvious OMT hindrance were observed in y‐ and h‐Fe/NC after increasing catalyst loading, with clear OMT resistance in s‐Fe/NC, addressing the issue of oxygen diffusion limitations caused by excessively thick catalytic layers of Fe/NC catalysts in PEMFCs. The open‐circuit voltage of the y‐Fe/NC‐based MEA reached ∼1.0 V, implying the high ORR performance of y‐Fe/NC in the H_2_‐O_2_ fuel cell. Moreover, the higher performance of y‐Fe/NC than that of h‐Fe/NC demonstrates the increased O_2_‐effective SD (Figure 5e). Based on the above discovery, the backpressure of 2 bar and loading amount of 6 mg cm^−2^ was considered as the optimized conditions. The corresponding EIS analysis demonstrates the lower Rmt of y‐Fe/NC under different reaction conditions (Figure S54). As shown in Figure 5f and Figure S55, the *P_max_* of y‐Fe/NC reached 1.03 and 0.34 W cm^−2^ in H_2_‐O_2_ and H_2_‐air fuel cell, respectively, which suppress that of s‐Fe/NC (0.69 and 0.20 W cm^−2^), h‐Fe/NC (0.86 and 0.26 W cm^−2^), and previous reports (Table S10). The y‐Fe/NC delivers the highest current density of 29.1 mA cm^−2^ at 0.9 V_iR‐free_ among all Fe/NC nanoreactors (Figure S56), approaching of commercial Pt/C (37.3 mA cm^−2^). The stability of all Fe/NC nanoreactors was evaluated at a constant cell voltage of 0.65 V for approximately 36 h (Figure 5g). The y‐Fe/NC exhibits a lower current density decay rate than its counterparts, retaining 82.4% of its initial current density after 36 h of continuous operation. Moreover, a square wave voltage cycling accelerated stress test (AST) was performed to access durability. The *P_max_* loss of y‐Fe/NC after 3000 AST cycles is 16.7% (Figure 5h), far lower than that of s‐Fe/NC (39.8%, Figure 5i). The enhanced durability is mainly attributed to the structural advantages of y‐Fe/NC, where the outer carbon shell effectively protects the inner Fe sites from demetallation, thereby suppressing Fenton reaction and the generation of hydroxyl radicals [77].

## 3 Conclusion

In this study, we successfully developed a pH‐dependent etching strategy to fabricate Fe‐N_4_ site‐dispersed nitrogen‐doped carbon nanoreactors with varied structures, specifically of solid, yolk‐shell, and hollow morphologies. Among these, the y‐Fe/NC demonstrated the most promising performance for the ORR, achieving optimized O_2_‐accessible ASD and enhanced OMT. The optimized structure facilitated enhanced catalytic activity, as evidenced by superior *E_1/2_* and *j_d_*, surpassing those of the solid and hollow counterparts. Additionally, y‐Fe/NC exhibited outstanding stability and practical applicability in PEMFC, with a competitive *P_max_*. This work highlights the critical role of nanostructural engineering in enhancing the ORR performance of Fe/NC catalysts and offers a promising approach for the design of highly efficient electrocatalysts.

## 4 Experimental Methods

### 4.1 Synthesis of Solid, Yolk‐Shell, and Hollow NC

The pH of tannic acid solution (20 mg mL^−1^) was adjusted to 11.1, 7.0, or 4.9 by using 6 m KOH solution to synthesize solid ZIF‐8@TA (s‐ZIF‐8@TA), yolk‐shell ZIF‐8@TA (y‐ZIF‐8@TA), and hollow ZIF‐8@TA (h‐ZIF‐8@TA), respectively. Then, 6 mL of the pH‐adjusted tannic acid solution was quickly poured into 10 mL ZIF‐8 dispersion solution (containing 200 mg ZIF‐8) under intense stirring for 5 min at room temperature. After centrifuging, washing with water for three times, and drying overnight, the corresponding ZIF‐8 with different nanostructures were obtained. The solid nitrogen‐doped carbon (s‐NC), yolk‐shell NC (y‐NC), and hollow NC (h‐NC) were synthesised by annealing at 900°C with a heating rate of 5°C min^−1^ for 3 h under inert atmosphere using solid ZIF‐8, yolk‐shell ZIF‐8, and hollow ZIF‐8, respectively.

### 4.2 Synthesis of s‐Fe/NC, y‐Fe/NC, and h‐Fe/NC

To synthesize solid Fe/NC (s‐Fe/NC), 60 mg of solid NC, 2 g urea, and 5.8 mg FeCl_3_·6H_2_O were ultrasonically dispersed in 50 mL EtOH for 12 h at room temperature. Subsequently, the solution was stirred for 12 h at room temperature and then evaporated to dry at 80°C. The obtained powder was heated to 900°C with a heating rate of 5°C min^−1^ and kept for 1 h under an inert atmosphere. After naturally cooling down to room temperature, s‐Fe/NC was obtained. Yolk‐shell Fe/NC (y‐Fe/NC) and hollow Fe/NC (h‐Fe/NC) were synthesized via similar procedures using yolk‐shell NC and hollow NC, respectively.

### 4.3 Electrochemical Measurements

The electrochemical measurements were carried out on a CHI 660 electrochemical station (Shanghai Chenhua, China) or a DH7003 electrochemical station (Jiangsu Donghua, China) with a rotating‐ring disc electrode (RRDE) in a conventional three‐electrode configuration at room temperature. The saturated Ag/AgCl double salt bridge reference electrode and graphite rod were used as the reference electrode and counter electrode, respectively. Prior to the test, the reference electrode was already corrected. All cells and electrodes were cleaned with aqua regia and deionized water to avoid impurities [78]. The catalyst‐coated glassy carbon rotating disk electrode (RDE, with a disk area of 0.0707 cm^2^) or the RRDE (with a disk area of 0.1257 cm^2^ and a Pt ring area of 0.1885 cm^2^) was used as the working electrode. The catalyst ink was prepared as follows: 4 mg of catalyst was dispersed in 1 mL solution containing 0.68 mL EtOH, 0.3 mL water, and 20 µL of Nafion solution via 2 h of ultrasonication to be homogeneous. The low‐rate rotational drying method (700 rpm) was used to consistently produce uniform catalyst films [60]. The loading of obtained catalysts and commercial Pt/C (20 wt.%) was 0.6 and 0.1019 mg cm^−2^, respectively. Before tests, the electrolyte was purged with pure N_2_ or O_2_ for at least 30 min to achieve N_2_/O_2_ saturation. The cyclic voltammetry (CV) tests with a scan rate of 50 mV s^−1^ and the linear sweep voltammetry (LSV) tests with a sweep rate of 10 mV s^−1^ were conducted in N_2_/O_2_‐saturated 0.1 m HClO_4_ at different rotation rates. The electrochemical impedance spectra were recorded with an AC amplitude of 5 mV rms by sweeping the frequency from 1000 kHz to ∼0.01 Hz at 12 points per decade (data collection on a logarithmic scale) in a single sine mode. All potentials of measured polarization curves were corrected with 100% *IR* compensation [79] and then converted to the reversible hydrogen electrode (RHE) using the conversion equation *E*(vs. RHE) = *E*(vs. Ag/AgCl) + 0.059 × pH + 0.196. Tafel slope was achieved from the Tafel equation [80]: *E* = *a* + *b*log(*J_k_*); where *E* is the applied potential of LSV tests, *a* is a constant, *b* is the Tafel slope, and *J_k_* is the kinetic current density. The accelerated durability tests (ADTs) were performed at room temperature in O_2_‐saturated 0.1 m HClO_4_ solution by applying potential cyclic sweeps between 0.6 and 1.0 V versus. RHE at a sweep rate of 100 mV s^−1^ for 10000 cycles.

### 4.4 Membrane Assembly Electrode Measurements

To prepare the catalyst ink, the 30 mg s‐Fe/NC, y‐Fe/NC, or h‐Fe/NC catalyst was mixed with 360 uL of Nafion solution (5%, 1100W Aldrich), isopropanol, and water, and then subjected to sonication for 3 h. The well‐dispersed ink was brushed on a piece of carbon paper (5 cm^2^), followed by drying in a vacuum at 80°C for 2 h. As for the anode and Pt/C counterpart, 60 wt.% Pt/C was used with a loading amount of ∼0.1 mgPt cm^−2^. The prepared cathode and anode with YLS layers (Toray YLS‐30T) were pressed onto the two sides of a Nafion 211 membrane (DuPont) at 130°C to obtain the MEA. The MEA was measured by a fuel cell test station (Scribner 850e) with UHP‐grade H_2_ and O_2_/Air at 353 K with 100% RH. The H_2_ and O_2_ flow rate was 1 standard liters per minute (SLPM) and 3 SLPM, respectively. AST was performed over a potential range from 0.6 to 0.9 V. The stability of the fuel cell was assessed at a constant potential of 0.65 V.

## Funding

Beijing Natural Science Foundation (Z240027), National Natural Science Foundation of China (NSFC), Zhejiang Provincial Natural Science Foundation (ZCLQN26B0301), Ningbo Yongjiang Talent Programme, Fundamental Research Funds for the Provincial Universities of Zhejiang (ZX2025000270), the Hong Kong UGC‐TRS (T23‐713/22‐R) award, the Hong Kong RGC‐EU Collaborative Programme initiative (E‐HKU701/23), and the “Hong Kong Quantum AI Lab Ltd” funded by the AIR@InnoHK.

## Conflicts of Interest

The authors declare no conflicts of interest.

## Supporting information




**Supporting File**: adma72575‐sup‐0001‐SuppMat.docx.

## Data Availability

The data supporting this article have been included as part of the Supplementary Information.
