# Supplementary material for: O2‐Accessible Fe–N4 Active Site Density Boosts Efficient Oxygen Reduction to Fuel‐Cell Level
Source: Adv Mater. 2026 Feb 16;38(16):e21600. doi: 10.1002/adma.202521600 (PMC12994327; doi:10.1002/adma.202521600)
Supplement: Supplementary file 1 — Supporting File: adma72575‐sup‐0001‐SuppMat.docx. [file ADMA-38-e21600-s001.docx]

Supporting Information

O_2_-accessible Fe–N_4_ active site density boosts efficient oxygen reduction to fuel-cell level

Author(s), Tianyu Zhang, Chen Liang, Shilun Sun, Shibo Xi, Jinliang Yuan, Zhongbin Zhuang, Zhengxiao Guo*, and Junfeng Liu*

E-mail: [ljf@mail.buct.edu.cn](mailto:ljf@mail.buct.edu.cn); zxguo@hku.hk

**Table of Contents**

[Supplementary Experimental Procedures. 5](#_Toc221578987)

[Chemicals and materials. 5](#_Toc221578988)

[Synthesis of ZIF-8. 5](#_Toc221578989)

[Supplementary Characterizations and Measurements. 5](#_Toc221578990)

[Characterization. 5](#_Toc221578991)

[X-ray absorption spectroscopy measurements. 6](#_Toc221578992)

[Electron transfer number calculation. 7](#_Toc221578993)

[Electrochemical active surface area calculation. 7](#_Toc221578994)

[Nitrite stripping measurement. 7](#_Toc221578995)

[CO pulse-chemisorption measurement. 8](#_Toc221578996)

[O_2_ temperature programmed desorption measurement. 9](#_Toc221578997)

[Activation energy calculation. 9](#_Toc221578998)

[Thiele modulus *Ф* and effectiveness factor *η* calculation*.* 10](#_Toc221578999)

[Finite element methods. 10](#_Toc221579000)

[Density functional theory calculation. 11](#_Toc221579001)

[Supplementary Figures: 13](#_Toc221579002)

[Fig. S1. Synthetic procedure illustration. 13](#_Toc221579003)

[Fig. S2. Morphological characterizations of ZIF-8. 13](#_Toc221579004)

[Fig. S3. Crystalline structure characterizations. 14](#_Toc221579005)

[Note S1. Characterizations on TA etching process. 14](#_Toc221579006)

[Fig. S4. Morphological characterizations of ZIF-8@TA. 15](#_Toc221579007)

[Fig. S5. Morphological characterizations of NC. 15](#_Toc221579008)

[Fig. S6. Morphological characterizations of Fe/NC. 16](#_Toc221579009)

[Fig. S7. HRTEM images of y-Fe/NC and h-Fe/NC. 17](#_Toc221579010)

[Fig. S8. Elemental mappings. 18](#_Toc221579011)

[Fig. S9. Oxidation state of Fe determined by XAS. 19](#_Toc221579012)

[Fig. S10. Wavelet transform analysis. 20](#_Toc221579013)

[Fig. S11. Fe K-edge EXAFS analysis of Fe foil. 21](#_Toc221579014)

[Fig. S12. Fe K-edge EXAFS analysis of FePc. 22](#_Toc221579015)

[Fig. S13. Fe K-edge EXAFS analysis of Fe_2_O_3_. 23](#_Toc221579016)

[Fig. S14. Fe K-edge EXAFS analysis of Fe/NC. 23](#_Toc221579017)

[Fig. S15. N species analysis determined by XPS. 24](#_Toc221579018)

[Fig. S16. FT-IR and Raman analysis. 25](#_Toc221579019)

[Fig. S17. DFT simulations. 25](#_Toc221579020)

[Fig. S18. ORR activity of y-Fe/NC with different loading. 26](#_Toc221579021)

[Note S2. Catalyst loading discussion on the RDE system. 27](#_Toc221579022)

[Fig. S19. RRDE measurements. 27](#_Toc221579023)

[Fig. S20. Characterizations after accelerated degradation test. 28](#_Toc221579024)

[Note S3. Koutecky-Levich equation. 28](#_Toc221579025)

[Note S4. ORR polarization mathematical model. 29](#_Toc221579026)

[Fig. S21. ORR mathematical model. 30](#_Toc221579027)

[Fig. S22. ORR polarization curve fitting. 30](#_Toc221579028)

[Note S5 Porous RDE model. 31](#_Toc221579029)

[Fig. S23. K-L plots. 32](#_Toc221579030)

[Fig. S24. Porosity analysis. 32](#_Toc221579031)

[Fig. S25. Electrochemical surface area measurement. 33](#_Toc221579032)

[Fig. S26. Adsorption character to CO species. 33](#_Toc221579033)

[Fig. S27. Nitrite stripping voltammetry of Fe/NCs. 34](#_Toc221579034)

[Note S6. Turnover frequency calculation. 35](#_Toc221579035)

[Fig. S28. Turnover frequency calculation. 36](#_Toc221579036)

[Fig. S29. Rotating speed exploration. 37](#_Toc221579037)

[Fig. S30. Oxygen mass transport pathway. 37](#_Toc221579038)

[Note S7. Oxygen transfer resistance calculation. 38](#_Toc221579039)

[Fig. S31. Oxygen partial pressure regulation. 39](#_Toc221579040)

[Note S8. Oxygen partial pressure exploration. 39](#_Toc221579041)

[Fig. S32. Reaction temperature exploration. 40](#_Toc221579042)

[Fig. S33. Thiele modulus and effectiveness factor. 40](#_Toc221579043)

[Fig. S34. Adsorption character to O_2_ species. 41](#_Toc221579044)

[Fig. S35. Local H^+^ concentration by FEM simulations. 41](#_Toc221579045)

[Fig. S36. Local O_2_ concentration by FEM simulations. 41](#_Toc221579046)

[Fig. S37. Midline concentration distribution by FEM simulations. 42](#_Toc221579047)

[Fig. S38. O_2_ flow speed by FEM simulations. 42](#_Toc221579048)

[Fig. S39. Local H^+^ concentration. 42](#_Toc221579049)

[Fig. S40. Local O_2_ concentration. 43](#_Toc221579050)

[Fig. S41. O_2_ flow speed. 43](#_Toc221579051)

[Fig. S42. Contact angle analysis. 43](#_Toc221579052)

[Fig. S43. Droplet contact angle. 44](#_Toc221579053)

[Fig. S44. Static gas bubble adhesion behaviors. 44](#_Toc221579054)

[Note S9. Aerophilicity effect on nanostructure design. 45](#_Toc221579055)

[Fig. S45. Stability test for y-Fe/NC. 45](#_Toc221579056)

[Note S10. Theoretical model of BPCC system. 46](#_Toc221579057)

[Fig. S46. Illustration of BPCC system. 46](#_Toc221579058)

[Fig. S47. Reacting O_2_ bubble behavior in BPCC. 47](#_Toc221579059)

[Fig. S48. Theoretical fitting in BPCC. 47](#_Toc221579060)

[Fig. S49. Backpressure regulation. 48](#_Toc221579061)

[Fig. S50. Open-circuit voltage. 48](#_Toc221579062)

[Fig. S51. Equivalent circuit model. 48](#_Toc221579063)

[Fig. S52. Nyquist plot analysis. 49](#_Toc221579064)

[Fig. S53. Catalyst loading regulation. 49](#_Toc221579065)

[Fig. S54. Nyquist plot analysis. 50](#_Toc221579066)

[Fig. S55. PEMFC measurement. 50](#_Toc221579067)

[Fig. S56. Kinetic properties of PEMFC. 51](#_Toc221579068)

[Supplementary Tables: 52](#_Toc221579069)

[Table S1. Metal loadings of Fe/NC determined by ICP-AES. 52](#_Toc221579070)

[Table S2. EXAFS fitting parameters at the Fe K-edge. 52](#_Toc221579071)

[Table S3. Elemental contents of Fe/NC determined by XPS. 52](#_Toc221579072)

[Table S4. Performance comparison for ORR catalysts in acid. 53](#_Toc221579073)

[Table S5. BET surface area and the pore volume of Fe/NCs. 54](#_Toc221579074)

[Table S6. Summary of the surface area of all Fe/NCs. 54](#_Toc221579075)

[Table S7. CO adsorption capacity. 54](#_Toc221579076)

[Table S8. Summary of MSD of all Fe/NCs. 54](#_Toc221579077)

[Table S9. EIS fitting results. 55](#_Toc221579078)

[Table S10. PEMFC performance comparison. 56](#_Toc221579079)

[References 57](#_Toc221579080)

Supplementary Experimental Procedures.

Chemicals and materials.

Zinc nitrate hexahydrate (Zn(NO_3_)_2_·6H_2_O; 99%), Iron(III) chloride hexahydrate (FeCl_3_·6H_2_O; 99%), tannic acid (AR), urea (99%), potassium hydroxide (KOH; 85%), perchloric acid (HClO_4_; 70%), potassium thiocyanate (KSCN; 99%), and 2-methylimidazole (2-MIM; 98%) were purchased from Shanghai Aladdin biochemical technology. Methanol (MeOH; AR), ethanol (EtOH; AR), 1-propanol (AR), sodium acetate (CH_3_COONa; AR), glacial acetic acid (CH_3_COOH; AR), and sodium nitrite (NaNO_2_; AR) were purchased from Sinopharm Chemical. Commercial Pt/C (20 wt.% Pt on carbon) and Nafion dispersion (5% w/w in water and 1-propanol) were purchased from Alfa Aesar. Standard KCl solution was purchased from Labcoms. All reagents were obtained from commercial sources and used as received without further purification.

Synthesis of ZIF-8.

Typically, 10 mmol Zn(NO_3_)_2_·6H_2_O and 80 mmol 2-MIM were dissolved in 120 mL MeOH under continuous stirring. The obtained ZIF-8 was collected by centrifugation after 12 h of reaction at room temperature. Then, ZIF-8 was washed with MeOH three times and finally dried at 60°C overnight.

Supplementary Characterizations and Measurements.

Characterization.

The morphology and crystal structure of the samples were examined by field-emission scanning electron microscopy (FESEM, Zeiss SUPRA 55), transmission electron microscopy (TEM, HT7700) and X-ray diffraction (XRD, Bruker D8) with Cu Kα radiation (*λ* = 0.154178 nm). High-angle annular dark field scanning transmission electron microscopy (HAADF-STEM) and corresponding energy dispersive X-ray spectroscopy (EDX) were performed on a Hitachi HD2700C with a probe-corrector, respectively. Fourier-transform infrared spectroscopy (FT-IR) was performed by Netzsch Geraetebau Gmbh Tensor II (Germany) with a precision of 0.005 cm^−1^. Raman spectra were recorded on a Lab Ram ARAMIS Raman spectrometer (HORIBA) at an excitation wavelength of 532 nm. Detailed chemical compositions were analysed by X-ray photoelectron spectroscopy (XPS) on an ESCALAB 250Xi photoelectron spectrometer (ThermoFisher Scientific, USA) using a monochromated Al Kα 150 W X-ray beam (1486.6 eV). All binding energies were referenced to the C *1s* peak (284.8 eV). The nanoscale pore size distribution and specific surface area of the NCGAs were measured via adsorption and desorption of N_2_ (ASAP-2460-4N) based on Brunauer-Emmett-Teller (BET) theory^[1]^ and the Barrett-Joyner-Halenda (BJH) method^[2]^. The metal concentration was measured by inductively coupled plasma-atomic emission spectroscopy (ICP-AES). The bubble and droplet contact angle on the catalyst surface was recorded by a microscope (SZX16, OLYMPUS) mounted on a high-speed CCD camera (i-SPEED 3, AOS Technologies). The illumination was achieved by a fiber optic illuminator system (CEL-TCX 250).

X-ray absorption spectroscopy measurements.

The X-ray absorption fine structure spectra (Fe K-edge) were collected at X-ray Absorption Structure (XAS) for Catalysis (XAFCA) beamline of Singapore Synchrotron Light Source (SSLS) center. The SSLS comprises a compact superconducting storage ring with 700 MeV electron energy and 4.5 Tesla magnetic field to produce synchrotron radiation with a characteristic photon energy of 1.47 keV and a characteristic wavelength of 0.845 nm. The data were collected in fluorescence excitation mode using a Lytle detector. All samples were pelletized as disks of 13 mm diameter using graphite powder as a binder. The soft XAS measurements were performed at the beamline U19 of the National Synchrotron Radiation Laboratory (NSRL, Hefei). The C K-edge, N K-edge, and Fe L_2_, L_3_-edge data are collected in total electron yield (TEY) mode, monitoring total current under a vacuum better than 10^−7^ Pa. The acquired EXAFS data were processed according to the standard procedures using the ATHENA module implemented in the IFEFFIT software packages^[3]^. The *k^3^*-weighted EXAFS spectra were obtained by subtracting the post-edge background from the overall absorption and then normalizing them to the edge-jump step. Subsequently, *k^3^*-weighted χ(*k*) data of Fe K-edge were Fourier transformed to real (R) space using a Hanning window (*d_k_*=1.0 Å^−1^) to separate the EXAFS contributions from different coordination shells. To obtain the quantitative structural parameters around central atoms, the least-squares curve parameter fitting was performed using the ARTEMIS module of the IFEFFIT software package. The following EXAFS equation was used:

$$\begin{aligned} \chi\left( k \right)=\sum_{j} \frac{N_{j}S_{0}^{2}F_{j}^{\left( k \right)}}{kR_{j}^{2}}\exp\left[ -2^{k^{2}\sigma_{j}^{2}} \right]\exp\left[ \frac{-2R_{j}}{\lambda\left( k \right)} \right]\sin\left[ 2k^{R_{j}+\phi_{j}^{\left( k \right)}} \right]\# \mathrm{SEQ}\mathrm{EQ}1 \end{aligned}$$

Where *S_0_^2^* is the amplitude reduction factor; *F_j_(k)* is the effective curved-wave backscattering amplitude; *N_j_* is the number of neighbours in the *j_th_* atomic shell; *R_j_* is the distance between the X-ray absorbing central atom and the atoms in the *j_th_* atomic shell (backscatter); *λ(k)* is the mean free path in Å; *ϕ_j_(k)* is the phase shift (including the phase shift for each shell and the total central atom phase shift); *σ_j_* is the Debye-Waller parameter of the *j_th_* atomic shell (variation of distances around the average *R_j_*)^[4]^. The functions *F_j_(k)*, *λ(k)*, and *ϕ_j_(k)* were calculated with the ab initio code FEFF8.0^[5]^. For Wavelet Transform (WT) analysis, the *χ(k)* exported from Athena was imported into the Hama Fortran code^[6]^. The parameters were used as: R range, 1-4 Å, k range, 0-13 Å^−1^; *k* weight: 2; Morlet function with *κ*=10, *σ*=1 was used as the mother wavelet to provide the overall distribution^[7]^.

Electron transfer number calculation.

The number of electron transfer (n) and the percent of H_2_O_2_ were calculated by the following equations^[8]^:

$$\begin{aligned} H_{2}O_{2}\left( \% \right)=200\times\frac{\frac{i_{r}}{N}}{i_{d}+\frac{i_{r}}{N}}\# SEQ EQ 2 \end{aligned}$$

$$\begin{aligned} n=4\times\frac{i_{d}}{i_{d}+\frac{i_{r}}{N}}\# SEQ EQ 3 \end{aligned}$$

Where *i_r_* is the ring current, *i_d_* is the disk current, and N (0.424) is the collection efficiency of the Pt ring. ORR results were presented after subtracting the currents measured in N_2_-saturated 0.1 M HClO_4_ solution to remove capacitive currents.

Electrochemical active surface area calculation.

Double-layer capacity (*C_dl_*) estimates the electrochemical active surface area (ECSA) of the catalyst. Consequently, the electrochemical capacitance was assessed via cyclic voltammetry in the potential range of 1-1.2 V *vs.* RHE. The scan rates were 10-60 mV s^−1^. The *C_dl_* was estimated by plotting the 1/2∆*j* = (*j_a_* − *j_c_*) at 1.1 V *vs.* RHE (where *j_c_* and *j_a_* are the cathodic and anodic current densities, respectively) against the scan rate, in which the slope was *C_dl_*. By taking into consideration the specific capacities of a smooth planar surface with a real 1.0 cm^2^ surface area and specific capacities (*C_s_*) of 20 to 60 µF cm^−2^ in general. Here, the ECSA is calculated by using the midpoint common capacitance of 40 μF cm^−2^ as^[9]^:

$$\begin{aligned} ECSA=\frac{C_{dl}}{C_{s}}=\frac{C_{dl}}{{40 \mu F cm}^{-2} per {cm}_{ECSA}^{2}}\# SEQ EQ 4 \end{aligned}$$

Nitrite stripping measurement.

The gravimetric active mass site density (MSD) of the catalysts was obtained *via* electrochemical nitrite stripping (NS) experiments on RRDE. The catalyst loading was fixed at 0.6 mg cm^−2^ and 0.5 M acetate buffer from CH_3_COONa and CH_3_COOH at pH 5.2 was utilized as electrolyte. Briefly, CV measurements were performed in N_2_- or O_2_- saturated acetate buffer. Then the catalysts were poisoned by NaNO_2_, and nitrite stripping was carried out in the region of 0.4 to −0.3V *vs.* RHE. CV curves measured in Ar-saturated acetate buffer and RDE polarization curves tested in O_2_-saturated acetate buffer were recorded before, during, and after the nitrite absorption. It is worth noticing that the current integrator is crucial to correctly measure the charges during stripping measurements. The electrochemical nitrite reduction stripping charge on Fe−N_x_ sites (Q_strip_, excess coulometric charge associated with the stripping peak) could be proportional to the MSD before and after nitrite absorption, based on the assumption on the adsorption and stripping process in Fig. S26. The accessible Fe−N_4_ sites can be strongly interact with the nitrite anions to form stable poisoned adducts. Thus, we can propose that the active site number is equal to the adsorbed nitrite anion number. Since per Fe−N_4_ site can be stripped at a very low potential to completely recover the catalytic activity with a transfer of 5 electrons per nitrite anion, we can calculate the MSD accordingly from the following equations^[10]^:

$$\begin{aligned} MSD(ns)\left( site g^{-1} \right)=\frac{Q_{strip}\times N_{A}}{n_{strip}\times F\times m_{cat}}\# SEQ EQ 5 \end{aligned}$$

Where *n_strip_* (= 5) is the number of electrons associated with reducing one adsorbed nitrosyl per FeN_4_ site, *F* is the faradaic constant, *N_A_* is Avogadro`s constant, *m_cat_* is the mass of the catalyst (*cat*), and the loading of *cat* is 0.27 mg cm^−2^.

CO pulse-chemisorption measurement.

The CO pulse-chemisorption experiments for the obtained catalysts were conducted on a Micromeritics Autochem II 2920 instrument equipped with a thermal conductivity detector (TCD). 100 mg of catalyst was inserted between two pieces of quartz wool at the bottom of the internal quartz bulb and pre-treated to remove strongly adsorbed species on metal-based sites on the surface, especially O_2_. The pre-treatment involves purging the lines with He (20 cm^3^ min^−1^ for 30 min), followed by annealing at 600 °C for 15 min with a heating rate of 10°C min^−1^, and followed by cooling to room temperature. Pulse chemisorption at −80°C (dry ice and acetone) consisted of 10 min He line flushing (20 cm^3^ min^−1^), followed by six consecutive CO pulses injected by the automated sample loop in intervals of 25 min. The CO cryo adsorption reached saturation after three pulses for all Fe/NC. The discrepancy in peak areas (*∆A*), representing adsorbed molar CO amount, is calculated based on six individually baseline-corrected integral pulse areas A_1_ to A_6_ according to:

$$\begin{aligned} \Delta A=\frac{A_{4}+A_{5}+A_{6}}{3}-\sum_{k=1}^{3} A_{k}\# SEQ EQ 6 \end{aligned}$$

The calibration constant *c_co_* was derived as 4.2 × 10^−7^ mmol per unit area by injecting a specific volume of CO gas, which was subsequently employed to convert integral peak areas to molar CO amounts. We can calculate the CO chemisorption active site density (MSD(CO)) accordingly from the following equations:

$$\begin{aligned} MSD(CO)\left( site g^{-1} \right)=\frac{c_{CO}\times\Delta A\times N_{A}}{m_{cat}}\# SEQ EQ 7 \end{aligned}$$

Where *c_co_* is the calibration constant, *∆A* is the peak area discrepancy, *N_A_* is Avogadro`s constant and *m_cat_* is the mass of the catalyst inserted in the quartz tube of the chemisorption reactor.

O_2_ temperature programmed desorption measurement.

The O_2_ temperature programmed desorption (TPD) experiments for the obtained catalysts were also conducted on the Micromeritics Autochem II 2920 instrument equipped with a mass spectrometry (MS) detector. The catalyst was pretreated with He ambient at 150 °C for 1 h to remove the adsorbed gaseous impurities. Then, the samples were cooled down to 50 °C and treated with pure O_2_ before the TPD test. And then the carrier gas was changed to He and the temperature was increased to 300 °C at a rate of 10 °C min^−1^ while the MS signal was recorded.

Activation energy calculation.

The activation energy (E_a_) was evaluated at different fixed potentials using the Arrhenius Equation in the temperature range 20-60 °C^[11]^. To show the effect of the ORR kinetics and O_2_ concentration, the current density measured at different temperatures has been corrected according to:

$$\begin{aligned} j_{m}^{*}=j_{m}\cdot exp\left( \frac{1 mM}{C_{O_{2}}} \right)^{0.79}\# SEQ EQ 8 \end{aligned}$$

Where *j_m_* is the measured mass-transport corrected current density, C_O2_ is the actual O_2_ concentration in the liquid electrolyte at the temperature at which the measurement was done^[12]^, and *j_m_^*^* is the corresponding current density for an oxygen concentration of 1 mM. Then, the Arrhenius plot (logarithm of current density versus the inverse of the absolute temperature) can be drawn, and *E_a_* can be calculated from the linearization of the Arrhenius law:

$$\begin{aligned} j_{m}^{*}\left( T \right)=j_{m}^{*}\left( T_{\infty} \right)\cdot exp\left( \frac{-E_{a}}{2.3RT} \right)^{0.79}\# SEQ EQ 9 \end{aligned}$$

Where *T* is the temperature at which the measurement was done, and *j_m_^*^*(*T*_∞_) is a constant value. The slope of the Arrhenius plots is equal to −*E_a_*/(2.3*R*).

Thiele modulus *Ф* and effectiveness factor *η* calculation*.*

The Thiele modulus *Ф* reflects the influence degree of internal diffusion and chemical reactions during the reaction^[13]^. The effectiveness factor *η* is used to evaluate the utilization efficiency of catalysts and can be derived from *Ф*. *Ф* is defined as:

$$\begin{aligned} Ф=\sqrt{\frac{r_{intrinsc}}{r_{diffusion}}}=\frac{\sqrt{n+1}}{2}R_{p}\sqrt{\frac{k{C_{as}}^{n-1}}{D_{eff}}}\# SEQ EQ 10 \end{aligned}$$

Where *Ф* is the Thiele modulus, intrinsic is the maximum reaction rate, diffusion is the maximum diffusion rate, *n* is the reaction order, *R_p_* is the diameter of the catalyst pellet, *k* is the reaction rate constant, *C_as_* is the surface reactant concentration and *D_eff_* is the effective diffusivity inside the catalyst tunnel. Thus, *Ф* can be calculated by:

$$\begin{aligned} \frac{E_{app,a}}{E_{int,a}}=\frac{1}{2}+Ф\frac{1-{tanh}^{2}Ф}{2tanhФ}\# SEQ EQ 11 \end{aligned}$$

Where *E_app,a_* is the apparent activation energy and *E_int,a_* is the intrinsic activation energy. The effectiveness factor *η* was used to evaluate pellet utilization which can be calculated when the shape of the catalysts is nearly spherical:

$$\begin{aligned} \eta=\frac{1}{Ф}\left[ \frac{1}{tanh3Ф}-\frac{1}{3Ф} \right]\# SEQ EQ 12 \end{aligned}$$

A low *η* means that internal diffusion seriously affects the reaction process and there is a large unused area inside the catalysts.

Finite element methods.

The finite element simulation and analysis (FEA) were performed with Free FEM software^[14]^. According to the experimental data, all the model was constructed as a regular hexagon with a side length of 231 nm and a pore size of 10 nm. Subsequently, a shell with a thickness of 15 nm was constructed to form yolk-shell and hollow models. Finally, a sphere with a diameter of 200 nm and a pore size of 10 nm was added to the center of the yolk-shell model to finish the modelling process. The error of the diameter in the models was ±0.5 nm. The 2D axisymmetric simplification method was employed based on the characteristics of the imitation model, and the diffusion and reaction were only carried out within the rotating sweeping plane. Moreover, the catalyst layers using s-, y-, and h-Fe/NC were assumed to have the same thickness with closely arranged hexagons and a total of 5*9 units employed in the simulation.

The speed field was calculated by the Navier–Stokes equations:

$$\begin{aligned} \nabla\cdot\left( \rho\cdot u \right)=0\# SEQ EQ 13 \end{aligned}$$

$$\begin{aligned} \rho\cdot\frac{\partial u}{\partial t} + \rho\cdot\left( u\cdot\nabla)\cdot u \right)=\nabla\cdot\left[ - P\cdot I+\tau\right]+F+\rho\cdot g\# SEQ EQ 14 \end{aligned}$$

$$\begin{aligned} \tau= u\cdot(\nabla\cdot u+\left( \nabla\cdot u \right)^{T}\#\# SEQ EQ 15 \end{aligned}$$

$$\begin{aligned} F= \sigma\cdot k\cdot n\cdot\delta\#\# SEQ EQ 16 \end{aligned}$$

Where ∇ is the divergence, *ρ* is the mass density, *u* is the velocity vector, *t* is the time, *P* is the pressure, *I* is the identity matrix, *τ* is the viscous stress tensor, *F* is the volume force vector, *g* is the body accelerations acting on the continuum, *T* is the temperature, *σ* is the surface tension coefficient, *k* is the interfacial curvature, *n* is the interfacial unit normal vector, *δ* and is the delta function centered at the interface.

Utilizing the calculated convective velocity, the mass conservation analysis on the adsorbate concentration was conducted, meeting the criteria for a dilute species transfer interface simulated with the diffusion equation:

$$\begin{aligned} \nabla\cdot\left( -D\cdot\nabla\cdot c \right)+u\cdot\nabla\cdot c=0\# SEQ EQ 17 \end{aligned}$$

Where ∇ is the divergence, *D* is the diffusivity, *c* is the concentration, and *u* is the velocity vector. It satisfies Fick's diffusion law as a whole.

The calculated domain was simulated *via* filling the electrolyte in a square region with a side length of 800 nm. It is worth noticing that we defined the obtained H_2_O from the ORR reaction as a substance different from solution water, thus the product distribution around different Fe/NC nanoreactors can be easily compared. All the involved reactions are surface reactions:

$$\begin{aligned} J=k{\cdot c}_{H}\cdot c_{o_{2}}\# SEQ EQ 18 \end{aligned}$$

Where J is the reaction rate, k is the surface reaction frequency, and c is the concentration. The oxygen was poured from the left boundary and outflowed from the right boundary. The inflow velocity was set to 100 nm s^−1^ at the entrance, the concentration of HClO_4_ was set to 0.1 mol L^−1^, the concentration of O_2_ was set to 1.0 mol L^−1^, and the diffusion coefficient was set to 1 × 10^−13^ m^2^ s^−1^.

Density functional theory calculation.

All theoretical calculations were performed using density functional theory (DFT)^[15]^. The exchange-correlation potential was described by the generalized-gradient approximation (GGA) with spin-polarized Perdew-Burke-Ernzerhof (PBE) functional. The reaction kinetics and thermodynamics were analyzed based on spin-polarized density functional theory calculations with van der Waals corrections (DFT-D3), which provide substantial accuracy in describing the chemisorption and physisorption properties of adsorbates. The k-point samplings were set to 3×3×1 for geometry optimizations and 5×5×1 for electronic structure computations. All atoms were relaxed using a cutoff energy of 450 eV until the force reaches a convergence threshold of 0.01 eV/Å. To module the Fe atoms hosted in mesopores and voids, a Fe–N_4_ site is embedded in a periodic 7×7 flat graphene (114 carbon sites) support with lattice parameters a = b = c = 17 Å, donated as FeN_4_-C. To module the Fe atoms hosted in micropores, a Fe–N_4_ site is embedded in a carbon sphere (180 carbon sites) support with diameter of 1.5 nm and lattice parameters a = b = c = 17 Å, donated as FeN_4_-C_180_. The convergence threshold for the self-consistent field (SCF) was set at 10^5^ eV for the total energy change and 0.01 eV/Å for the maximum forces on atoms. The binding energy was calculated by subtracting the energies of the isolated adsorbate and the catalyst from the total energy of the adsorbed system: E_b_ = E_(slab + absorbate)_ – E_(slab)_ – E_(adsorbate)_.

Supplementary Figures:


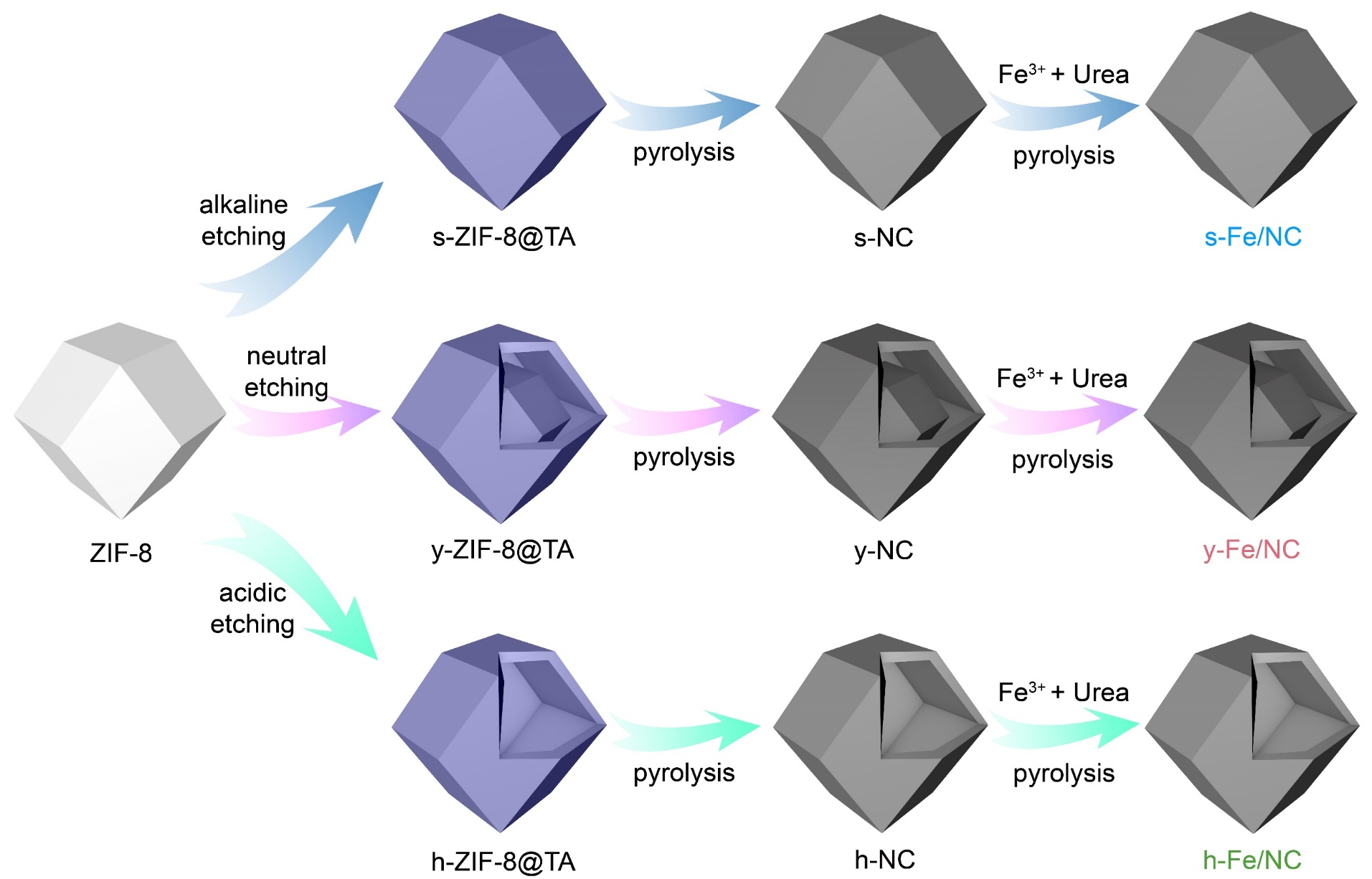


Fig. S1. Synthetic procedure illustration.

Schematic illustration of the synthetic procedure of solid (s-), yolk-shell (y-), and hollow (h-) Fe/NC nanoreactors.


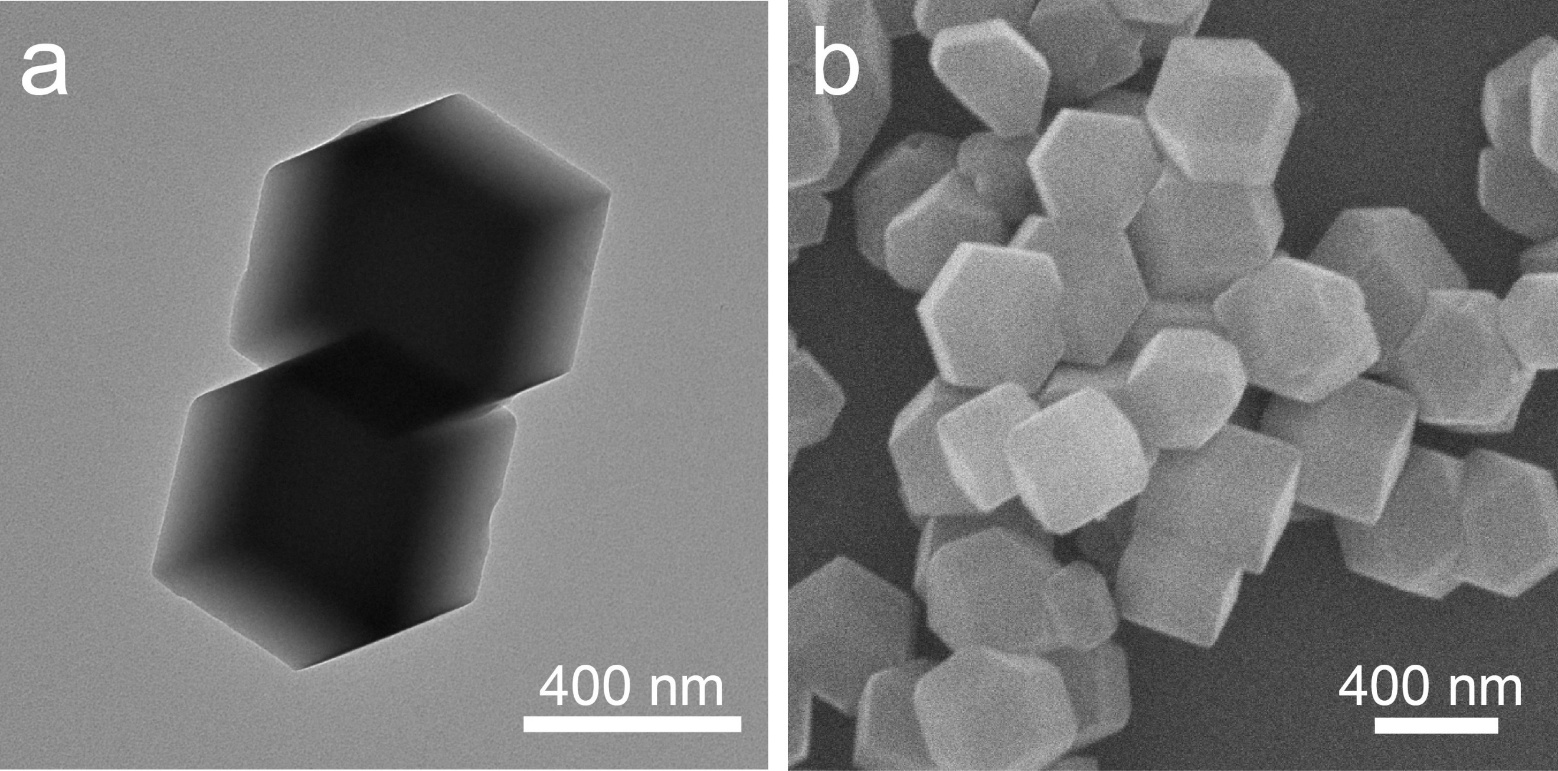


Fig. S2. Morphological characterizations of ZIF-8.

(a) TEM and (b) SEM images ZIF-8. The obtained ZIF-8 nanoparticles exhibit a typical rhombic dodecahedron shape with an average size of ~400 nm.


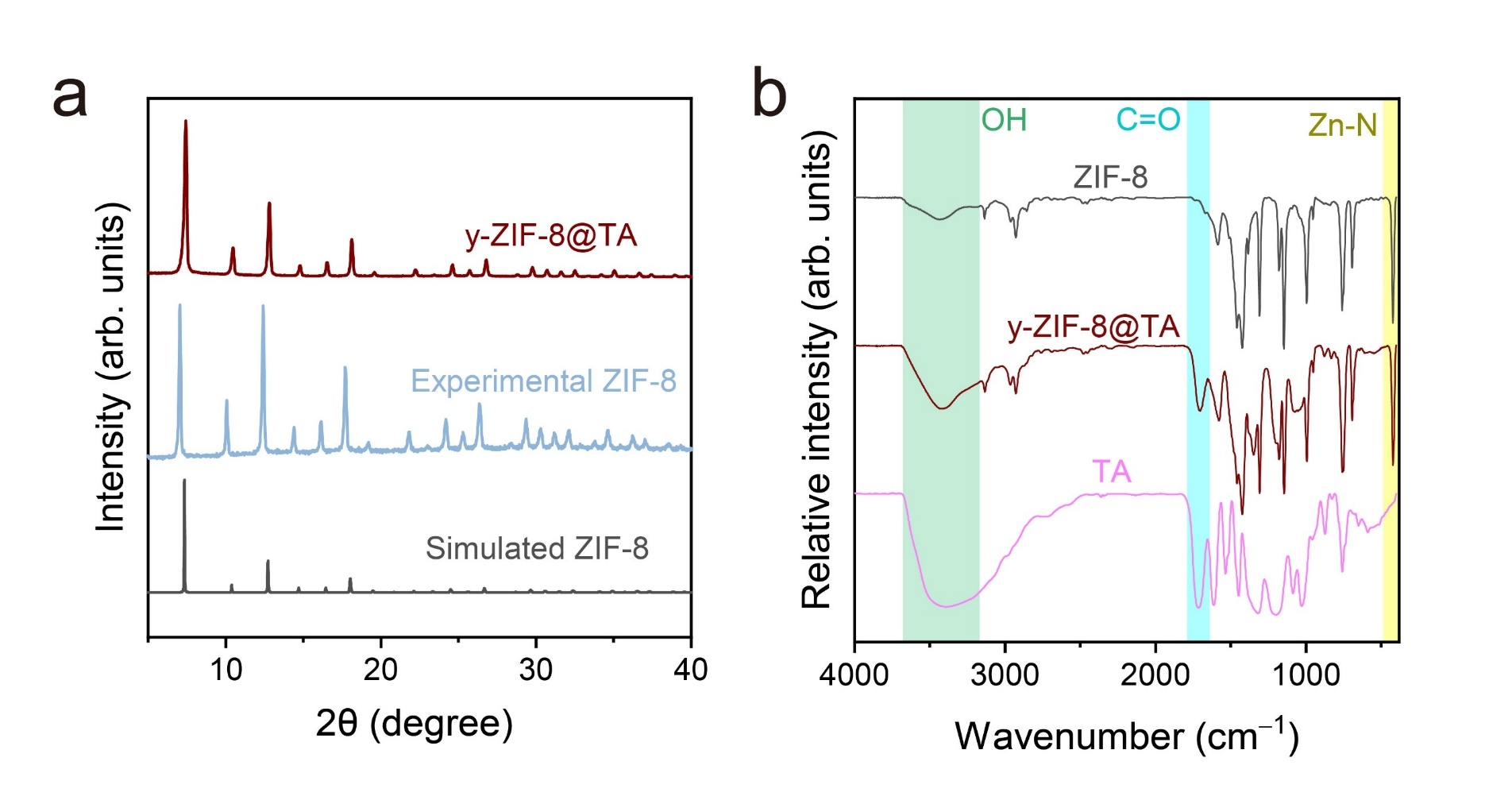


Fig. S3. Crystalline structure characterizations.

1. (a) XRD patterns of simulated ZIF-8, experimental ZIF-8, and y-ZIF-8@TA. (b) FT-IR spectra of ZIF-8, TA and y-ZIF-8@TA.

Note S1. Characterizations on TA etching process.

XRD was conducted to study the crystallographic structure transformation during the etching procedure. ZIF-8 structures were well-preserved after TA etching. However, the diffraction peak intensities of y-ZIF-8@TA were slightly reduced compared to those of pristine ZIF-8, indicating partial amorphization during TA etching. The FT-IR spectra further support the TA etching process. The peaks at 1705 and 3326 cm^−1^ in y-ZIF-8@TA can be attributed to the stretching vibration of C=O and polyphenolic hydroxyl (OH) moiety, respectively, indicating that ZIF-8 nanoparticles were covered with a metal-phenolic network film and TA molecules^[16]^.


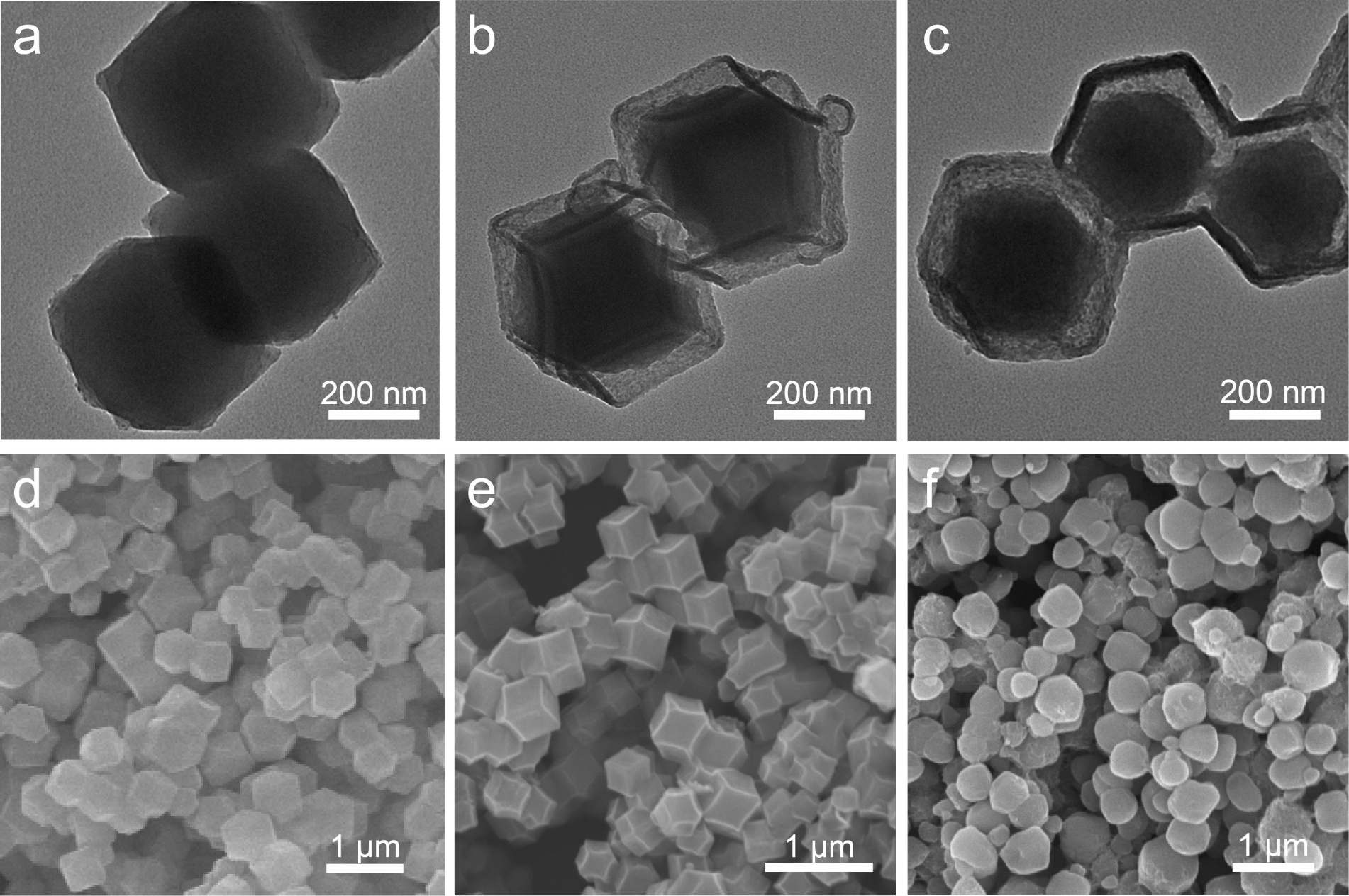


Fig. S4. Morphological characterizations of ZIF-8@TA.

(a-c) TEM and (d-f) SEM images of (a, d) s-ZIF-8@TA, (b, e) y-ZIF-8@TA and (c, f) h-ZIF-8@TA.


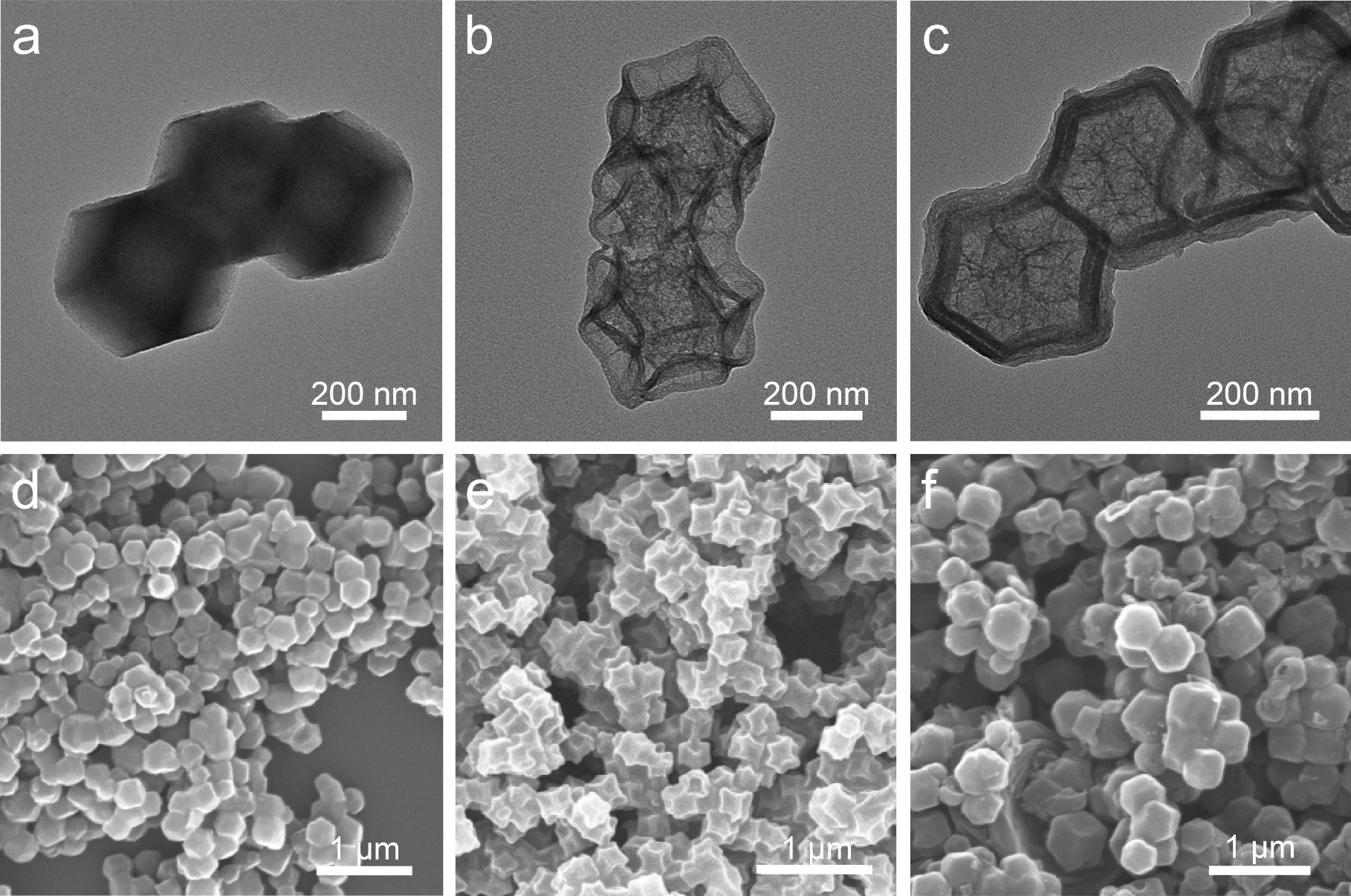


Fig. S5. Morphological characterizations of NC.

(a-c) TEM and (d-f) SEM images of (a, d) s-NC, (b, e) y-NC and (c, f) h-NC.


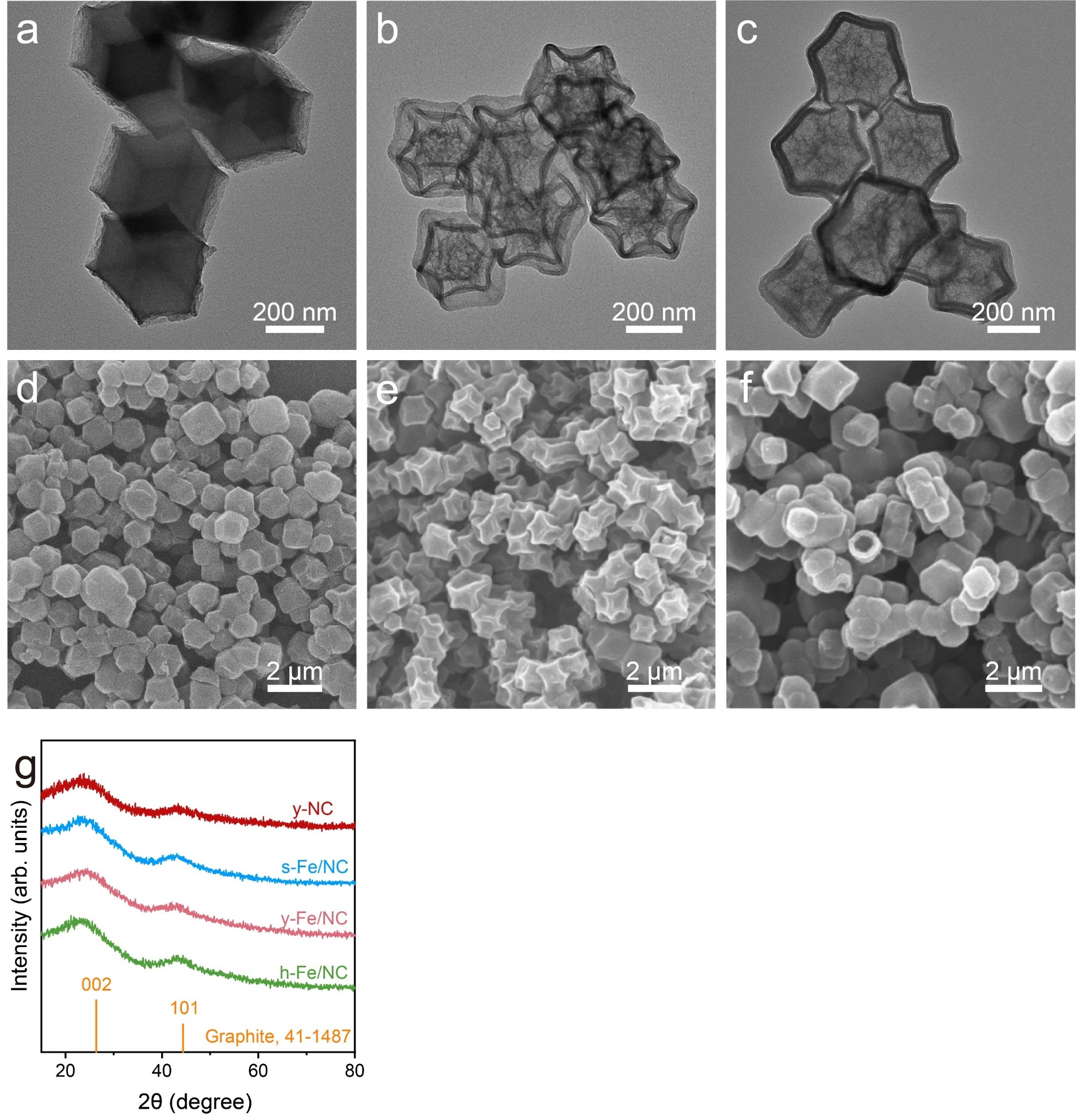


Fig. S6. Morphological characterizations of Fe/NC.

(a-c) TEM and (d-f) SEM images of (a, d) s-Fe/NC, (b, e) y-Fe/NC and (c, f) h-Fe/NC. (g) XRD patterns of y-NC, s-Fe/NC, y-Fe/NC, and h-Fe/NC.


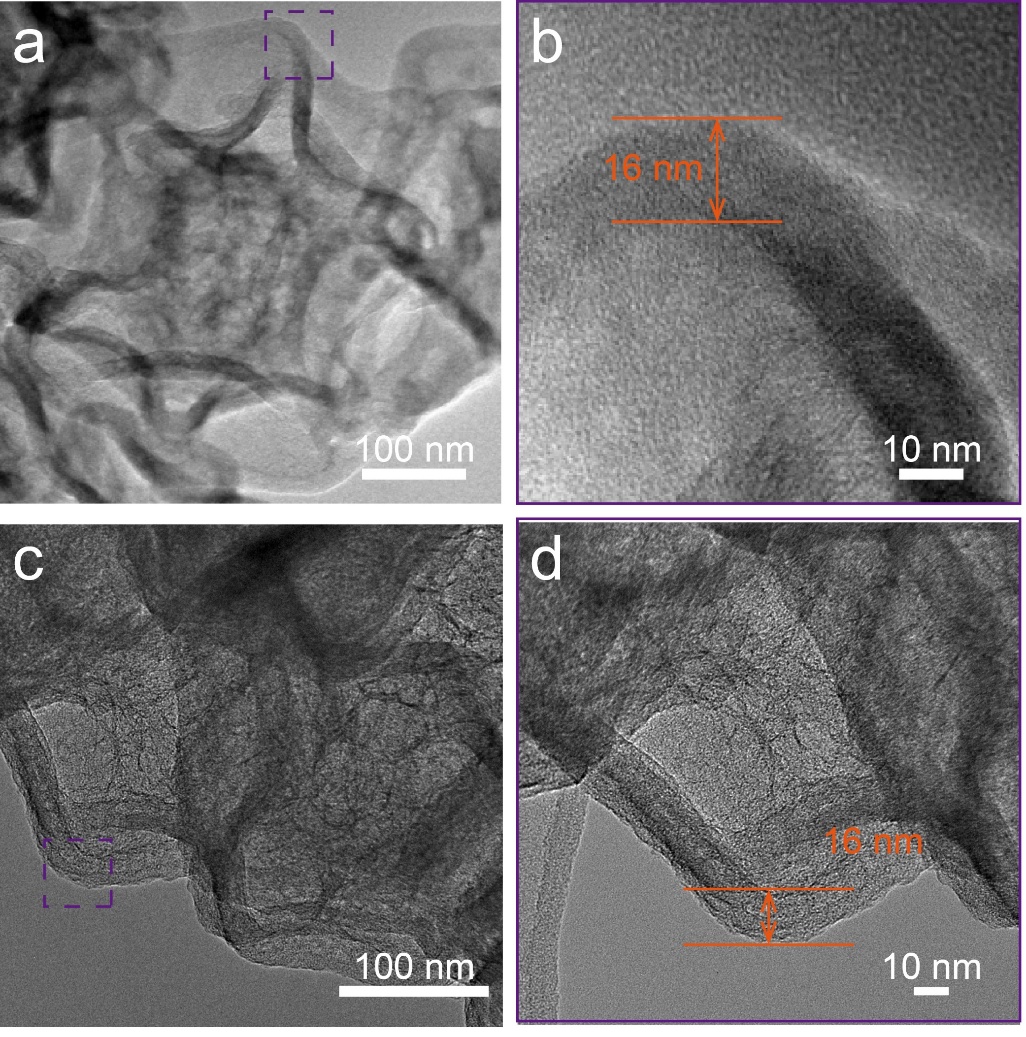


Fig. S7. HRTEM images of y-Fe/NC and h-Fe/NC.

(a) Overview of the y-Fe/NC particle and (b) zoom in the area marked with a purple square in a. The core size is about 200 nm and the thickness of the carbon shell in y-Fe/NC is measured as ~16 nm. (c) Overview of the h-Fe/NC particle and (d) zoom in the area marked with a purple square in c. The thickness of the carbon shell in h-Fe/NC is measured as ~16 nm.

**
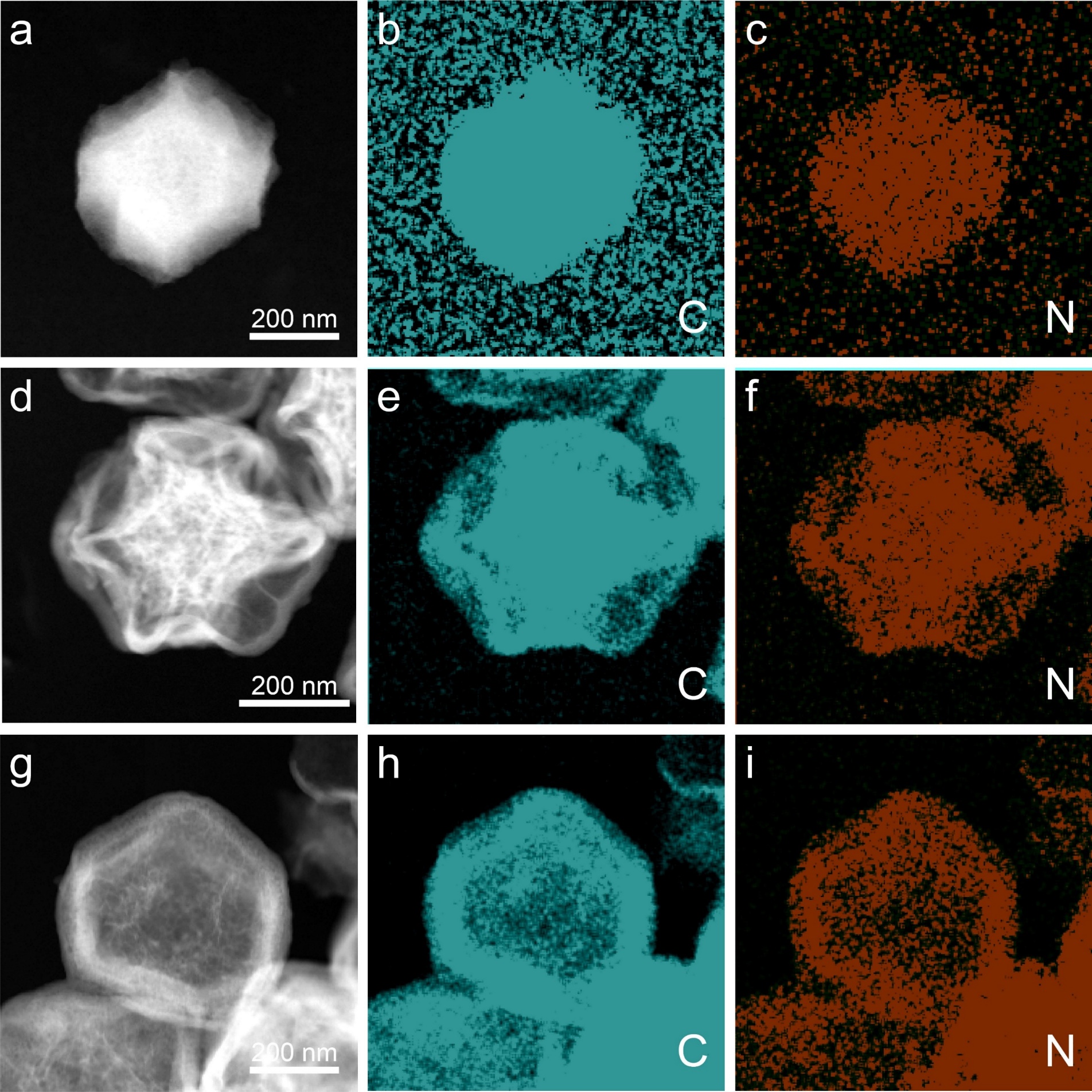
**

Fig. S8. Elemental mappings.

(a, d, g) TEM, (b, e, g) C, and (c, f, i) N for (a-c) s-Fe/NC, (d-f) y-Fe/NC, and (g-i) h-Fe/NC, respectively.


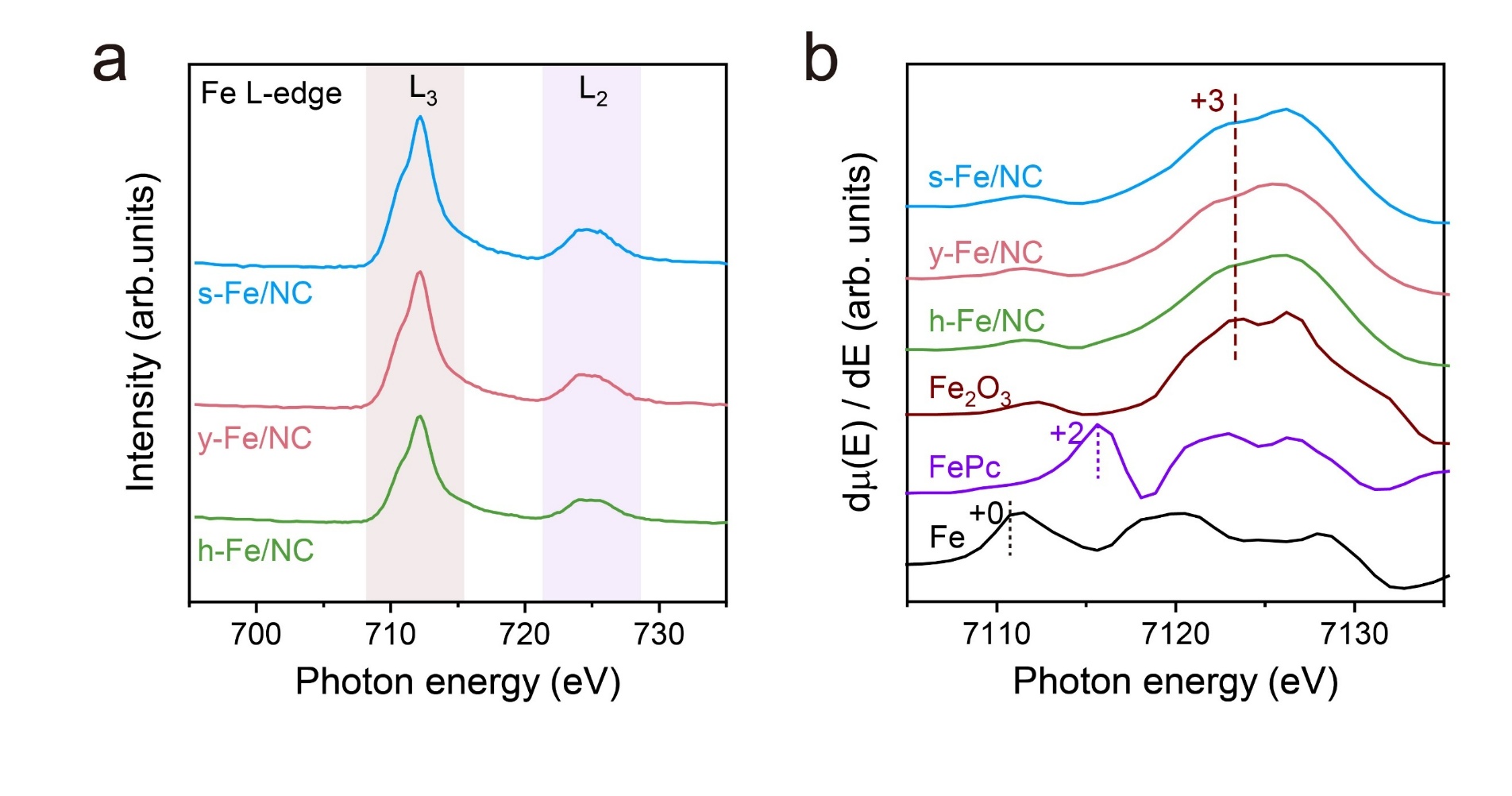


Fig. S9. Oxidation state of Fe determined by XAS.

(a) Fe L-edge XANES spectra for s-Fe/NC, y-Fe/NC, and h-Fe/NC. (b) First derivatives of the Fe K edge XAS data (in Fig. 1g) for s-Fe/NC, y-Fe/NC, and h-Fe/NC samples (nominal valence of +3) and the reference Fe foil (0), FePc (+2) and Fe_2_O_3_ (+3).


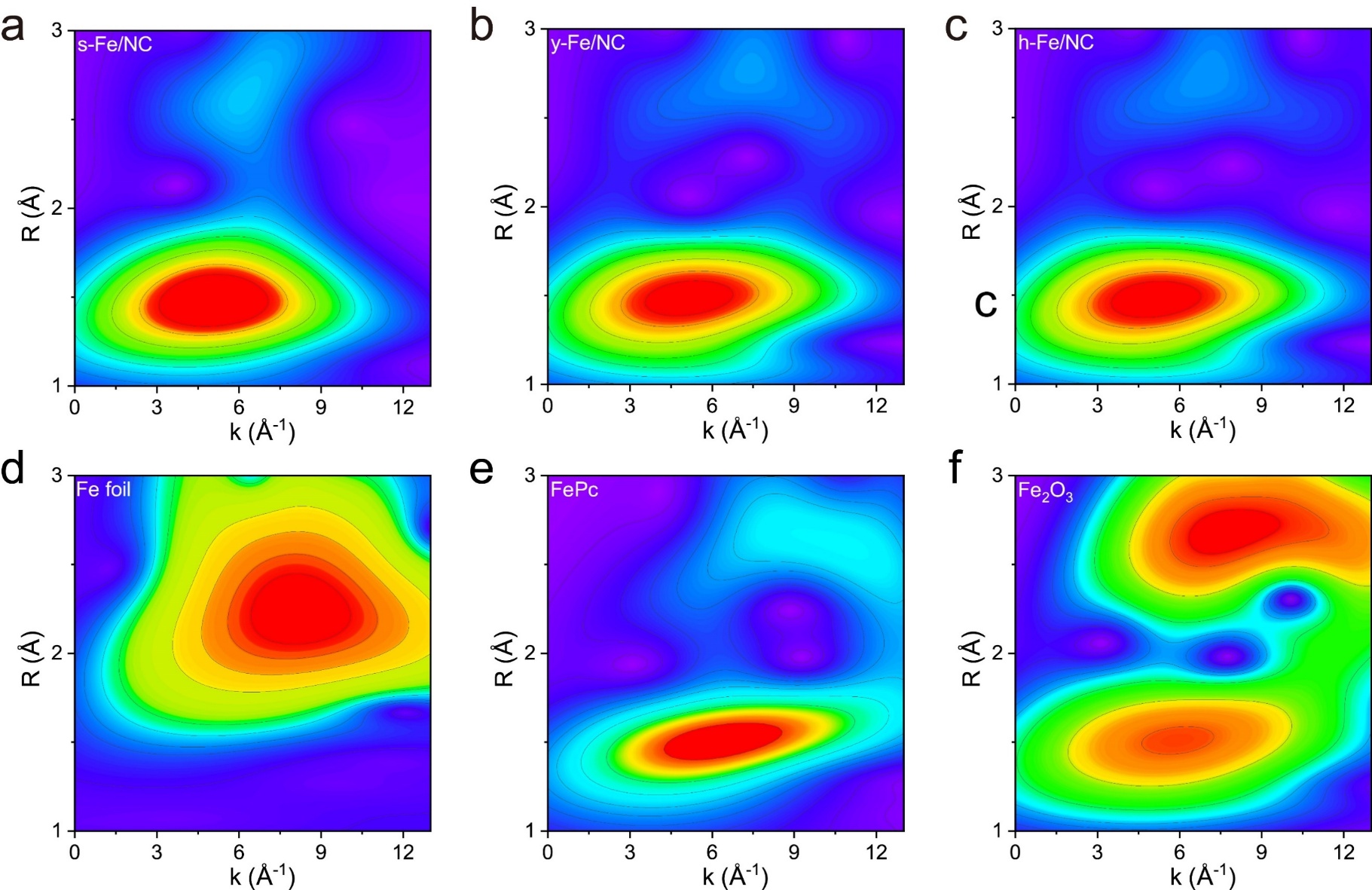


Fig. S10. Wavelet transform analysis.

WT of the *k^3^*-weighted Fe K-edge EXAFS signals of (a) s-Fe/NC, (b) y-Fe/NC, (c) h-Fe/NC, (d) Fe-foil, (e) FePc, (f) Fe_2_O_3_, based on Morlet wavelets with optimum resolutions at 2.0 Å. Utilizing wavelet transforms (WT) analysis on EXAFS spectra is an effective strategy for distinguishing the combined contributions that arise from distinct neighboring atoms or scattering events in both K- and R-spaces. The Fe sites in the obtained Fe/NC samples are once again confirmed to be mononuclear center due to the absence of WT signal at high k value over ~8.0 Å^−1^.


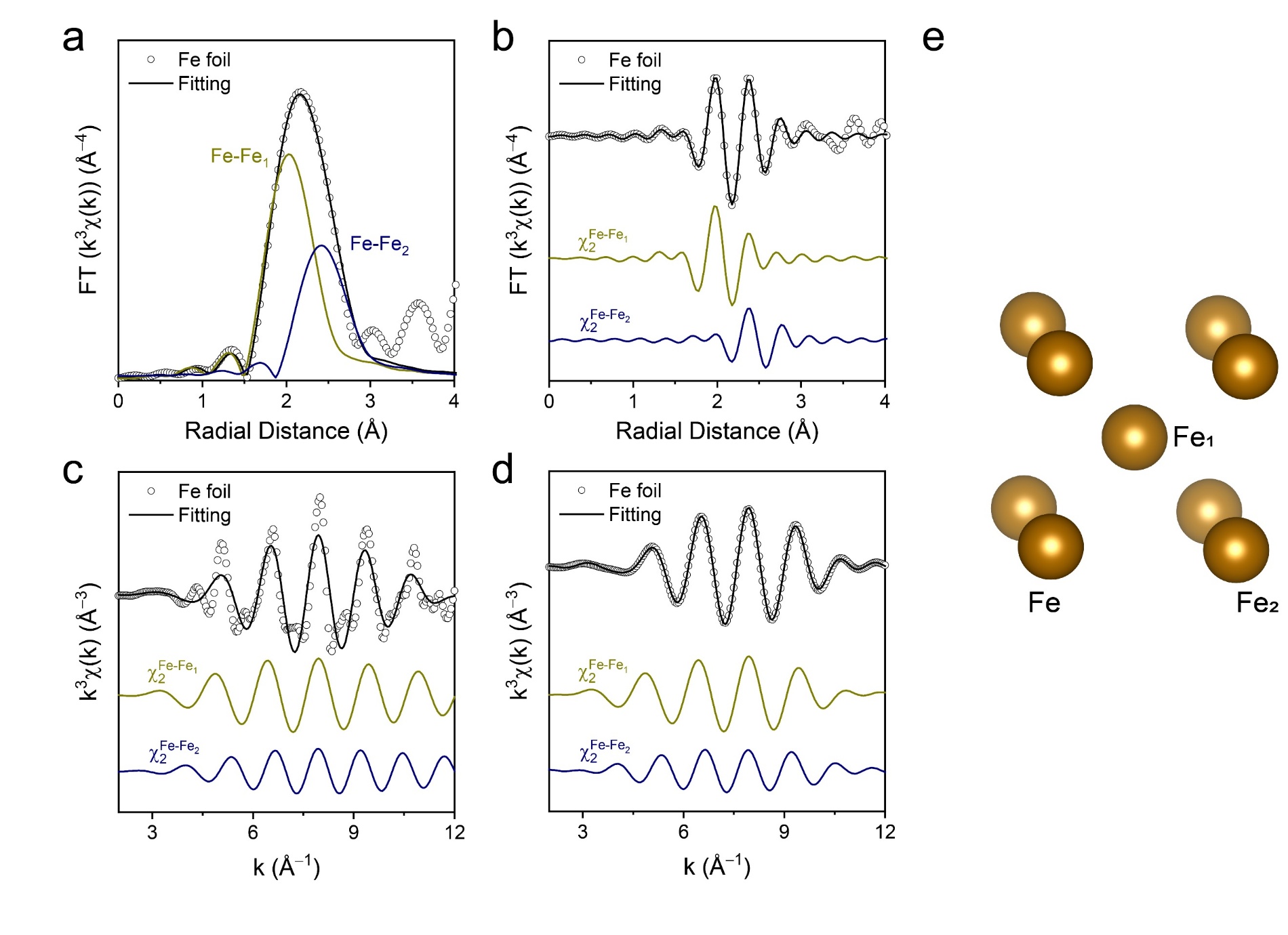


Fig. S11. Fe K-edge EXAFS analysis of Fe foil.

(a) R space, (b) real R space, (c) k space, and (d) q space for Fe foil. The data are k^3^-weighted and not phase-corrected. (e) The schematic model of Fe: Fe (dark yellow).


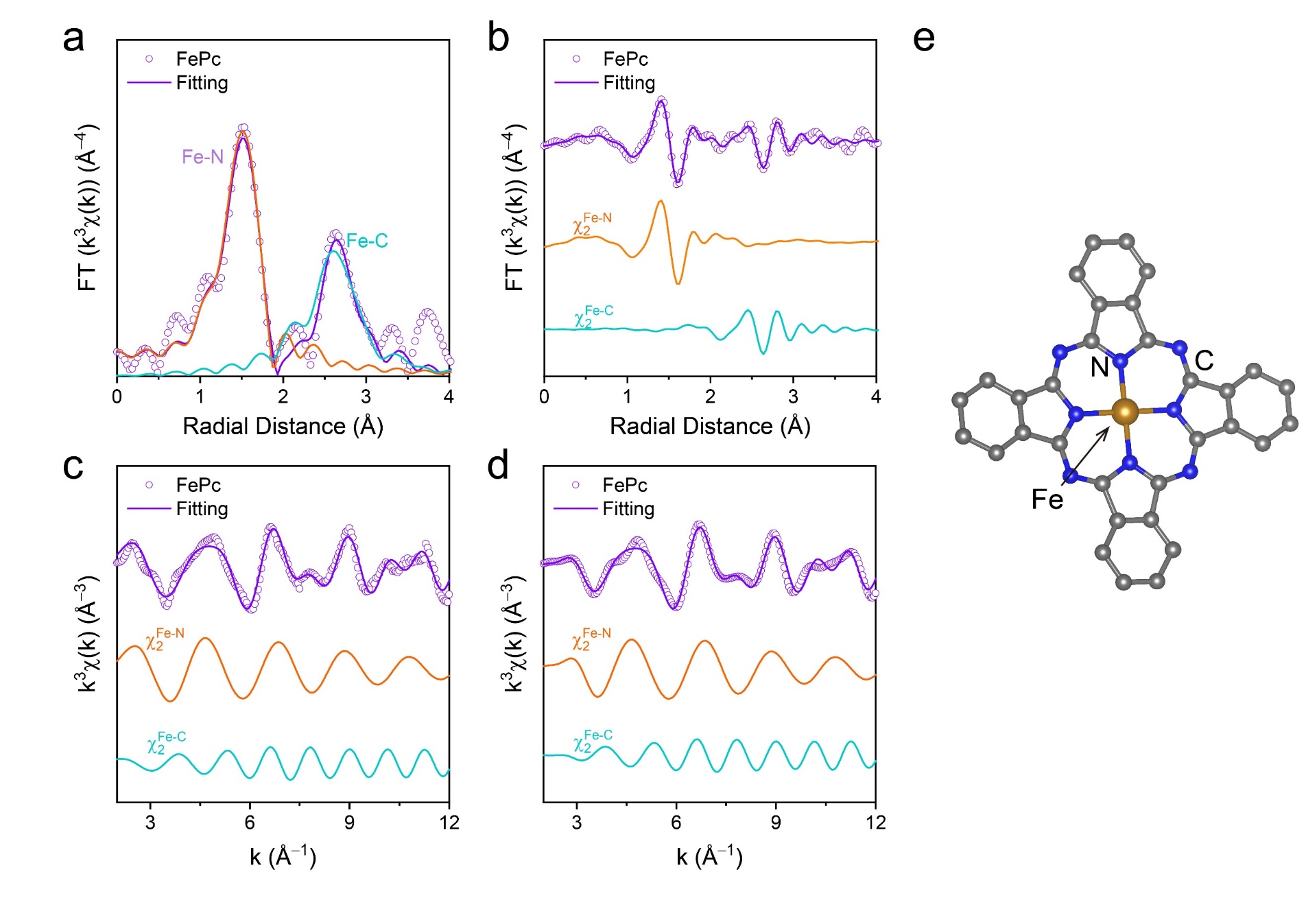


Fig. S12. Fe K-edge EXAFS analysis of FePc.

(a) R space, (b) real R space, (c) k space, and (d) q space for FePc. The data are k^3^-weighted and not phase-corrected. (e) The schematic model of FePc: Fe (dark yellow), N (blue), C (gray).


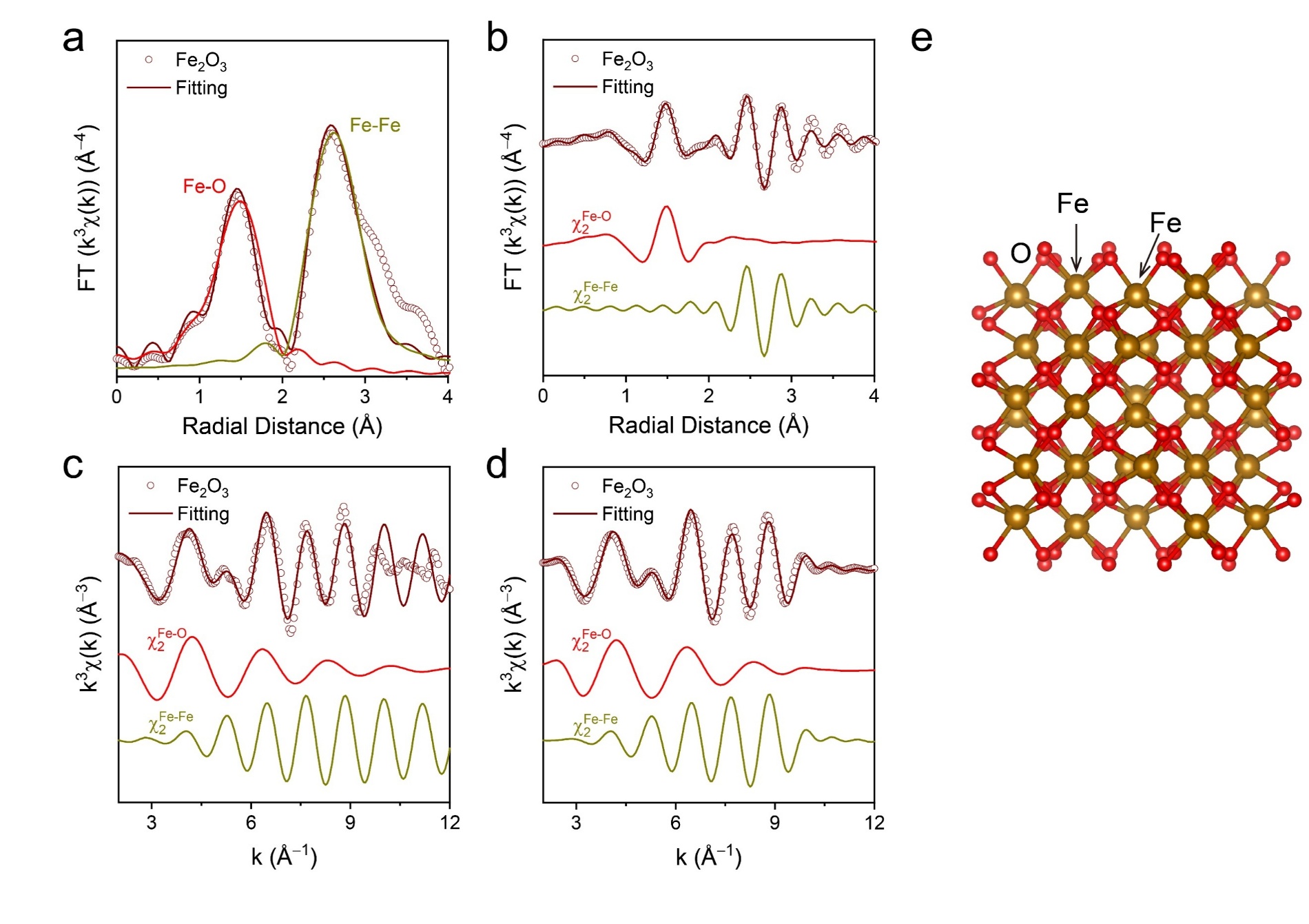


Fig. S13. Fe K-edge EXAFS analysis of Fe_2_O_3_.

(a) R space, (b) real R space, (c) k space, and (d) q space for Fe_2_O_3_. The data are k^3^-weighted and not phase-corrected. (e) The schematic model of Fe_2_O_3_: Fe (dark yellow), O (red).

**
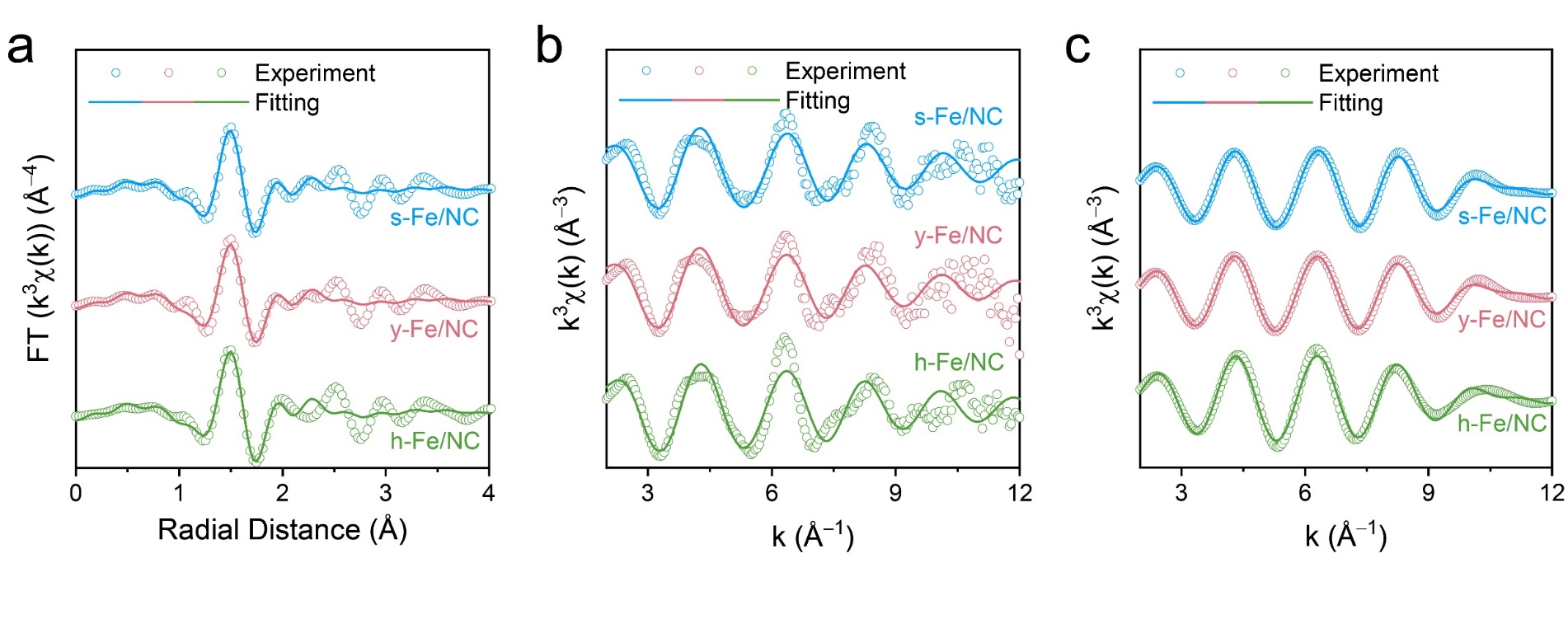
**

Fig. S14. Fe K-edge EXAFS analysis of Fe/NC.

(a) real R space, (b) k space, and (c) q space for s-Fe/NC, y-Fe/NC, and h-Fe/NC. The data are k^3^-weighted and not phase-corrected.

**
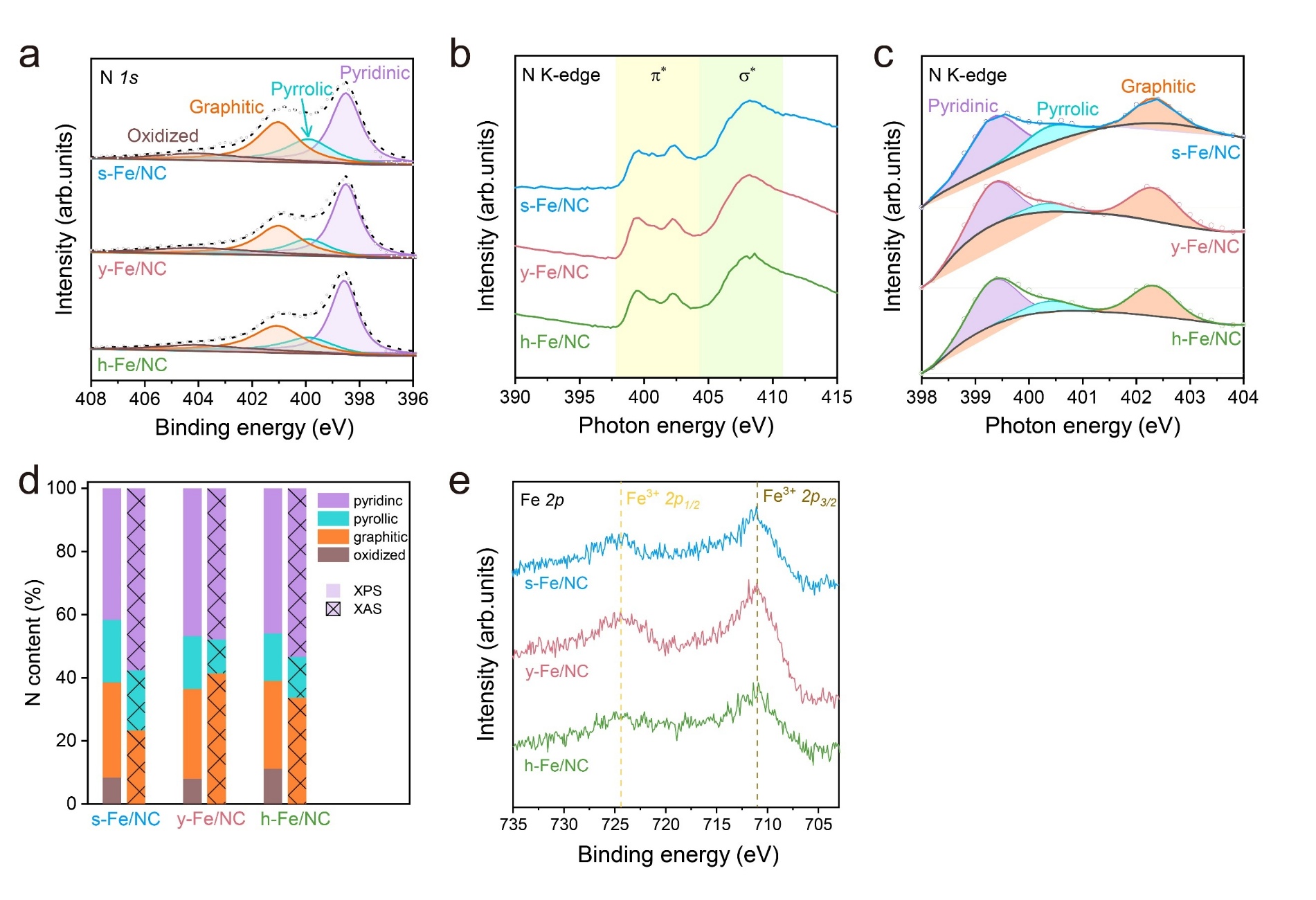
**

Fig. S15. N species analysis determined by XPS.

(a) High-resolution N *1s* XPS spectra. The high-resolution N *1s* XPS spectra of the obtained samples were deconvoluted into pyridinic (398.5 eV), pyrrolic (399.9 eV), graphitic (401.0 eV), and oxidized (404.1 eV) N species. (b) N K-edge XANES spectra, and (c) corresponding magnified and convoluted *π** regions of s-Fe/NC, y-Fe/NC, and h-Fe/NC. (d) Comparison of N percentage (%) in three SACs between XPS and XANES analysis. (e) High-resolution Fe *2p* XPS spectra.


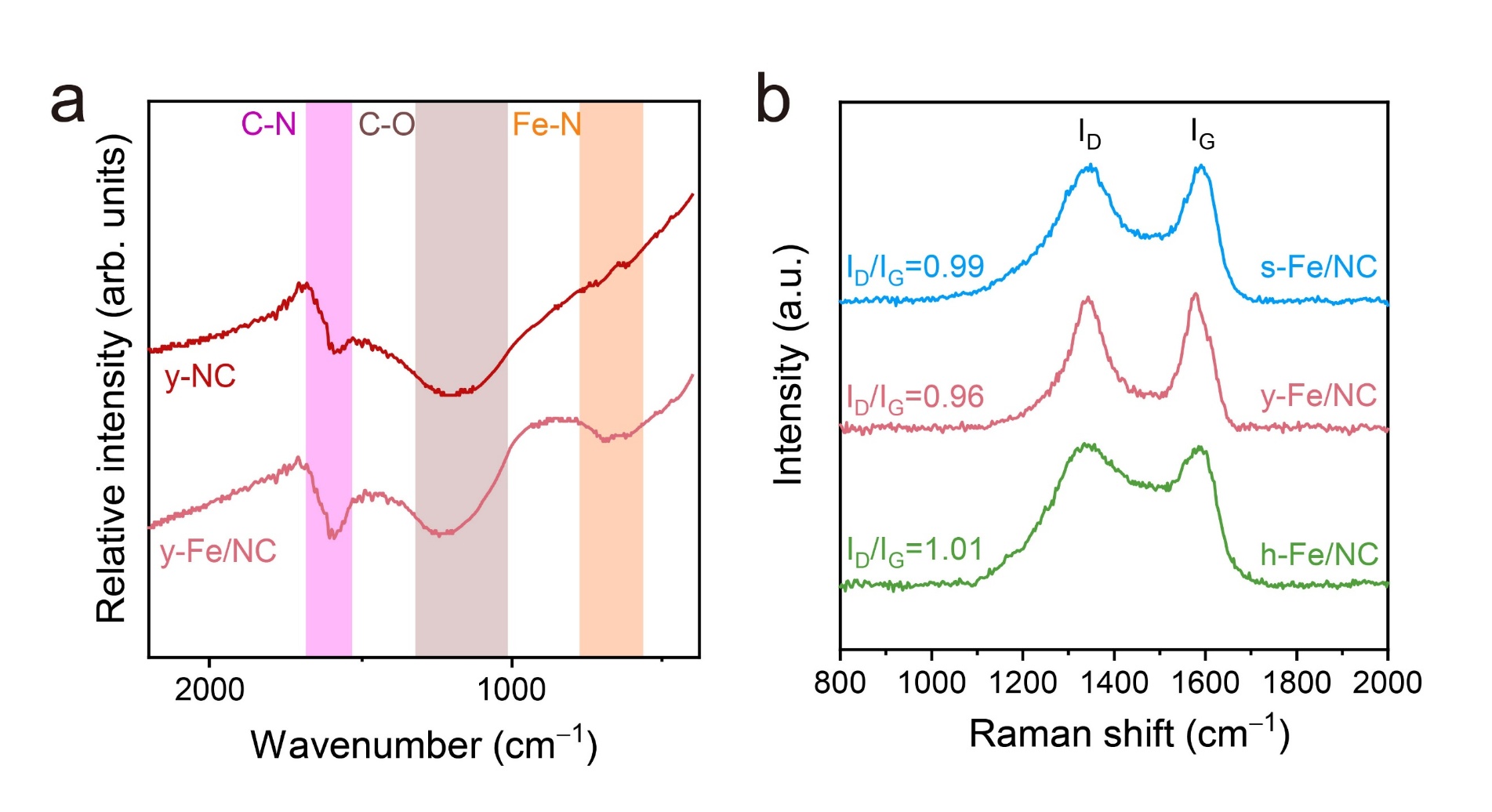


Fig. S16. FT-IR and Raman analysis.

1. FT-IR spectra of y-NC and y-Fe/NC. (b) Raman spectra of s-Fe/NC, y-Fe/NC, and h-Fe/NC.


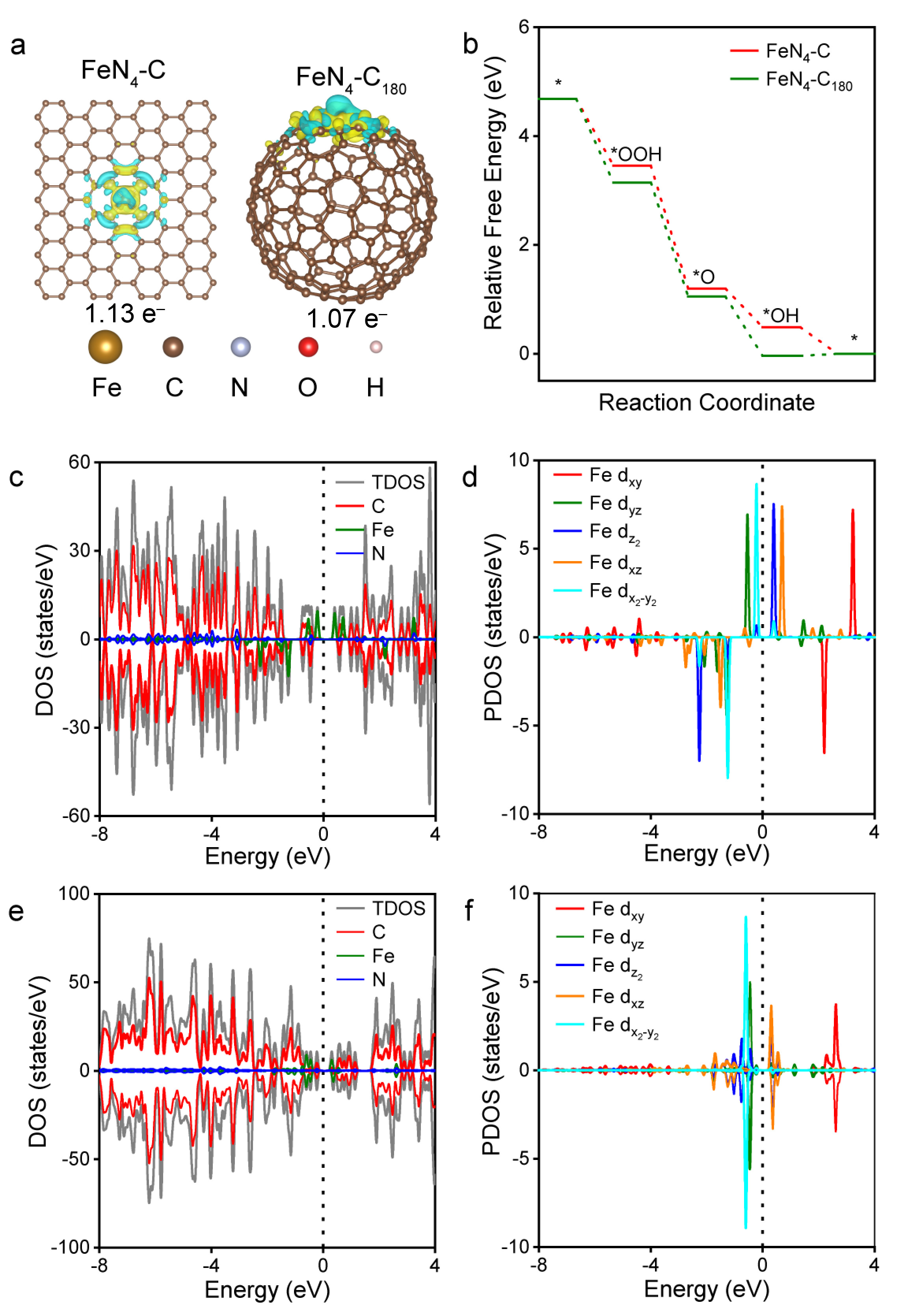


Fig. S17. DFT simulations.

(a) Charge density difference diagrams of FeN_4_-C and FeN_4_-C_180_. (b) Free energy paths of ORR at pH = 13 and T = 298 K. (c, e) DOS and (d, f) PDOS of (c, d) FeN_4_-C and (e, f) FeN_4_-C_180_, respectively.

**
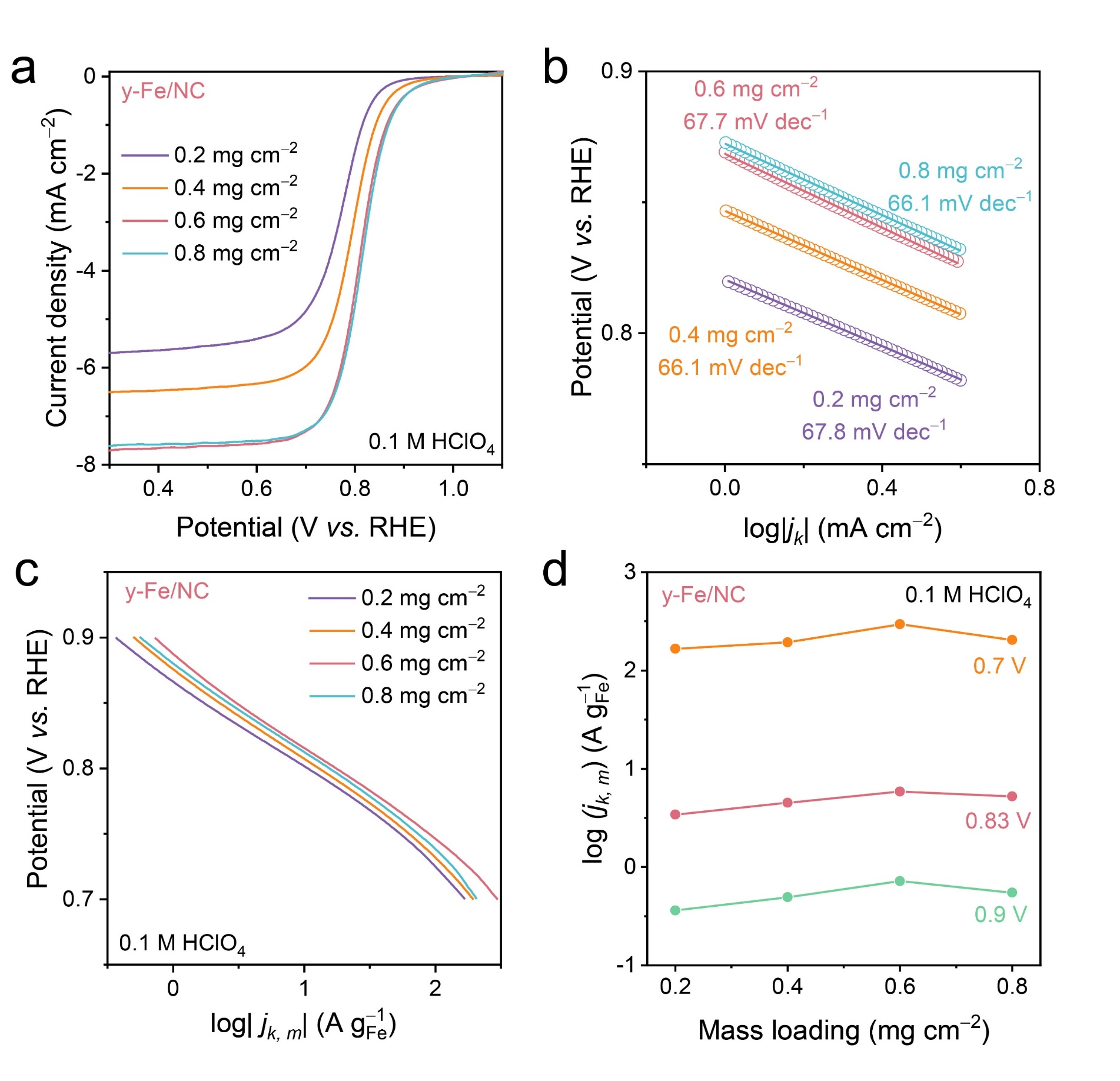
**

Fig. S18. ORR activity of y-Fe/NC with different loading.

(a) ORR polarization curves, (b) corresponding Tafel analysis and (c) mass ORR activities for y-Fe/NC with catalyst loading amounts of 0.2, 0.4, 0.6, and 0.8 mg cm^−2^, respectively. (d) Comparison of mass ORR activities at potential of 0.7 V (diffusion-controlled region), 0.83 V (mixed-controlled region), and 0.9 V (kinetic-controlled region) *vs.* RHE.

Note S2. Catalyst loading discussion on the RDE system.

The RDE theory based on the diffusion-convection kinetics of the pure solution laminar flow exhibits great advantages in eliminating mass transport issue^[17]^. However, a rational catalyst loading is critical to realize precise ORR performance evaluation. If the catalyst loading is too low, there is no uniform and full film formed in RDE. On the contrary, too high catalyst loading will introduce an extremely thick film, suppressing the O_2_ mass transportation to the interior layer^[18]^. Here, the rotational drying procedures were employed to get reproducible and uniform catalyst thin films^[19]^. As shown in Fig. S17, the mass activity of y-Fe/NC with the mass loading of 0.6 mg cm^−2^ reaches the highest under all regions, indicating the catalyst was completely utilized^[20]^. Thus, we select this loading amount for further performance comparison. Also, this loading amount is widely used in previous works, including *Nat. Energy* **2022**, 7, 652; *Adv. Mater.* **2023**, 35, 2300907; *Sci. Adv.* **2022**, 8, eadd8873.


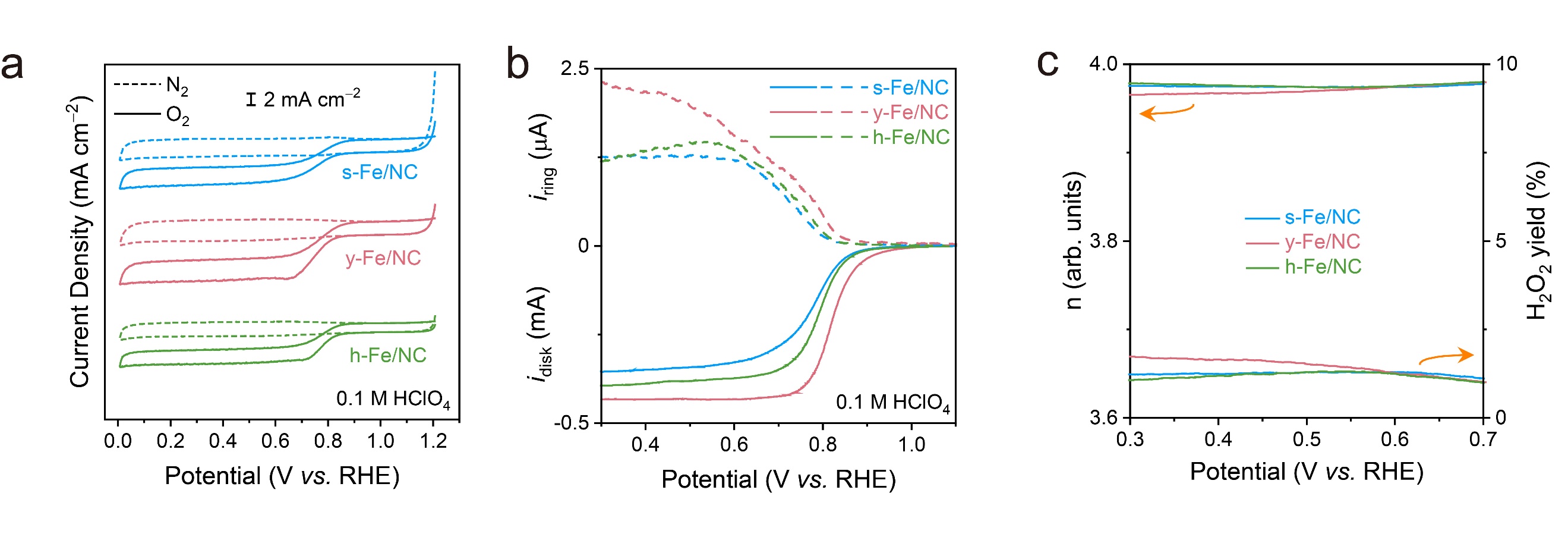


Fig. S19. RRDE measurements.

(a) CV curves for s-Fe/NC, y-Fe/NC, and h-Fe/NC under N_2_ (dashed lines) and O_2_ (solid lines). (b) ORR polarization curves of s-Fe/NC, y-Fe/NC, and h-Fe/NC (solid lines) together with the corresponding H_2_O_2_ currents on the ring electrode (dashed lines) recorded in O_2_-saturated 0.1 M HClO_4_ on a RRDE at 1600 rpm. (c) Calculated electron transfer number n and H_2_O_2_ yield during the potential sweep.

**
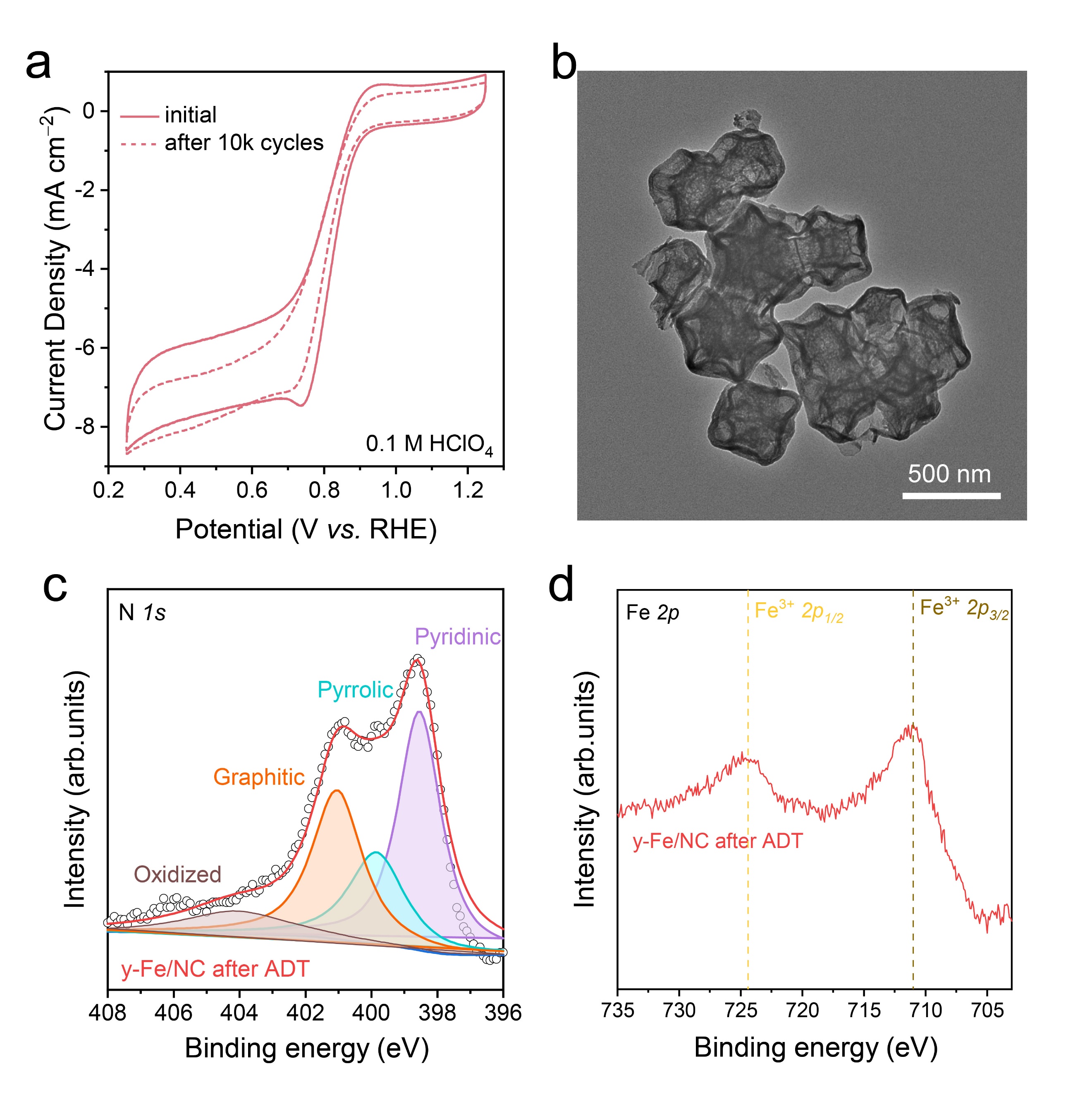
**

Fig. S20. Characterizations after accelerated degradation test.

(a) CV curves of y-Fe/NC before and after 10k cycles. (b) TEM image, (c) high-resolution N *1s* and (d) Fe *2p* XPS spectra of y-Fe/NC after 10k cycles of accelerated degradation test (ADT).

Note S3. Koutecky-Levich equation.

The electron transfer number (*n*) and kinetic current density (*j_k_*) can be calculated from the Koutecky-Levich equation (K-L)^[21]^:

$$\begin{aligned} \frac{1}{j}=\frac{1}{j_{d}}+\frac{1}{j_{k}}\# SEQ EQ 19 \end{aligned}$$

Where *j* is the measured current density, and *j_d_* is the diffusion-limiting current density. For all calculations of *j_k_* in this work, *j_d_* is defined as the current density at 0.4 V vs. RHE in the ORR polarization curves.

$$\begin{aligned} j_{td}=B\omega^{\frac{1}{2}}\# SEQ EQ 20 \end{aligned}$$

$$\begin{aligned} B=0.62nFC_{0}D_{0}^{\frac{2}{3}}V^{-\frac{1}{6}}\# SEQ EQ 21 \end{aligned}$$

Where *j_td_* is the theoretical diffusion-limiting current density, *n* is the total number of electrons transferred during the electrochemical reaction, *F* is the Faraday constant (96485 C mol^−1^); *C_0_* is the O_2_ saturated concentration (1.26×10^−6^ mol cm^−3^); *D_0_* is the O_2_ diffusion coefficient (1.93×10^−5^ cm^2^ s^−1^); *V* is the kinetic viscosity of solution (0.01 cm^2^ s^−1^), and *ω* is the rotation rate of the electrode. For 4e^−^ ORR, *j_td_* at 1600 rpm is a fixed value of 6.05 mA cm^−2^ in 0.1 M HClO_4_^[22]^.

Note S4. ORR polarization mathematical model.

The mathematical model of ORR polarization is established considering two limiting situations, i.e., the kinetic-controlled region and diffusion-controlled region. The relationship between overpotential and current density under the kinetic-controlled region with slow surface reaction kinetics and infinite fast mass transport was described by the Butler-Volmer (B-V) equation:

$$\begin{aligned} j_{k}=j_{0}\left[ -\exp\left( \frac{-\alpha nF}{RT}\eta\right)+\exp\left( \frac{\left( 1-\alpha\right)nF}{RT}\eta\right) \right]\# SEQ EQ 22 \end{aligned}$$

The relationship between mass transport and apparent electrochemical reaction rate under the diffusion-controlled region with fast surface reaction kinetics and sluggish mass transport was described by the Levich (L) equation:

$$\begin{aligned} j_{td}=\frac{i_{td}}{A_{real}}=\frac{i_{td}}{{RF\times A}_{geo}}=0.62nFC_{0}D_{0}^{\frac{2}{3}}{\omega^{\frac{1}{2}}V}^{-\frac{1}{6}}\# SEQ EQ 23 \end{aligned}$$

The electrode polarization curves follow the Nernstian reaction in Eq. 23:

$$\begin{aligned} \frac{j}{j_{0}}=\left( 1-\frac{j}{j_{td}} \right)\exp\left( -\alpha f\eta\right)-\left( 1-\frac{j}{j_{td}} \right)exp( \left( 1-\alpha)f\eta\right)\# SEQ EQ 24 \end{aligned}$$

Where *j* is the apparent current density, *j_td_* is the theoretical diffusion-limiting current density, A_real_ is the active surface area, A_geo_ is the geometric area, roughness factor (RF) is the ratio between A_real_ and A_geo_, *j_k_* is the kinetic current density, *j_0_* is the exchange current density estimated from the polarization curves (as indicated in Table S5), *α* is the exchange coefficient, *n* is the total number of electrons transferred during the electrochemical reaction, *F* is the Faraday constant (96485 C mol^−1^), *R* is the universal gas constant (8.314 J K^−1^ mol^−1^), *T* is the absolute room temperature (298 K), *f=F/RT*, and *η* is the overpotential, *C_0_* is the O_2_ saturated concentration (1.26×10^−6^ mol cm^−3^); *D_0_* is the O_2_ diffusion coefficient (1.93×10^−5^ cm^2^ s^−1^); *V* is the kinetic viscosity of solution (0.01 cm^2^ s^−1^), and *ω* is the rotation rate of the electrode (1600 rpm). Based on the above, the apparent ORR polarization curves can be described regarding coupled factors of surface reaction and mass transport effect (Fig. S20)^[23]^. Only four parameters (*j_0_*, *α*, *n*, and *A*) are variables, and only *n* and *A* show impacts on the experimental diffusion current density. Moreover, the experimental ORR polarization curves can be fitted using above mentioned mathematical model *via* Python-based least square method with the help of VSCode (Fig. S21). The current density is selected as the explained variable, and the parameters include the *j_0_*, *α*, *n*, and *A* as variables to be fitted. The fitting potential range is chosen as 0.3 to 1.0 V vs. RHE to avoid the possible hydrogen underpotential deposition^[24]^.


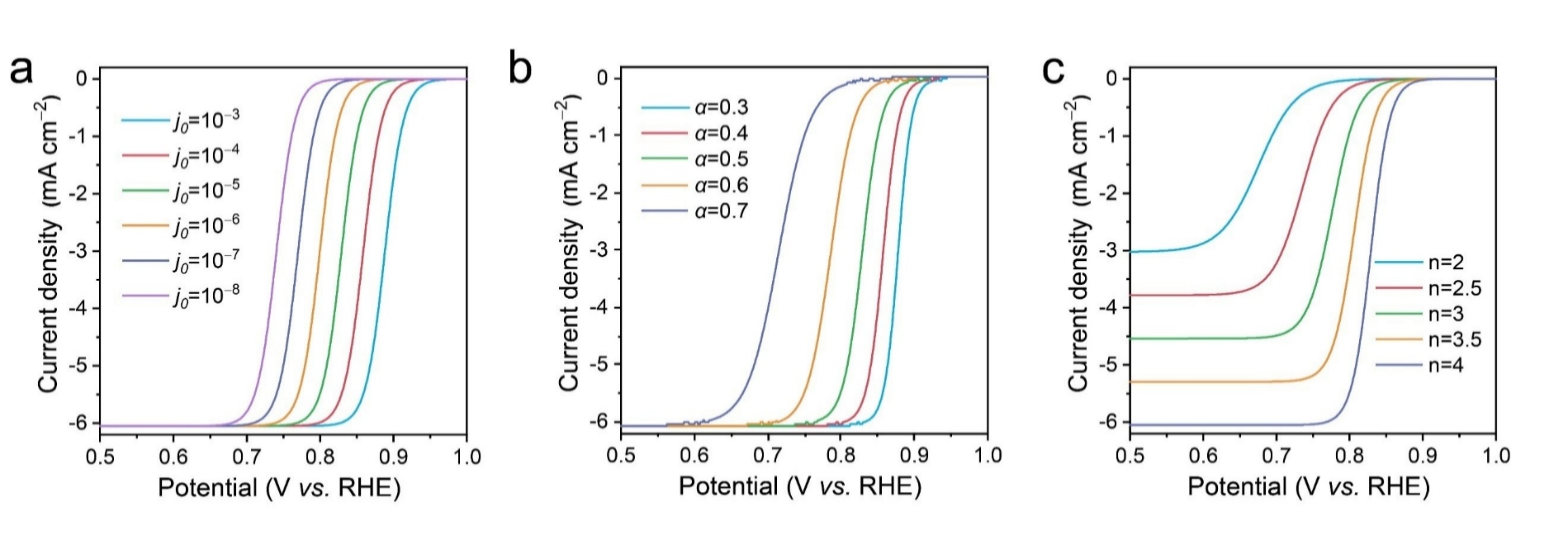


Fig. S21. ORR mathematical model.

Simulated ORR polarization curves with different (a) exchange current density from 10^−8^ to 10^−3^ mA cm^−2^, (b) exchange coefficient from 0.3 to 0.7, and (c) electron transfer number from 2 to 4. It is worth noting that the default parameters are 10^−5^ mA cm^−2^ for *j_0_*, 0.5 for *α*, and 4 for *n*, respectively.


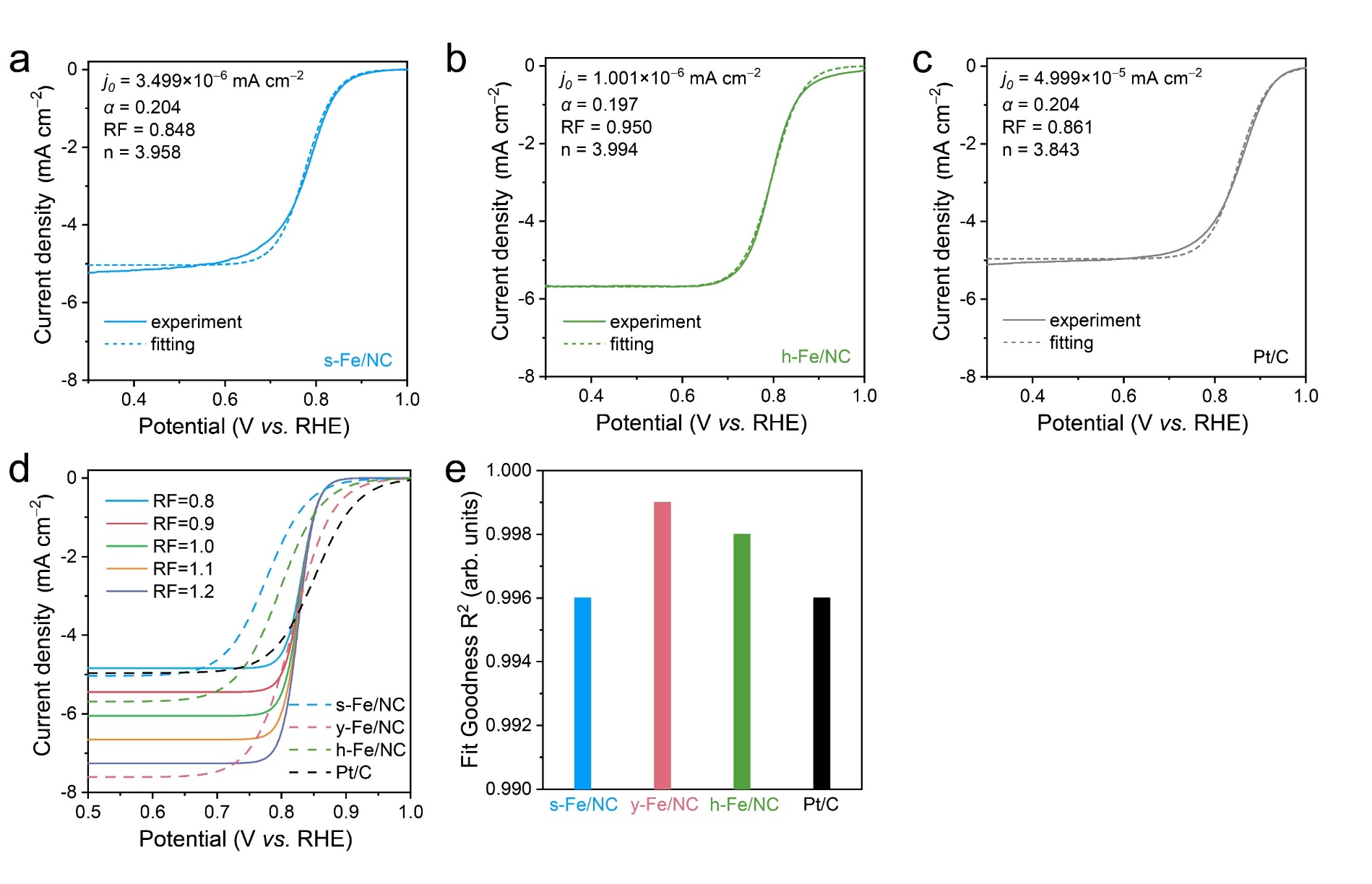


Fig. S22. ORR polarization curve fitting.

Experimental and fitting ORR polarization curves for (a) s-Fe/NC, (b) h-Fe/NC, and (c) Pt/C. (d) Simulated ORR polarization curves using different relations of RF. (e) Goodness of fit comparisons of obtained catalysts.

Note S5 Porous RDE model.

According to the Levich equation, the current *i* *vs.* the square root of the rotation rate yields a linear relationship in the mass-transport-limited region for a flat electrode. However, for a porous RDE model, the dependence of the current on angular velocity deviates from linearity^[25]^. As the rotation rate increases, the current increases more rapidly than predicted by the Levich equation, which is influenced by the thickness and porosity of the porous medium^[26]^. This phenomenon can be explained by the increased effective electrode surface area resulting from reactant perfusion induced by the local recirculation effect. The volumetric flow of the perfused reactant is calculated by:

$$\begin{aligned} Q=2k{\omega^{2}\pi R^{2}hV}^{-1}\# SEQ EQ 25 \end{aligned}$$

Where Q is the volumetric flow, *k* is the permeability of the porous medium, *ω* is the rotation rate of the electrode, R is the radius of the electrode, h is the thickness of the porous medium, and *V* is the kinetic viscosity of the solution (0.01 cm^2^ s^−1^). At a lower rotation rate, minimal reactants are infused into the porous medium, whereas at higher rates, the pores are saturated, resulting in an S-shaped relationship between the current and rotation rate in porous RDE. As shown in Fig. S22, the porous RDE are unsaturated below 2500 rpm. Thus, the porous RDE can be regarded as a combination of a flat RDE and an axial plug-flow reactor with complex flows, resulting in nonlinear and complicated dependence on rotating rate and current^[27]^. For completely perfused porous RDE, the flux of reactant to surface is approximately (D_0_C_0_)Q; thus, the estimated maximum current I_M_ is calculated by:

$$\begin{aligned} I_{M}=nFD_{0}C_{0}Q\pi r^{2}h\# SEQ EQ 26 \end{aligned}$$

where *Q* is the volumetric flow; *C_0_* is the O_2_ saturated concentration (1.26×10^−6^ mol cm^−3^); *D_0_* is the O_2_ diffusion coefficient (1.93×10^−5^ cm^2^ s^−1^); *n* is the total number of electrons transferred during the electrochemical reaction, *F* is the Faraday constant (96485 C mol^−1^), *πr^2^h* is the volume of the disk.


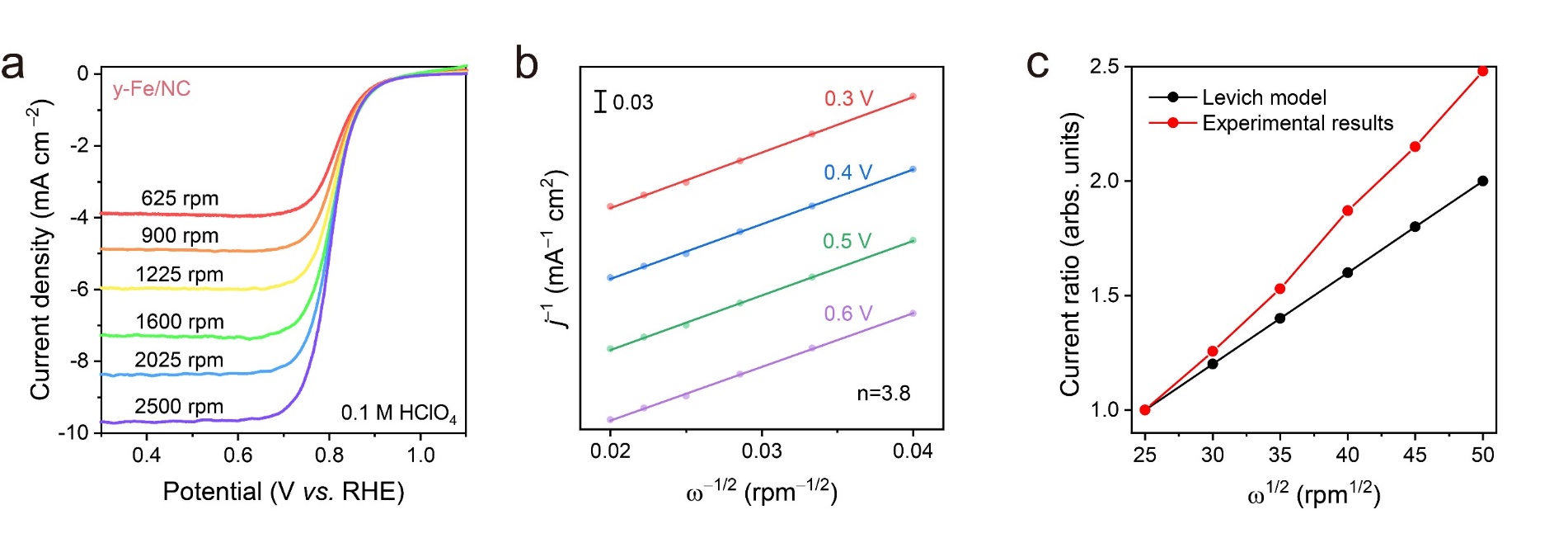


Fig. S23. K-L plots.

(a) ORR polarization curves of y-Fe/NC recorded in O_2_-saturated 0.1 M HClO_4_ on a RDE at different rotating speeds. (b) K-L plots of y-Fe/NC in the potential range from 0.3 V to 0.6 V *vs.* RHE. The electron transfer number n is measured as 3.8. (c) Relationship between current ratio *vs.* square root of the rotation rate, details can be found in Note S6. The experimental current ratio is calculated by dividing the current at a specific rate by that at the lowest rate.


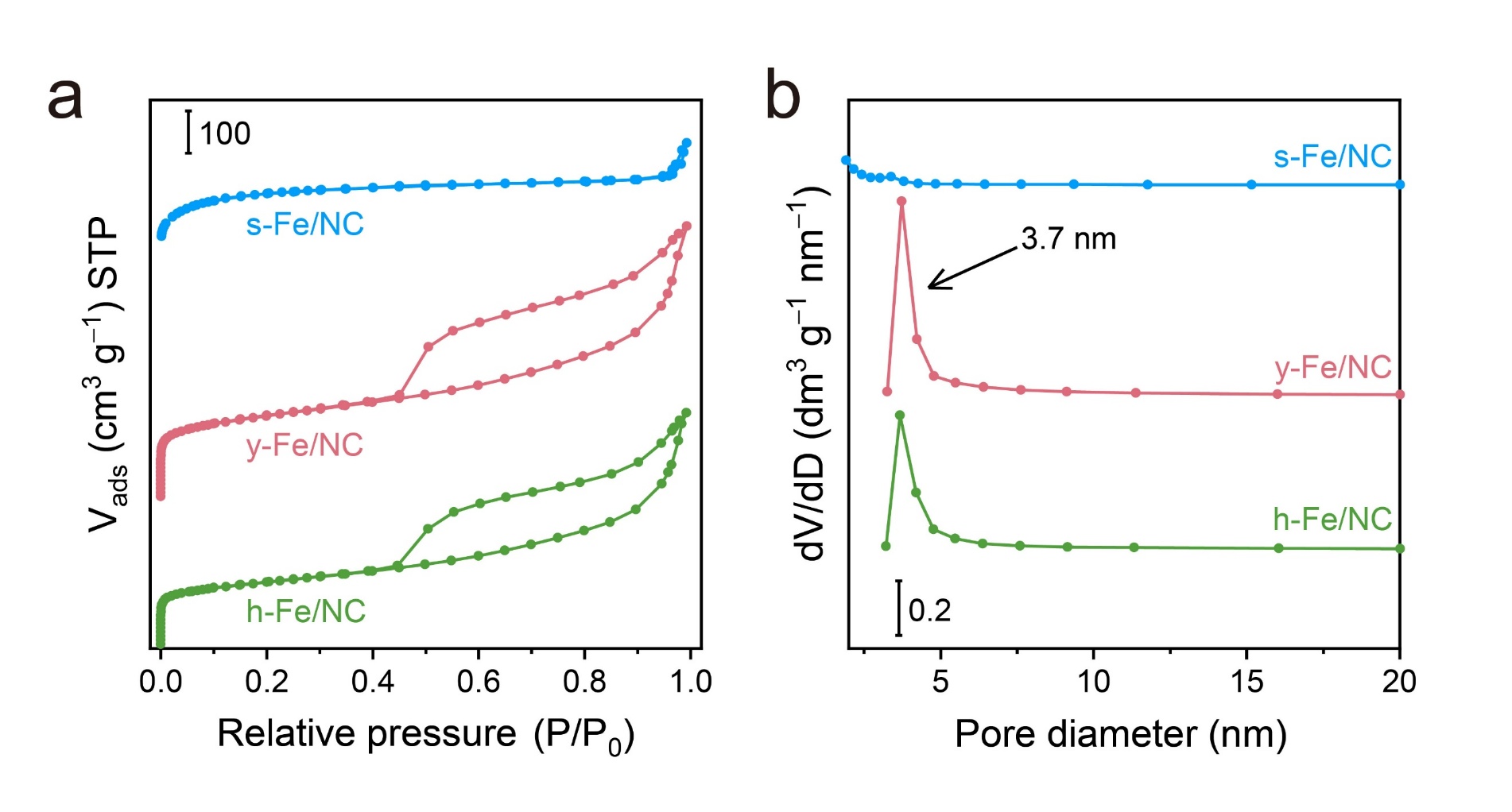


Fig. S24. Porosity analysis.

(a) N_2_ adsorption-desorption isotherms and (b) pore size distributions of s-Fe/NC, y-Fe/NC, and h-Fe/NC. Type IV hysteresis loops observed in y-Fe/NC and h-Fe/NC indicate the existence of micro/meso-pores within the carbon matrix and macropores forming the void. In contrast, only micro/meso-pores were observed in s-Fe/NC (Type II)^[28]^. The average diameter of mesopores was 3.7 nm in all Fe/NC samples.


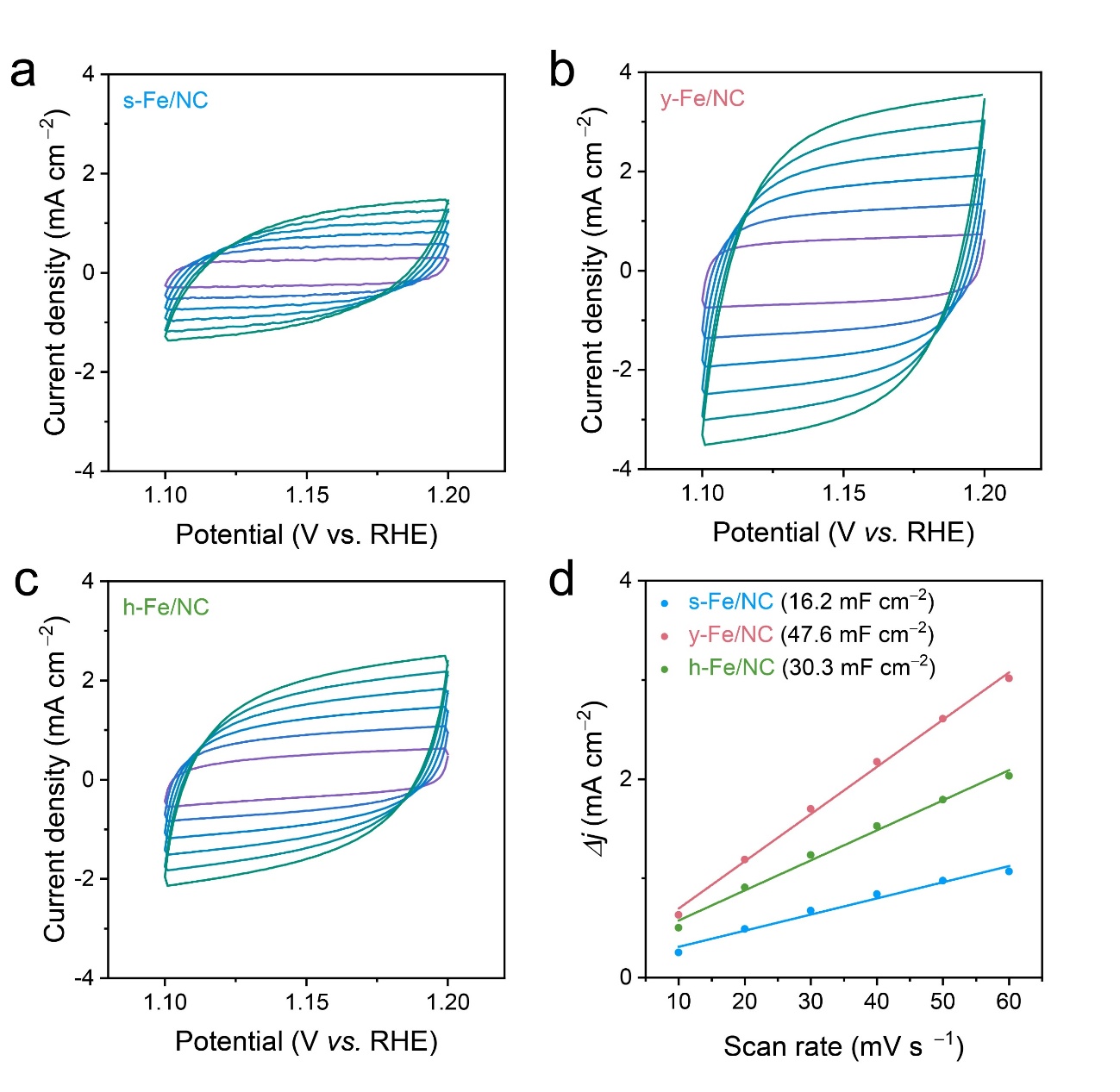


Fig. S25. Electrochemical surface area measurement.

Cyclic voltammetry curves of (a) s-Fe/NC, (b) y-Fe/NC, and (c) h-Fe/NC in N_2_-saturated 0.1 M HClO_4_ at scan rates from 10 to 60 mV s^−1^. (d) The corresponding electrochemical double-layer capacity of s-Fe/NC, y-Fe/NC, and h-Fe/NC determined by fitting plots of the current density at 1.1 V versus the scan rate. By using *C_s_* as 40 µF cm^−2^, the ECSA is calculated as 405, 1190, and 758 cm_ESCA_^2^ for s-Fe/NC, y-Fe/NC and h-Fe/NC, respectively.


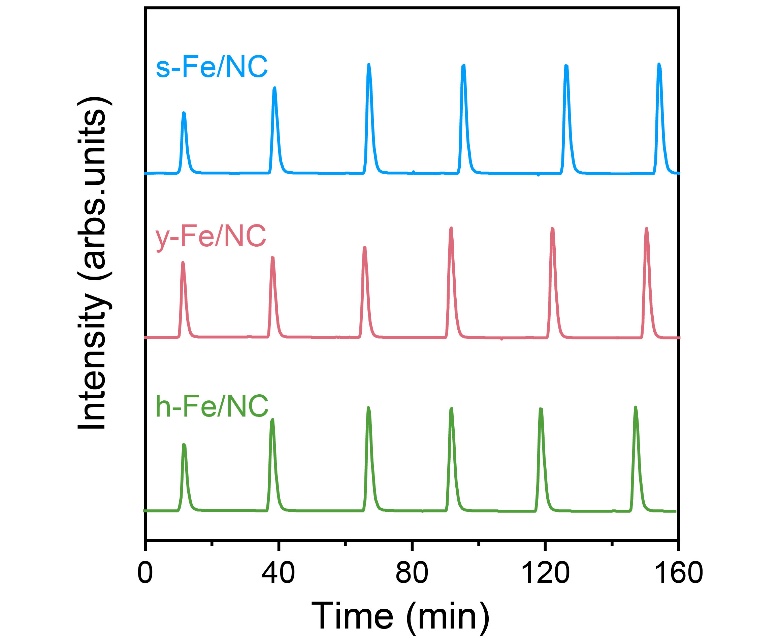


Fig. S26. Adsorption character to CO species.

CO pulse chemisorption profiles of s-Fe/NC, y-Fe/NC and h-Fe/NC, where the reduced pulse peak area indicates the CO uptake.


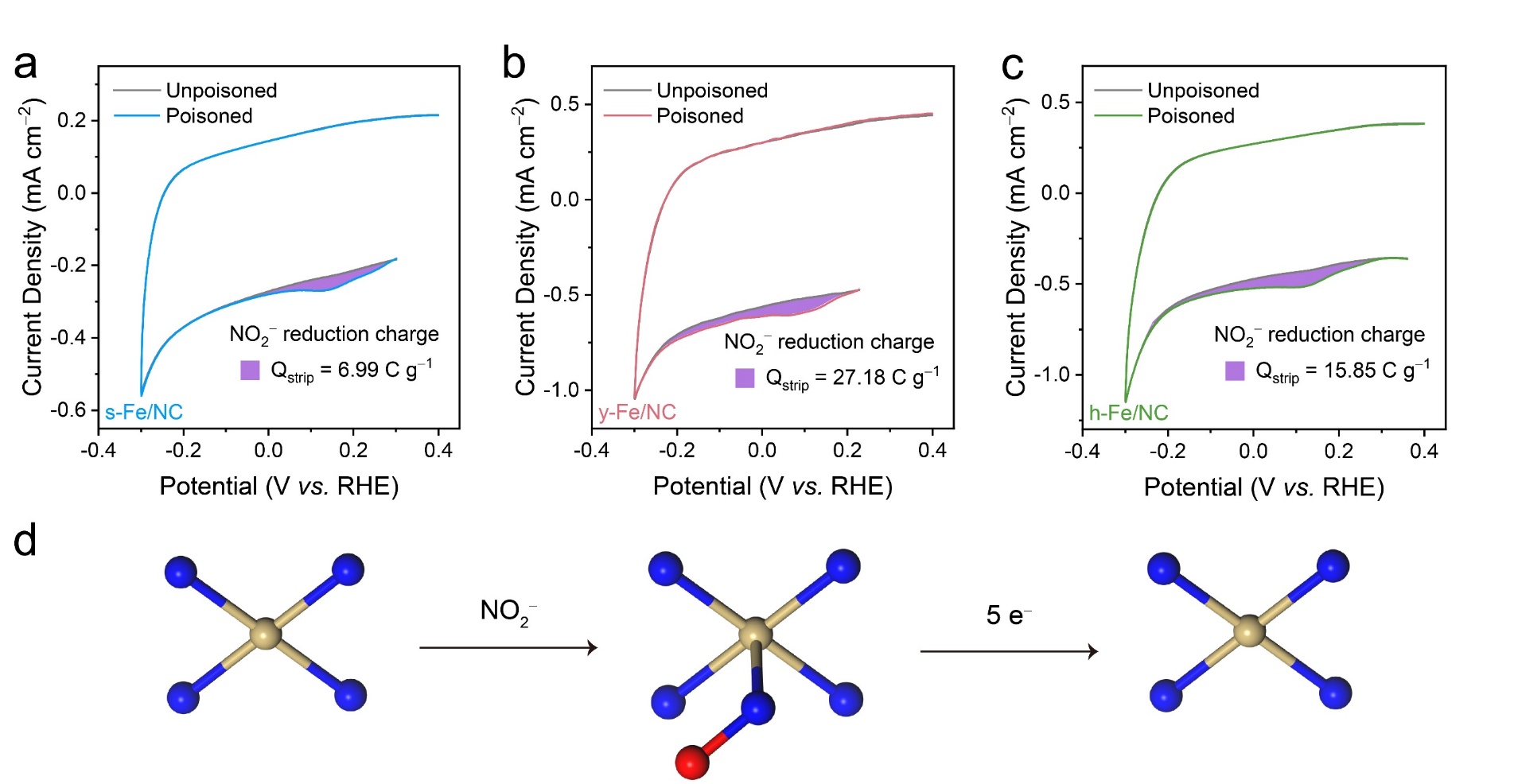


Fig. S27. Nitrite stripping voltammetry of Fe/NCs.

CV curves of s-Fe/NC (a), y-Fe/NC (b), and h-Fe/NC (c) before and during nitrite adsorption in the nitrite reductive stripping region in an N_2_-saturated 0.5 M acetate buffer at pH 5.2, respectively. (d) Schematic illustration of the method principle.

Note S6. Turnover frequency calculation.

The turnover frequency (TOF) quantifies the chemical conversions of reactant molecules per active site and unit time, representing a pivotal parameter for delineating the intrinsic activity of the catalytic site. For ORR, TOF is estimated by the combined input of the kinetic current density of a catalyst and its active MSD. In this work, three types of electrode area were employed to normalize the kinetic current density, including geometric (geo) area, BET area, and C_dl_-based ECSA. Moreover, three types of MSD were determined using ICP, CO chemisorption, and nitrite stripping (NS), respectively. Thus, TOFs can be calculated based on the following equation:

$$\begin{aligned} {TOF}_{x,y}\left[ e^{-}{site}^{-1} s^{-1} \right]=\frac{i_{k}\times N_{A}}{A_{x}\times{MSD}_{y}\times F\times m_{cat}}\# SEQ EQ 27 \end{aligned}$$

Where *i_k_* is the kinetic current, A*_x_* is the surface area based on *x* strategy, *x* presents geo, BET, and ECSA; M*SD_y_* is the mass site density based on *y* strategy, *y* presents ICP, CO, and NS, *F* is the faradaic constant, *N_A_* is the Avogadro`s constant, and *m_cat_* is the mass of catalyst. As shown in Fig. S27, the resulting TOF values can be labeled as TOF_geo-ICP_, TOF_geo-CO_, TOF_geo-NS_, TOF_BET-ICP_, TOF_BET-CO_, TOF_BET-NS_, TOF_ECSA-ICP_, TOF_ECSA-CO_, and TOF_ECSA-NS_, respectively.

The calculation of standard deviation (SD) among three samples in Fig. 2e are listed as following:

1. For TOF values at 0.8 V *vs.* RHE normalized by each method for three Fe/NC catalysts, we have one value (x_1_, x_2_, and x_3_).

2. The mean across three samples can be calculated as x̅.

3. The SD can be calculated using

$$\begin{aligned} s=\sqrt{\frac{{(x_{1}-\bar{x})}^{2}+{(x_{2}-\bar{x})}^{2}+{(x_{3}-\bar{x})}^{2}}{3-1}}\# SEQ EQ 28 \end{aligned}$$

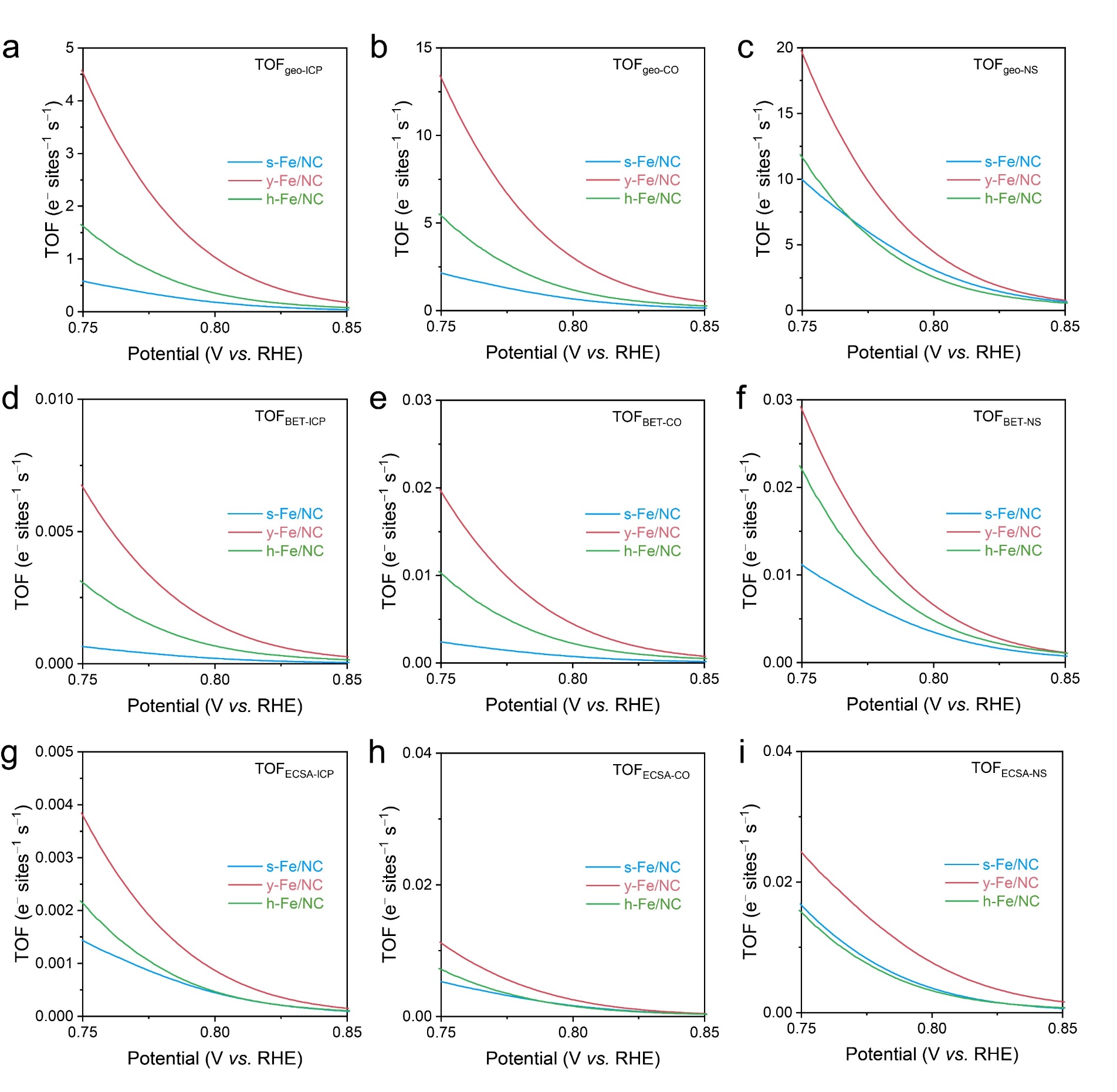


Fig. S28. Turnover frequency calculation.

The calculated (a) TOF_geo-ICP_, (b) TOF_geo-CO_, (c) TOF_geo-NS_, (d) TOF_BET-ICP_, (e) TOF_BET-CO_, (f) TOF_BET-NS_, (g) TOF_ECSA-ICP_, (h) TOF_ECSA-CO_, and (i) TOF_ECSA-NS_ based on Fig. 2a.


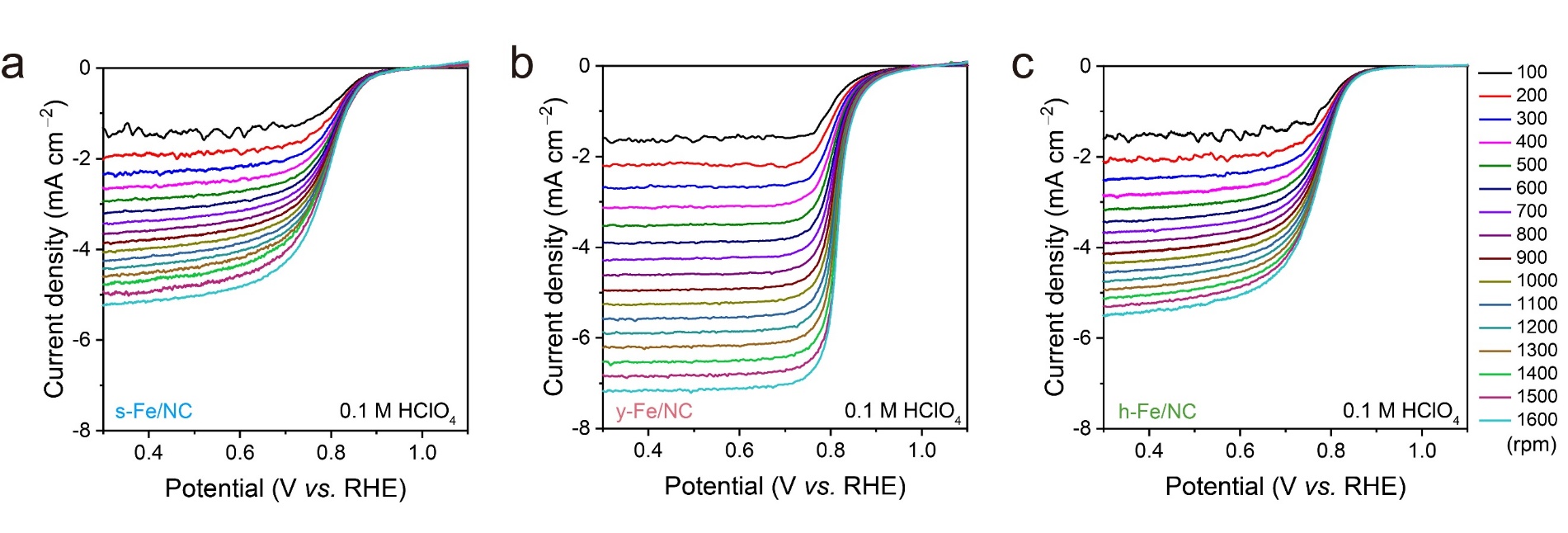


Fig. S29. Rotating speed exploration.

ORR polarization curves of (a) s-Fe/NC, (b) y-Fe/NC, and (c) h-Fe/NC recorded in O_2_-saturated 0.1 M HClO_4_ on a RDE at different rotating speeds from 100 to 1600 rpm.


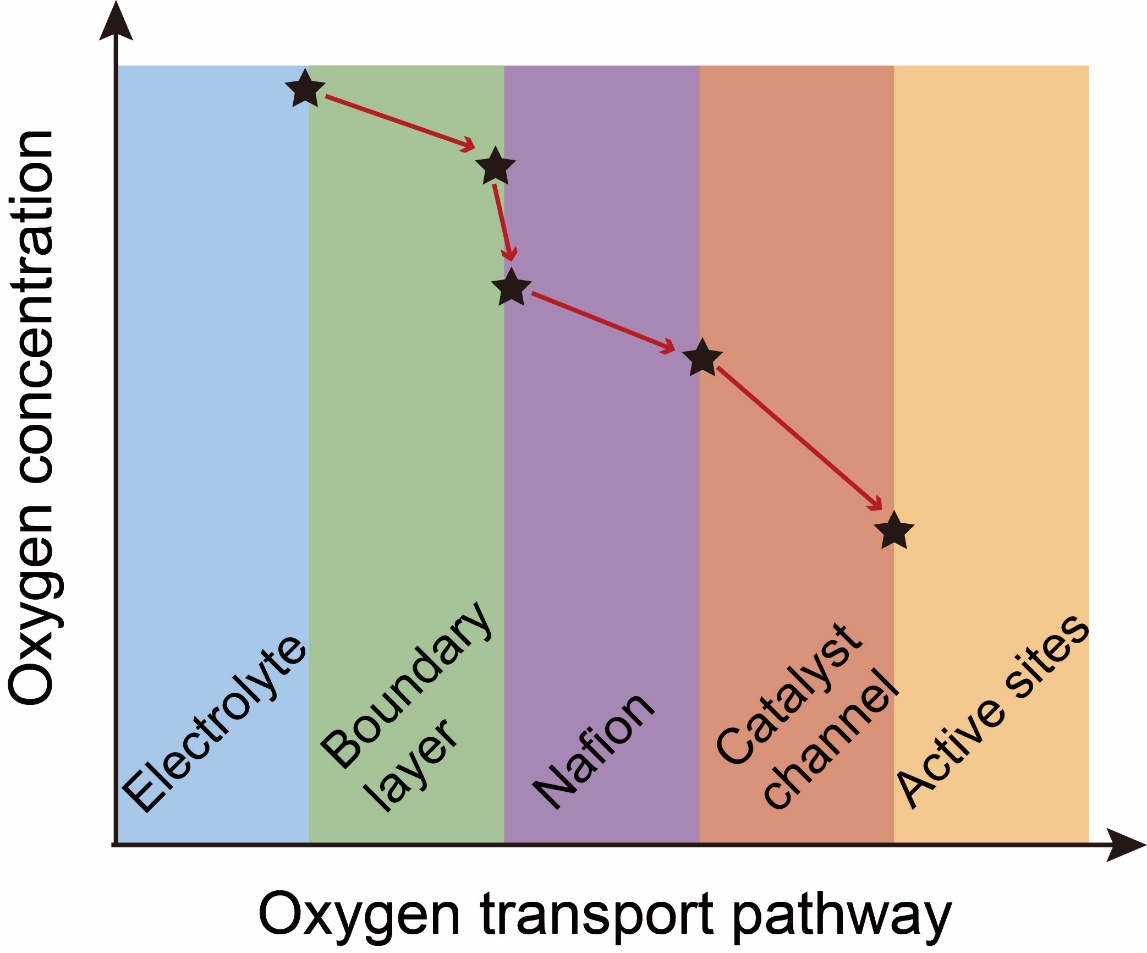


Fig. S30. Oxygen mass transport pathway.

Schematic diagram of the oxygen transport pathway from the electrolyte to the active sites.

Note S7. Oxygen transfer resistance calculation.

If the oxygen reduction reaction occurred on the ideal smooth surface, without involving the attributes of electrode structure, the theoretical total oxygen transfer resistance (*TR_O2_*, unit: s m^−1^) for oxygen transferred from the electrolyte to the active site can be calculated by the following equation^[29]^:

$$\begin{aligned} {TR}_{O_{2}}= \frac{C_{O_{2}}\times n\times F}{B\omega^{\frac{1}{2}}}\# SEQ EQ 29 \end{aligned}$$

The experimental total oxygen transfer resistances (*ER_O2_*, unit: s m^−1^) can be indirectly calculated by the following equation:

$$\begin{aligned} {ER}_{O_{2}}= \frac{C_{O_{2}}\times n\times F}{j_{d}}\# SEQ EQ 30 \end{aligned}$$

Where *j_d_* is the experimental diffusion-limiting current density (mA cm^−2^), *n* is the total number of electrons transferred during the electrochemical reaction, *F* is the Faraday constant (96485 C mol^−1^); *C_O2_* is the O_2_ saturated concentration (1.26×10^−6^ mol cm^−3^); *ω* is the rotation rate of the electrode.

By considering the boundary condition of a real electrode, Eq. 28 can be rewritten in a new form^[30]^:

$$\begin{aligned} {ER}_{O_{2}}=A_{O_{2}}+TR_{O_{2}} \# SEQ EQ 31 \end{aligned}$$

Where *A_O2_* is a correction parameter of *ER_O2_* as *ω* approaches infinity, and is dependent on the electrode structure only.

We assumed that the *R_O2_* can be separated into the transport resistance in the electrolyte, boundary layer, Nafion, catalyst channel, and active sites, respectively. In this work, only the catalyst channel due to the structure design is considered as a variable, and its dependence on various structures of Fe/NC-based nanoreactors is analyzed.


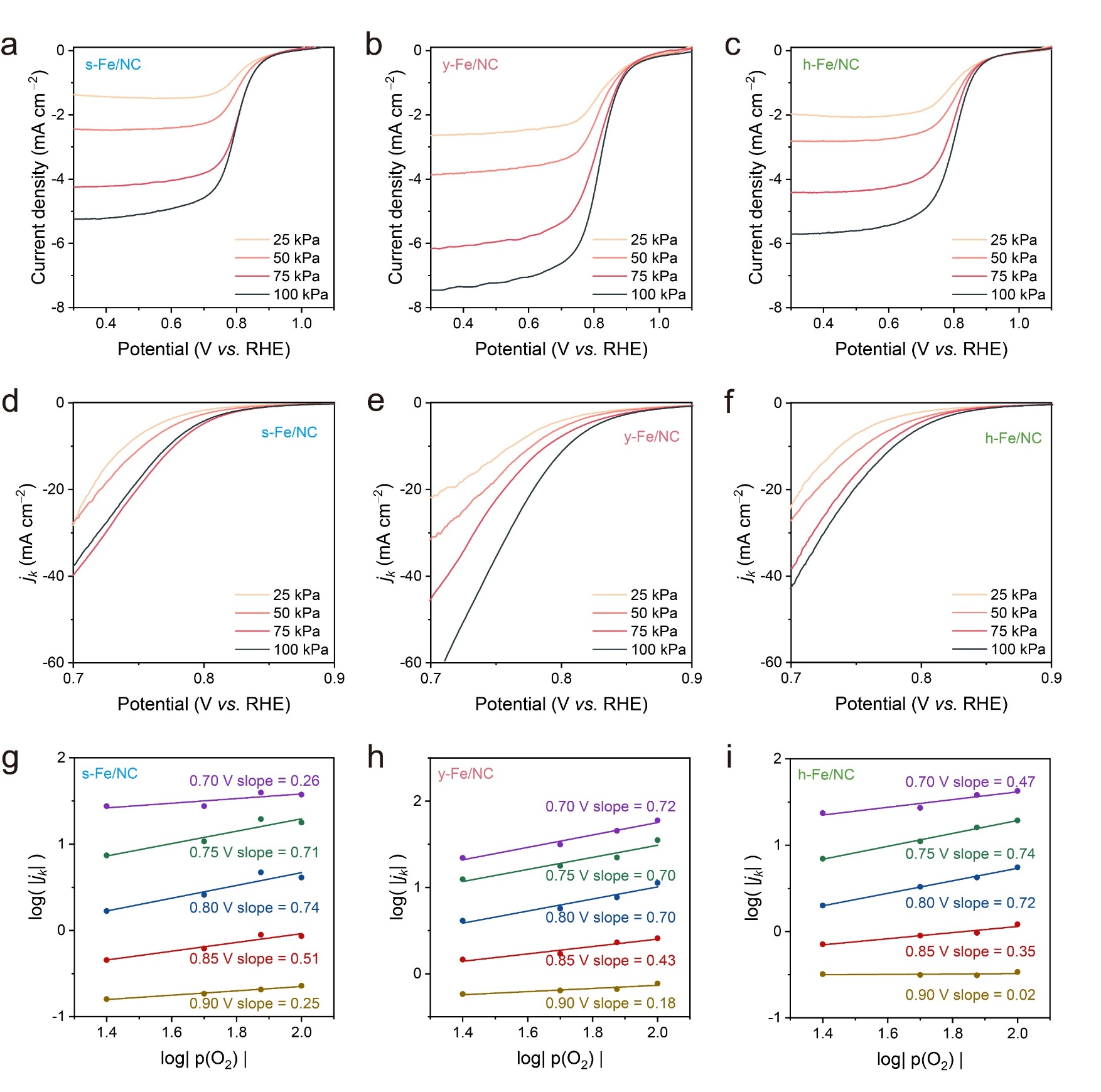


Fig. S31. Oxygen partial pressure regulation.

(a-c) ORR polarization curves, (d-f) the corresponding kinetic current density *versus* potential curves, and (g-i) reaction orders of O_2_ at different potentials of s-Fe/NC (a, d, g), y-Fe/NC (b, e, h), and h-Fe/NC (c, f, i) recorded in O_2_-saturated 0.1 M HClO_4_ on a RDE at different oxygen partial pressure.

Note S8. Oxygen partial pressure exploration.

According to Henry’s law, the O_2_ partial pressure in electrolyte is regulated by controlling the oxygen percentage of the gas inlet through the N_2_ diluting strategy^[31]^. For example, an O_2_ partial pressure of 25 kPa was obtained by mixing gas of O_2_ (50 mL min^−1^) and N_2_ (150 mL min^−1^), while maintaining the total gas flow rate as 200 mL min^−1^. All Fe/NC nanoreactors were performed under 25, 50, 75, and 100 kPa, respectively. The logarithm of the kinetic current density of samples versus the logarithm of the O_2_ partial pressure was plotted in Fig. S30g-i, and the reaction order of O_2_ can be determined as the slope of the fitting line.


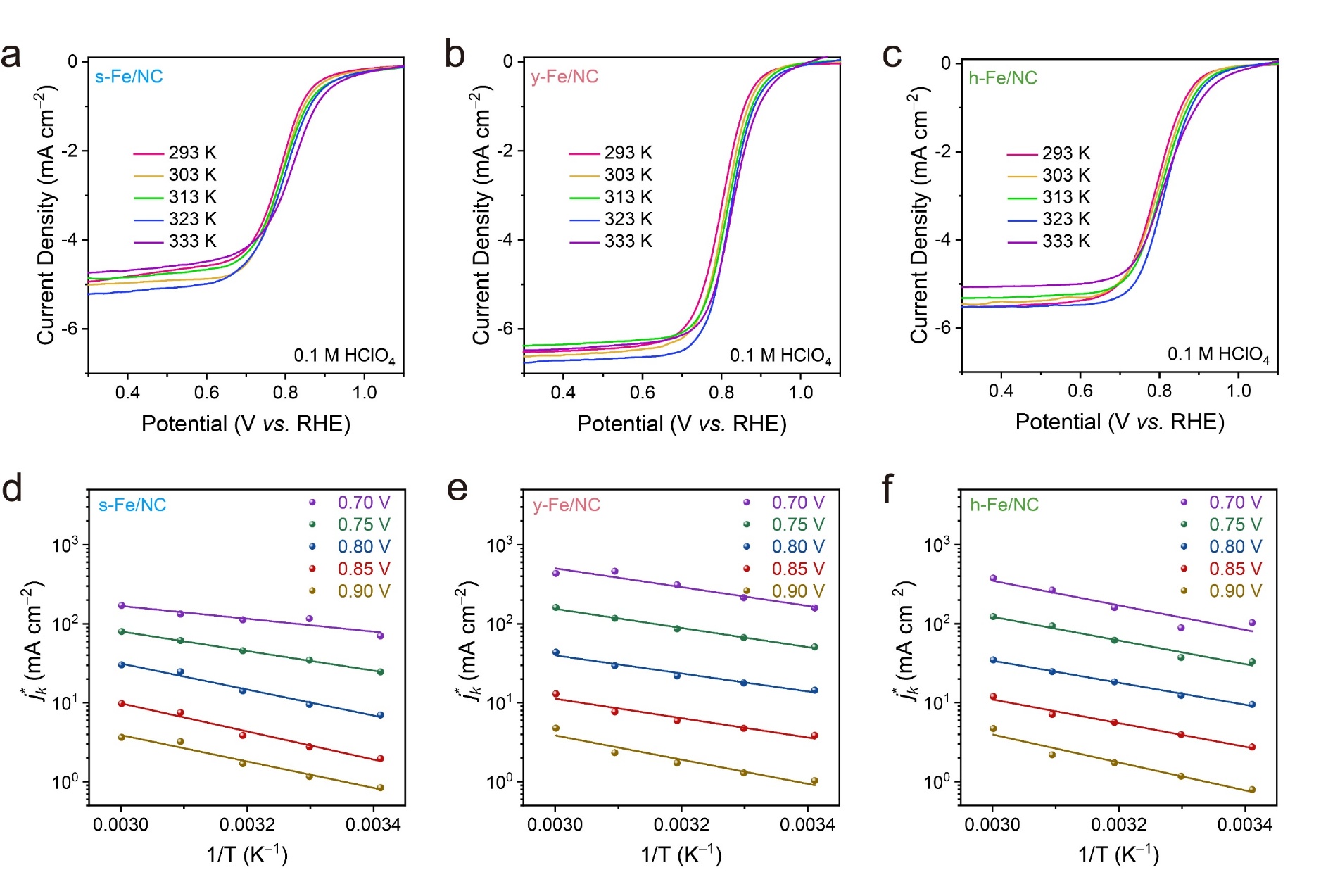


Fig. S32. Reaction temperature exploration.

(a-c) ORR polarization curves and (d-f) Arrhenius plots at different potentials of s-Fe/NC (a, d), y-Fe/NC (b,e), and h-Fe/NC (c,f) recorded in O_2_-saturated 0.1 M HClO_4_ on a RDE at different temperatures.


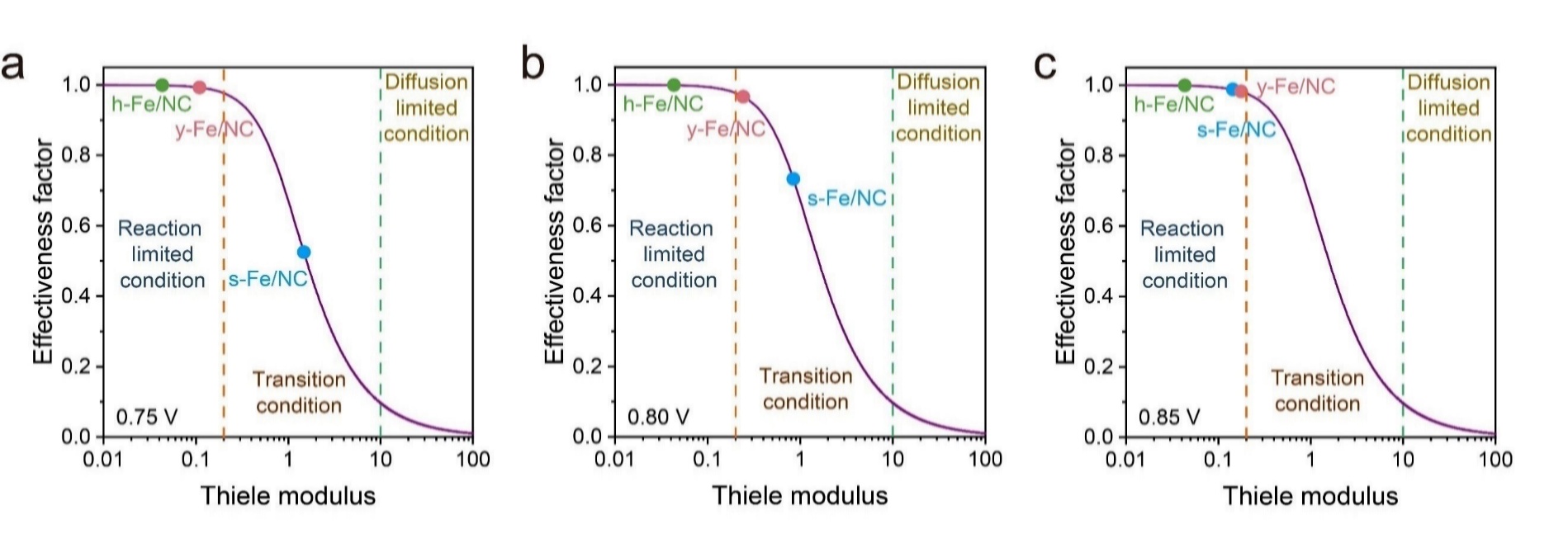


Fig. S33. Thiele modulus and effectiveness factor.

Thiele modulus and effectiveness factor of all Fe/NC-based nanoreactors at (a) 0.75 V, (b) 0.8 V and (c) 0.85 V, respectively.


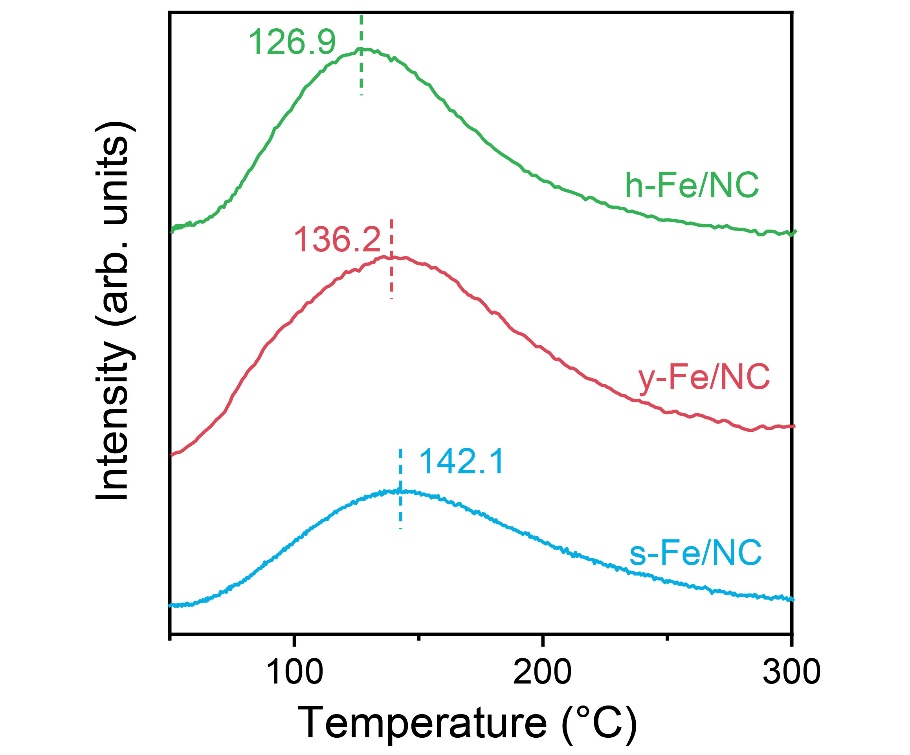


Fig. S34. Adsorption character to O_2_ species.

O_2_-TPD profiles of s-Fe/NC, y-Fe/NC and h-Fe/NC.


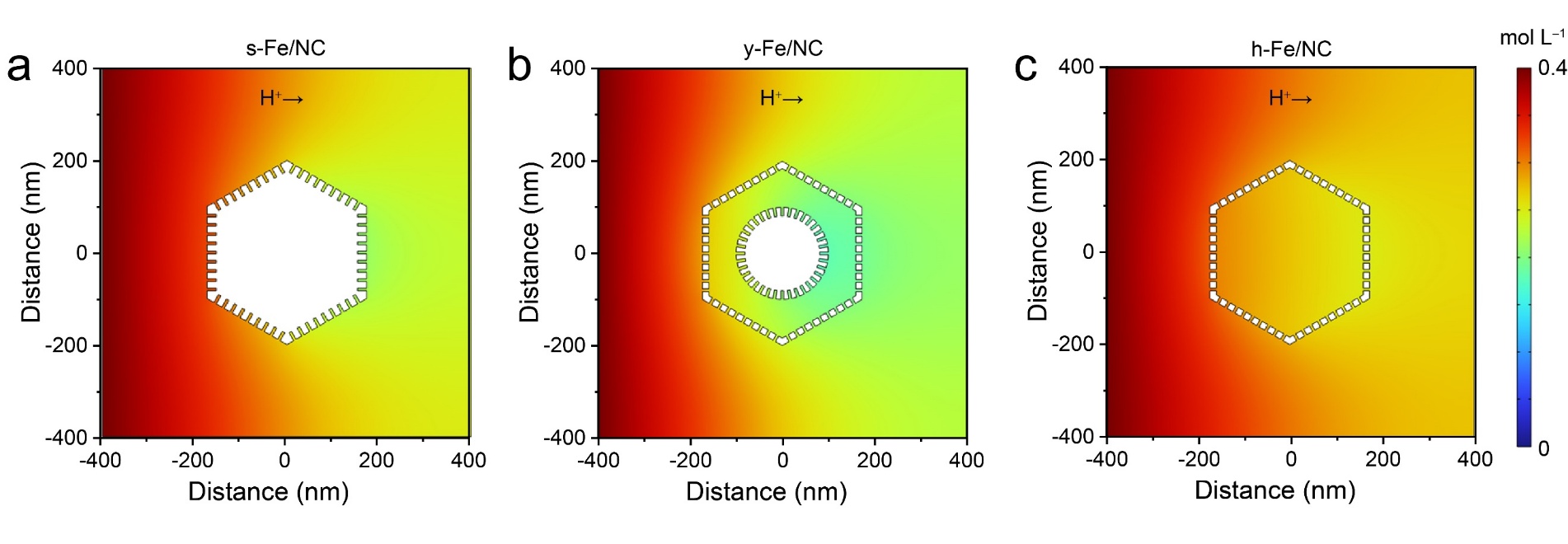


Fig. S35. Local H^+^ concentration by FEM simulations.

Distribution of the proton concentration around the single model (a) s-Fe/NC, (b) y-Fe/NC, and (c) h-Fe/NC.


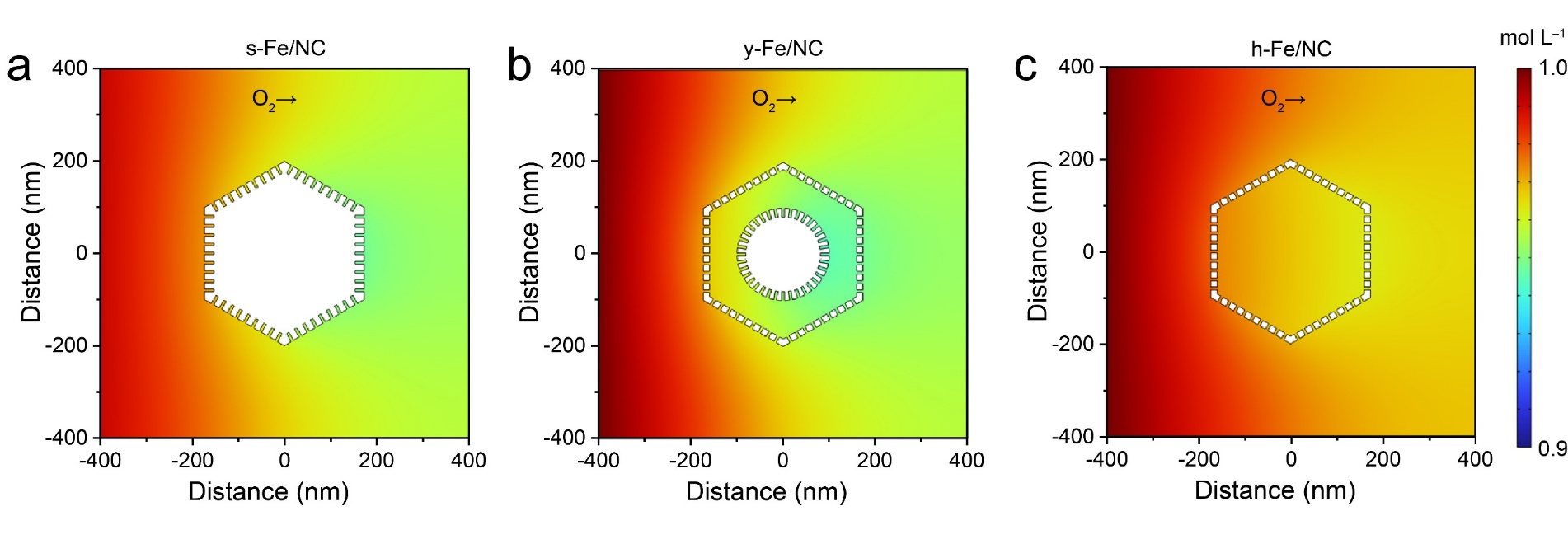


Fig. S36. Local O_2_ concentration by FEM simulations.

Distribution of the oxygen concentration around the single model (a) s-Fe/NC, (b) y-Fe/NC, and (c) h-Fe/NC.


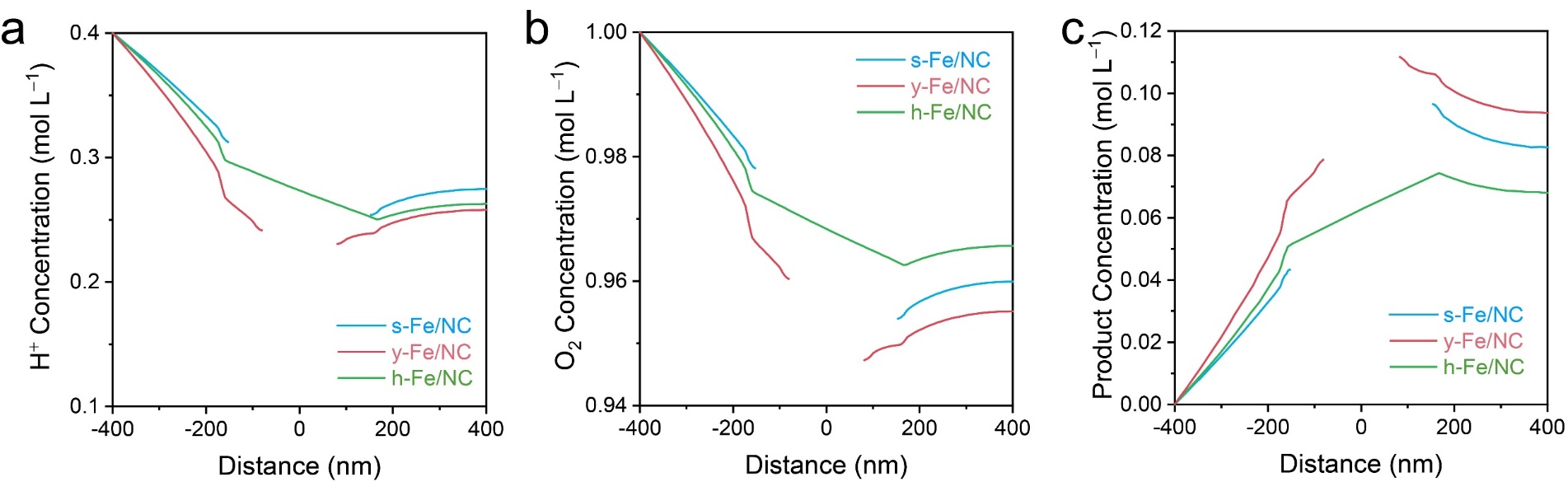


Fig. S37. Midline concentration distribution by FEM simulations.

Distribution of the (a) proton, (b) oxygen, and (c) obtained water concentration at the midline of the single model Fe/NC-based nanoreactors.


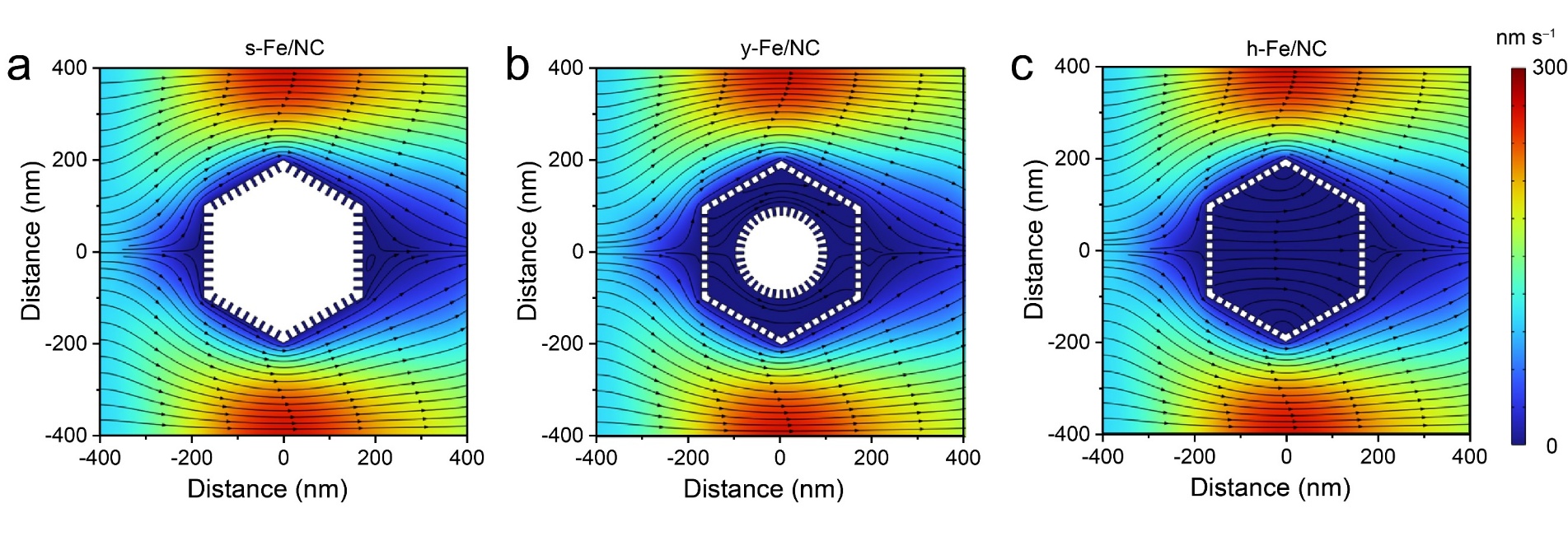


Fig. S38. O_2_ flow speed by FEM simulations.

Oxygen flow speed around the single model (a) s-Fe/NC, (b) y-Fe/NC, and (c) h-Fe/NC.


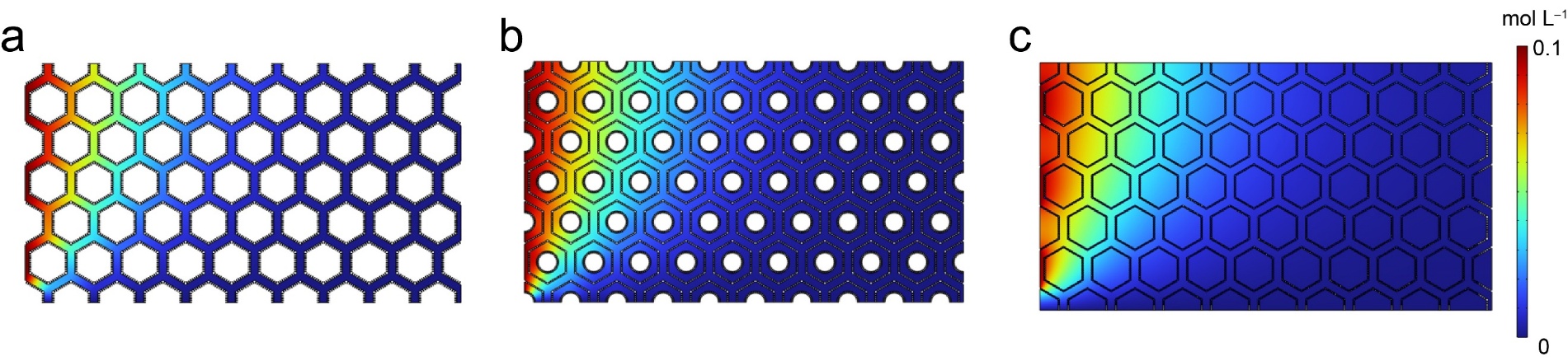


Fig. S39. Local H^+^ concentration.

Distribution of the proton concentration around the array model (a) s-Fe/NC, (b) y-Fe/NC, and (c) h-Fe/NC.


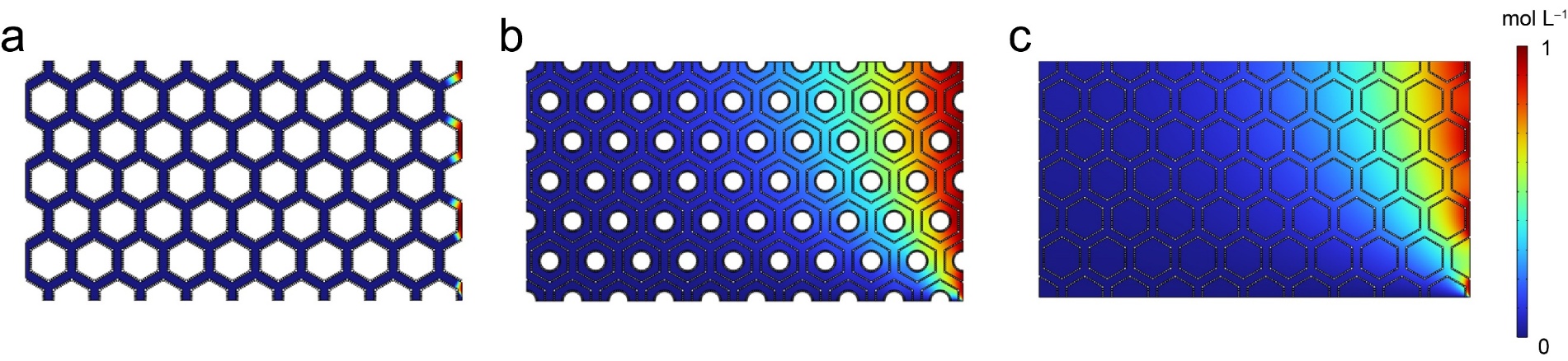


Fig. S40. Local O_2_ concentration.

Distribution of the oxygen concentration around the array model (a) s-Fe/NC, (b) y-Fe/NC, and (c) h-Fe/NC.


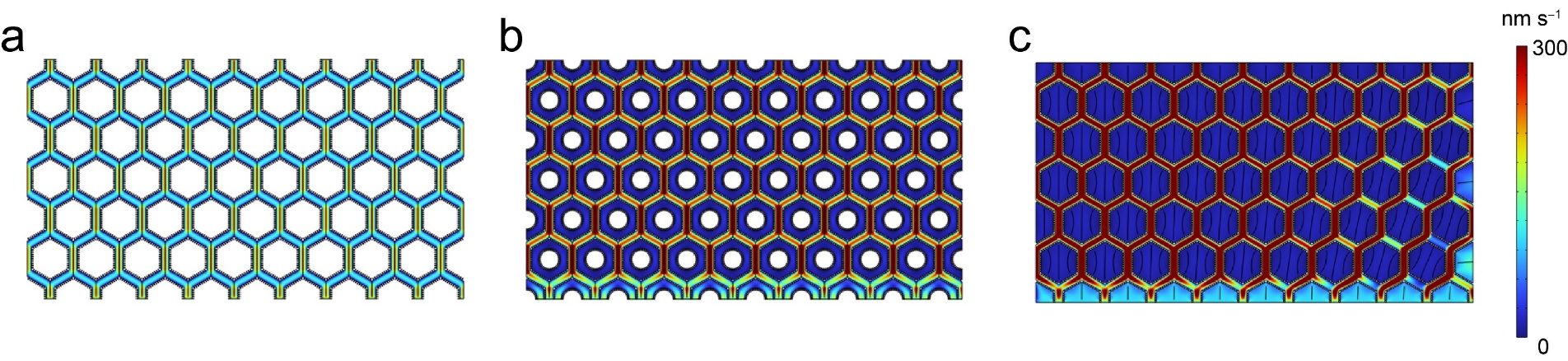


Fig. S41. O_2_ flow speed.

Oxygen flow speed around the array model (a) s-Fe/NC, (b) y-Fe/NC, and (c) h-Fe/NC.


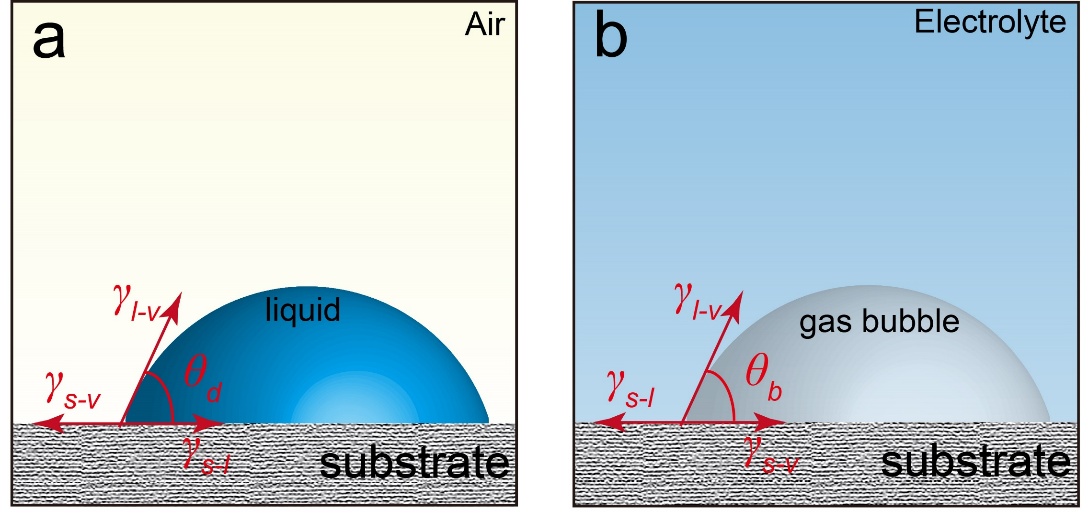


Fig. S42. Contact angle analysis.

Schematic illustration of the contact angle of (a) a droplet and (b) a bubble on a solid substrate. *θ_d_* is the droplet contact angle; *θ_b_* is the bubble contact angle; *γ_s-v_*, *γ_s-l_*, and *γ_l-v_* are the interfacial tensions for the solid-vapor, solid-liquid, and liquid-vapor interfaces, respectively.


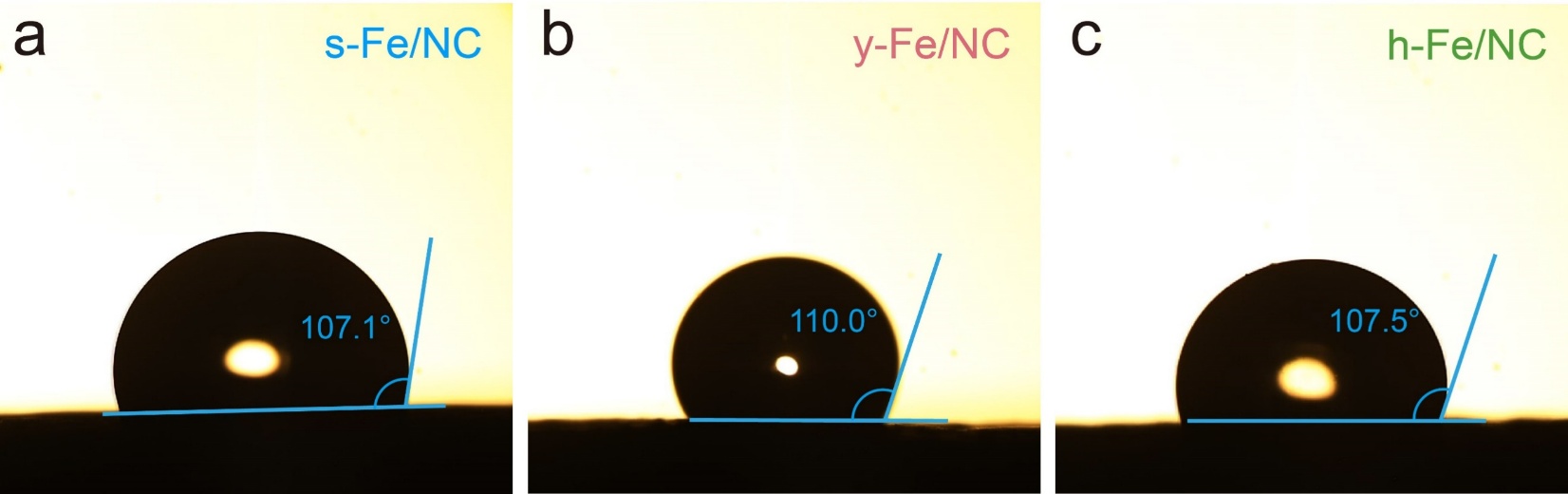


Fig. S43. Droplet contact angle.

The optical pictures and corresponding liquid contact angles for (a) s-Fe/NC, (b) y-Fe/NC, (c) h-Fe/NC using 0.1 M HClO_4_ as droplets.


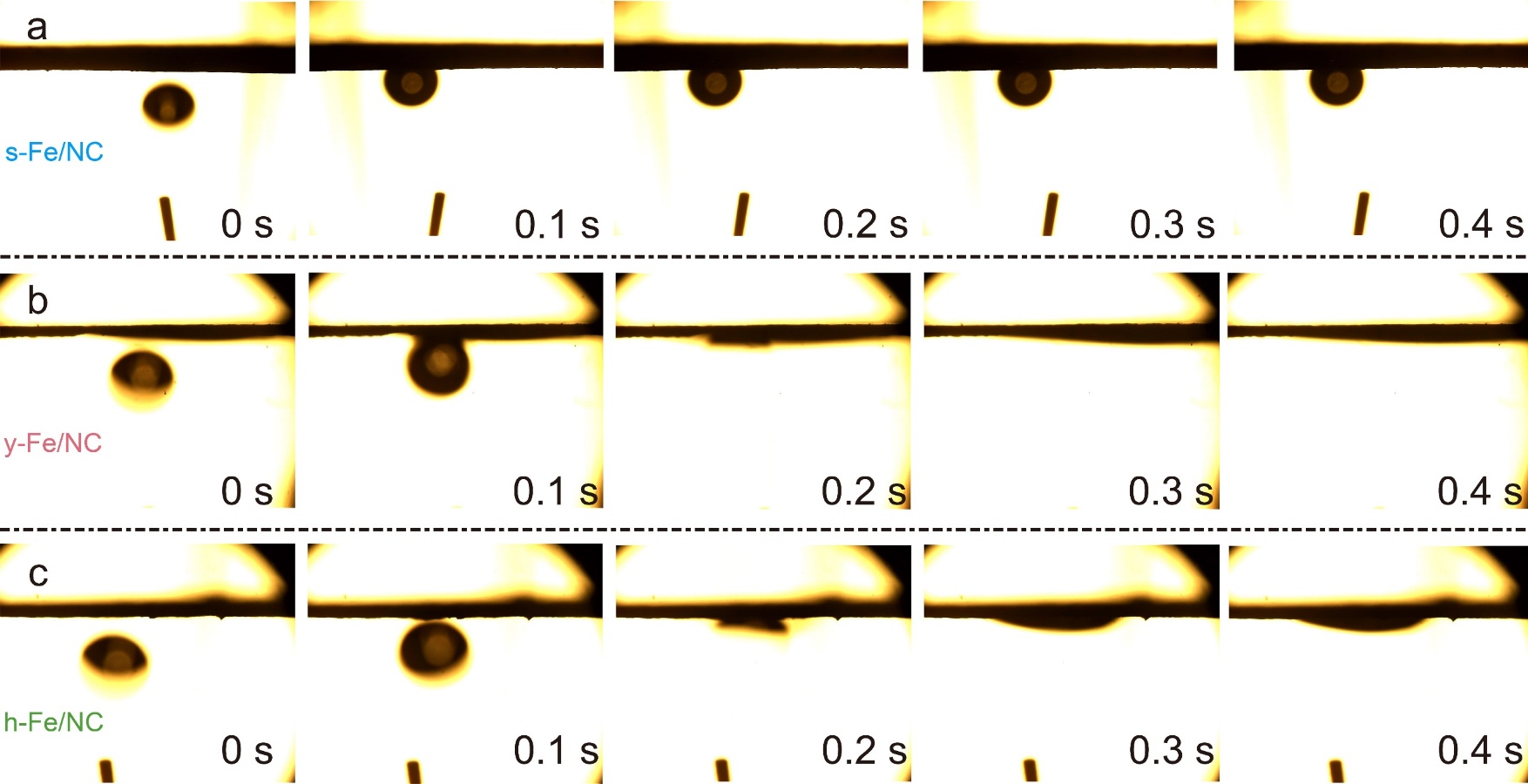


Fig. S44. Static gas bubble adhesion behaviors.

The optical pictures of static gas bubble behavior for (a) s-Fe/NC, (b) y-Fe/NC, (c) h-Fe/NC using 0.1 M HClO_4_ as solution.

Note S9. Aerophilicity effect on nanostructure design.

Theoretically, the basic principle for surface energy analysis in the determination of bubble wetting characteristics is the same as that for droplet^[32]^. As shown in Fig. S41, all Fe/NC samples exhibit hydrophobicity with similar contact angles of approximately 110°. However, the totally different gas bubble adhesion behaviors can be observed after nanostructure design. The gas bubble adhesion behaviors were measured under steady states, suggesting full contact between Fe/NC and the electrolyte. The adhesion behavior of gas bubbles under electrolyte on s-Fe/NC (Fig. S42a) is a typical pinning state, indicating that the gas pathway is blocked due to the isolated micropores and few mesopores^[33]^. However, for y-Fe/NC (Fig. S42b) and h-Fe/NC (Fig. S42c) with abundant mesopores within the carbon matrix and macropores that form the void, the gas bubbles show a significant bursting state, indicating a smooth air pathway^[34]^. Impressively, the gas capacity of y-Fe/NC is higher than that of h-Fe/NC, demonstrating the better resistance of the macropores against flooding. These bubble adhesion analysis are consistent well with the porosity analysis above^[32b]^.


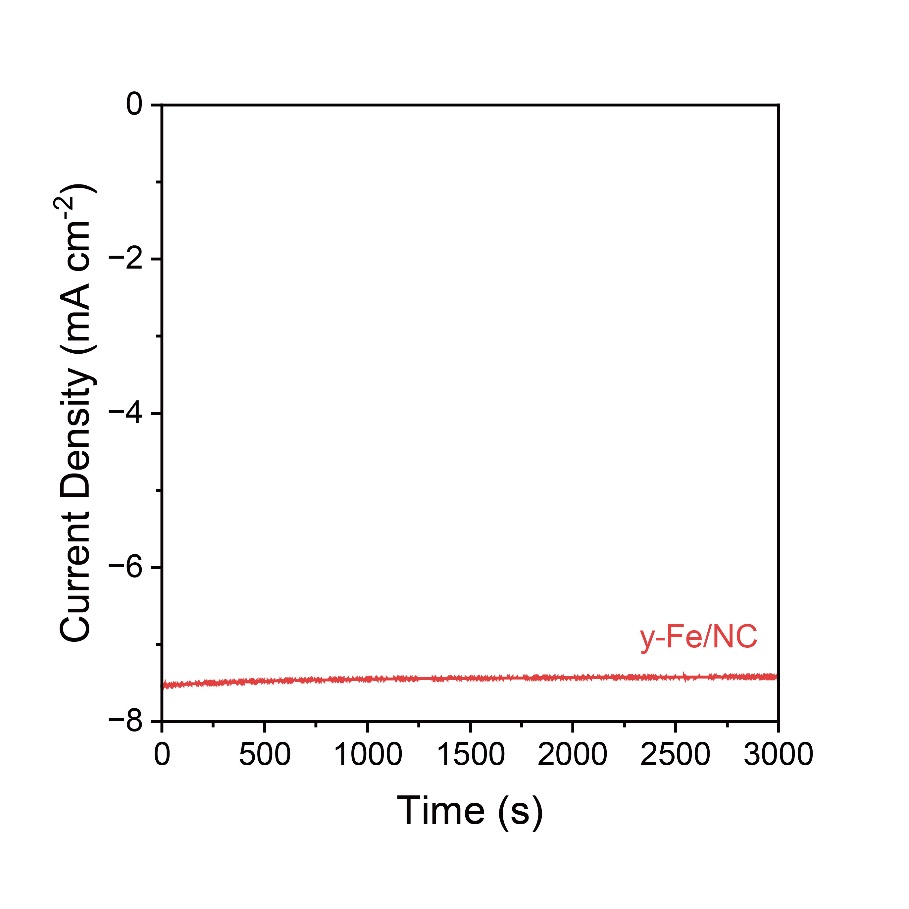


Fig. S45. Stability test for y-Fe/NC.

Amperometric i-t curve of y-Fe/NC.

Note S10. Theoretical model of BPCC system.

*In-situ* bubble pump consumption chronoamperometry (BPCC) strategy was performed in a home-made system^[35]^, as illustrated in Fig. S44. N_2_-saturated electrolyte was used in GDE evaluation to eliminate the dissolved oxygen influence, and a high overpotential was applied during chronoamperometry to ensure that the measured current is limited by mass transport rather than reaction kinetics. Thus, the *in situ* probe on GDE characteristics dominated by the O_2_ diffusion rate can be achieved by recording the responsive ORR current of each bubble. Based on different current behaviors, the ORR can be divided into two zones: extension zone (O_2_ spreads across the GDE to increase the reaction area and current) and consumption zone (current decreases until O_2_ is fully consumed). The extension-consumption (E-C) model can be described by the following equation:

$$\begin{aligned} i\left( t \right)=4\pi nFC_{0}hDkt\cdot\exp\left( -kt \right)=Akt\cdot\exp\left( -kt \right)\# SEQ EQ 32 \end{aligned}$$

Where *i* is the current, *t* is the extending time, *n* is the total number of electrons transferred during the electrochemical reaction, *F* is the Faraday constant, *C_0_* is the initial oxygen concentration, *h* is the inner thickness of GDE, *D* is the diffusion coefficient of O_2_ inside GDE, *k* is the effective reaction rate constant, and A is the pre-exponential factor (*4πnFC_0_hD*). Based on the established E-C model, the values of *A* and *k* could be obtained by fitting the current variations, representing OMT and SD, respectively.


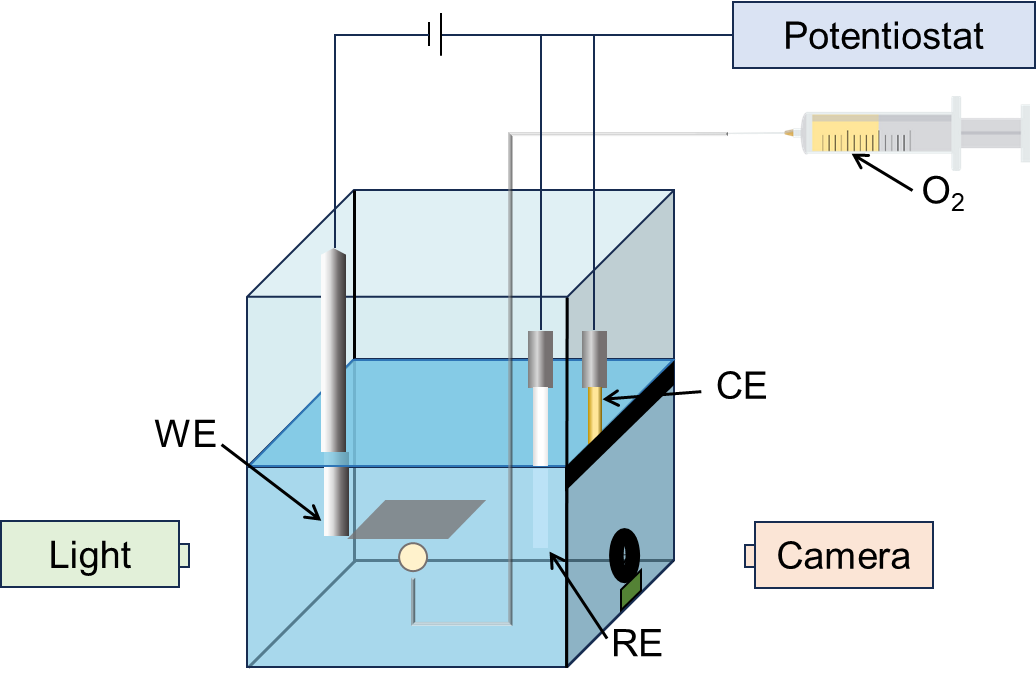


Fig. S46. Illustration of BPCC system.

Schematic illustration of home-made BPCC systems, where WE, RE, and CE represent working, reference, and counter electrode, respectively.


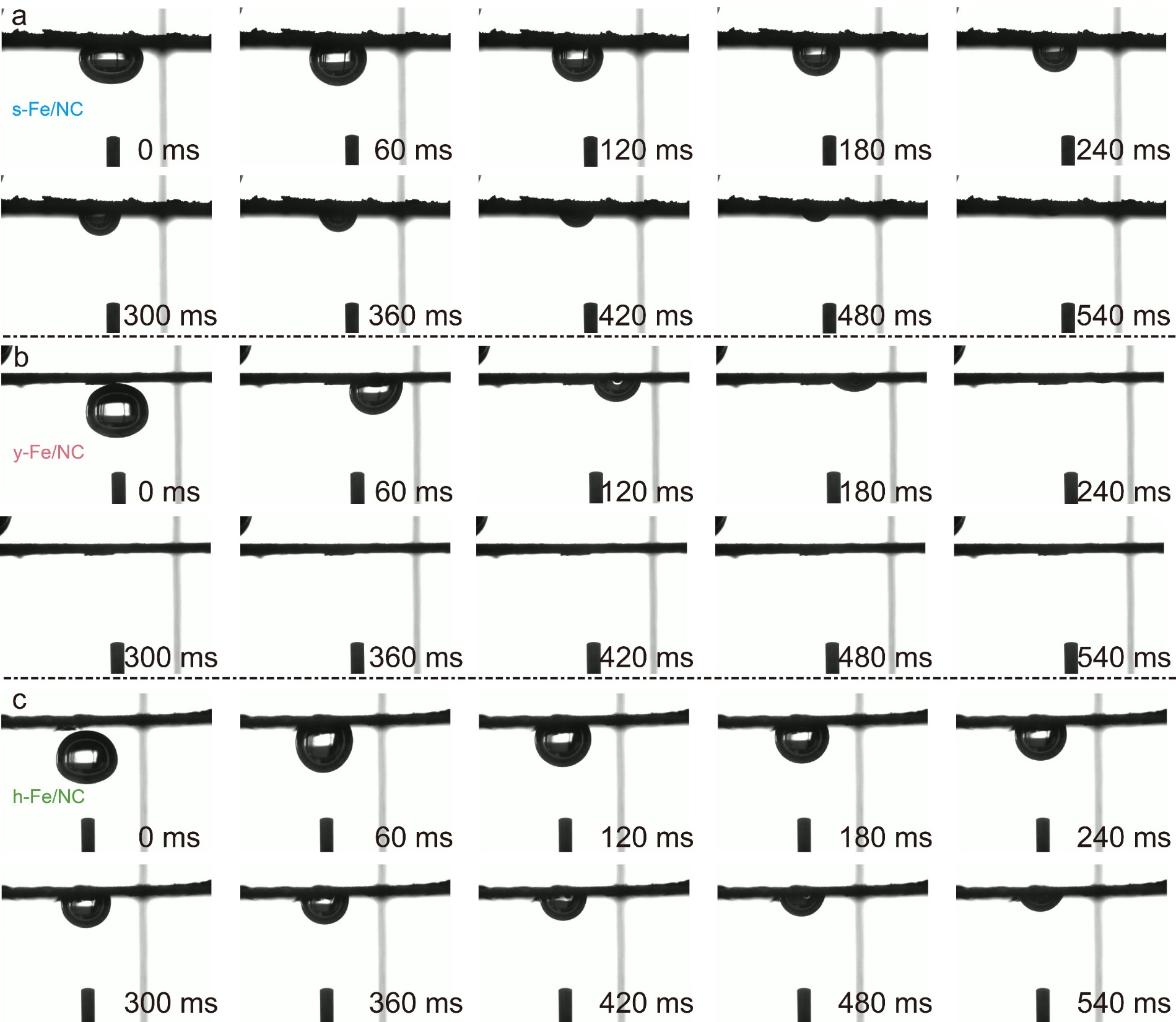


Fig. S47. Reacting O_2_ bubble behavior in BPCC.

The measurement of reacting O_2_ bubble behavior of (a) s-Fe/NC, (b) y-Fe/NC, and (c) h-Fe/NC.


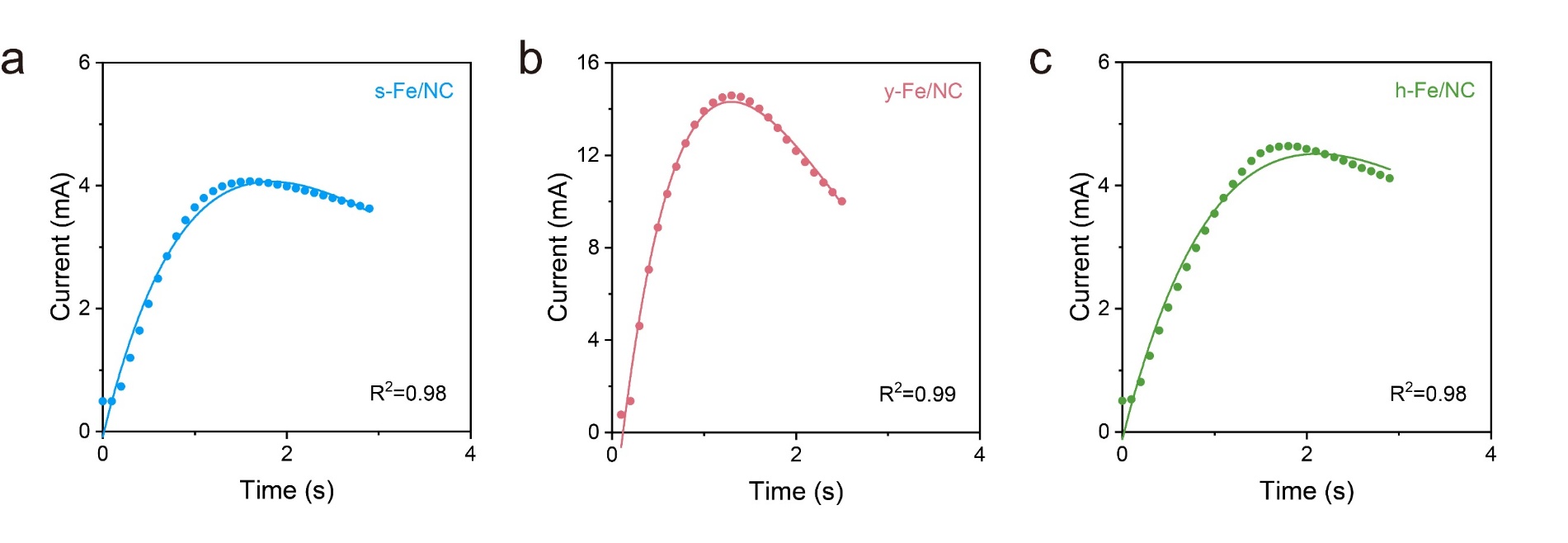


Fig. S48. Theoretical fitting in BPCC.

Fitting results for the ORR curve using the E-C model.


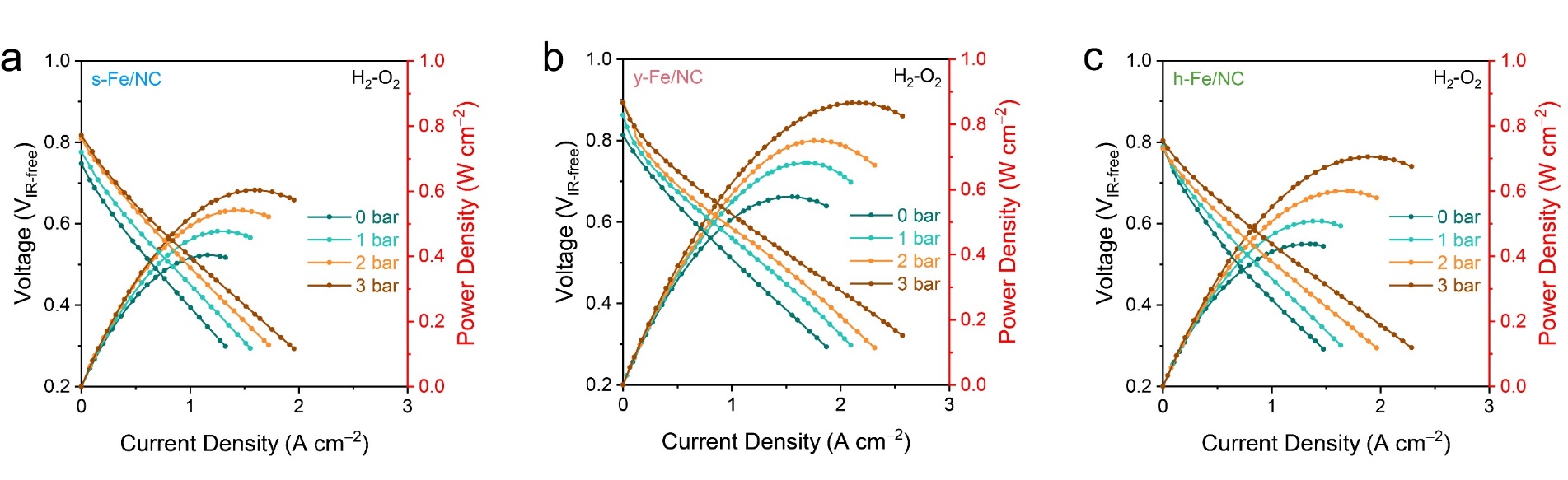


Fig. S49. Backpressure regulation.

Polarization and power density curves of (a) s-Fe/NC, (b) y-Fe/NC, and (c) h-Fe/NC under different backpressures. Catalyst loading: 3 mg cm^–2^ for Fe/NC nanoreactors.


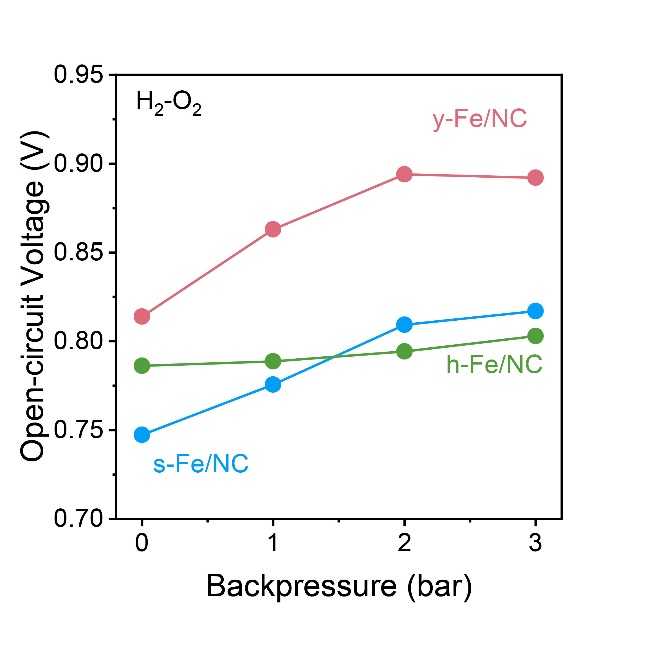


Fig. S50. Open-circuit voltage.

Relationship between open-circuit voltage and backpressure for all Fe/NC.


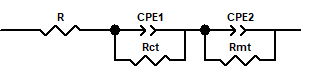


Fig. S51. Equivalent circuit model.

Equivalent circuit model for electrochemical impedance spectroscopy (EIS), including charge-transfer resistance (*R*_ct_), mass-transfer resistance (*R*_mt_), bulk resistance (R), and the constant phase element (CPE1 and CPE2) describing double-layer capacitive behavior. Zview software was used to fit the Nyquist plots.


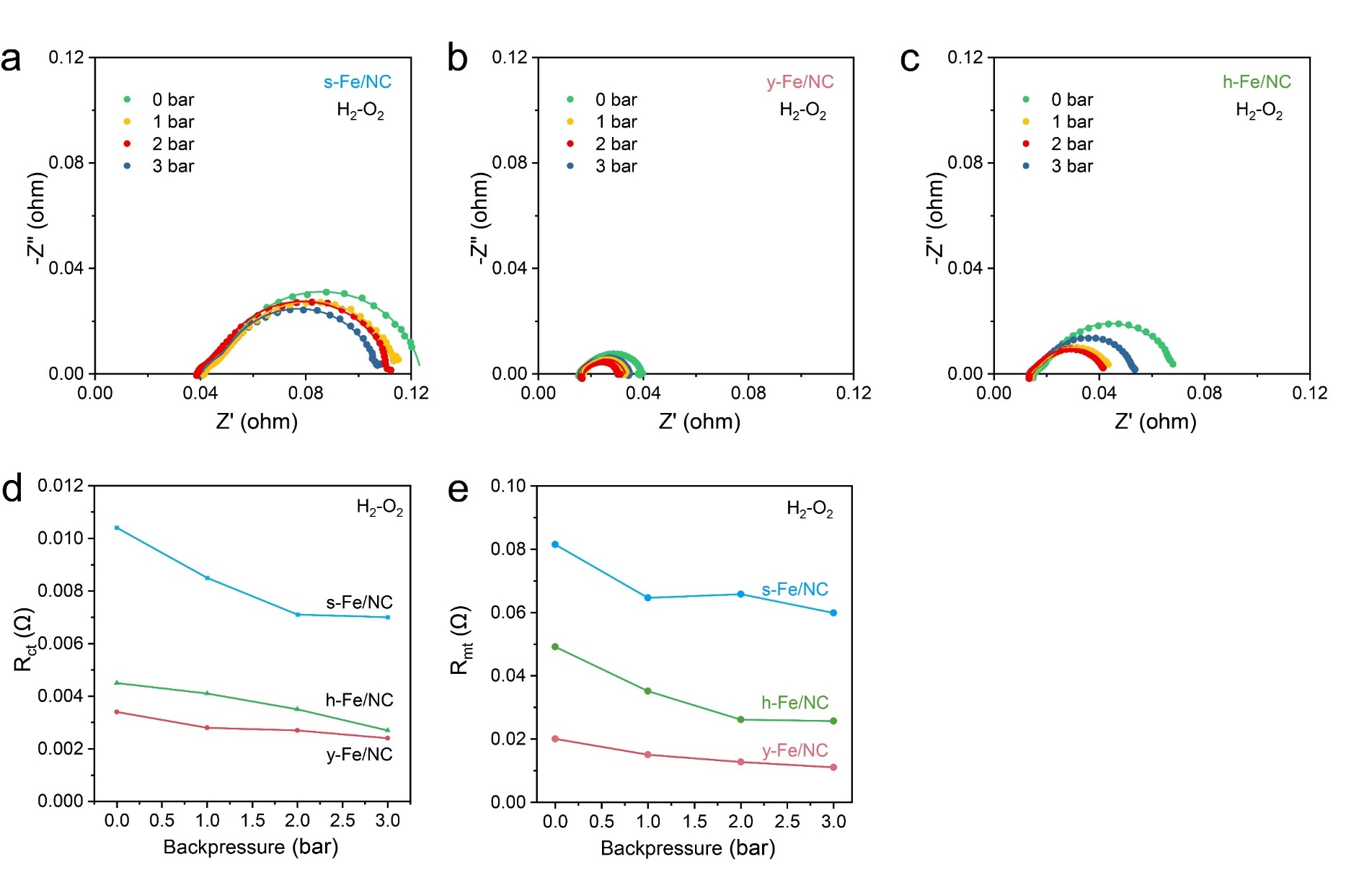


Fig. S52. Nyquist plot analysis.

Nyquist diagram for the fuel cell impedance measured of (a) s-Fe/NC, (b) y-Fe/NC, and (c) h-Fe/NC in H_2_-O_2_ fuel cell at different backpressures.


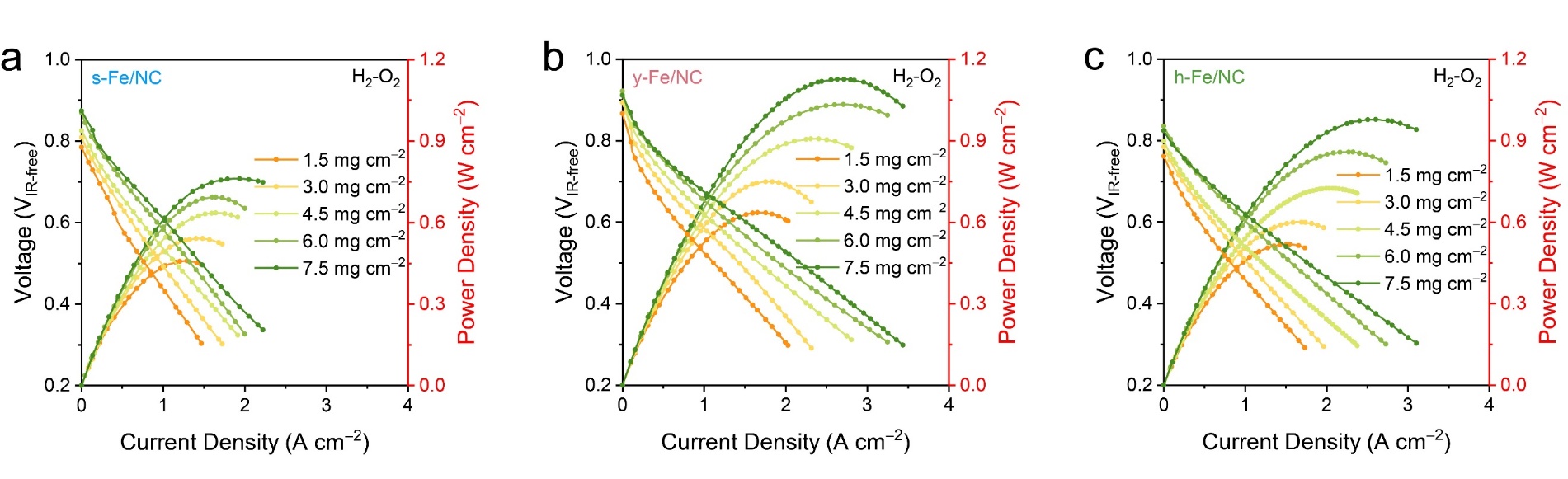


Fig. S53. Catalyst loading regulation.

Polarization and power density curves of (a) s-Fe/NC, (b) y-Fe/NC, and (c) h-Fe/NC under different loading amounts.


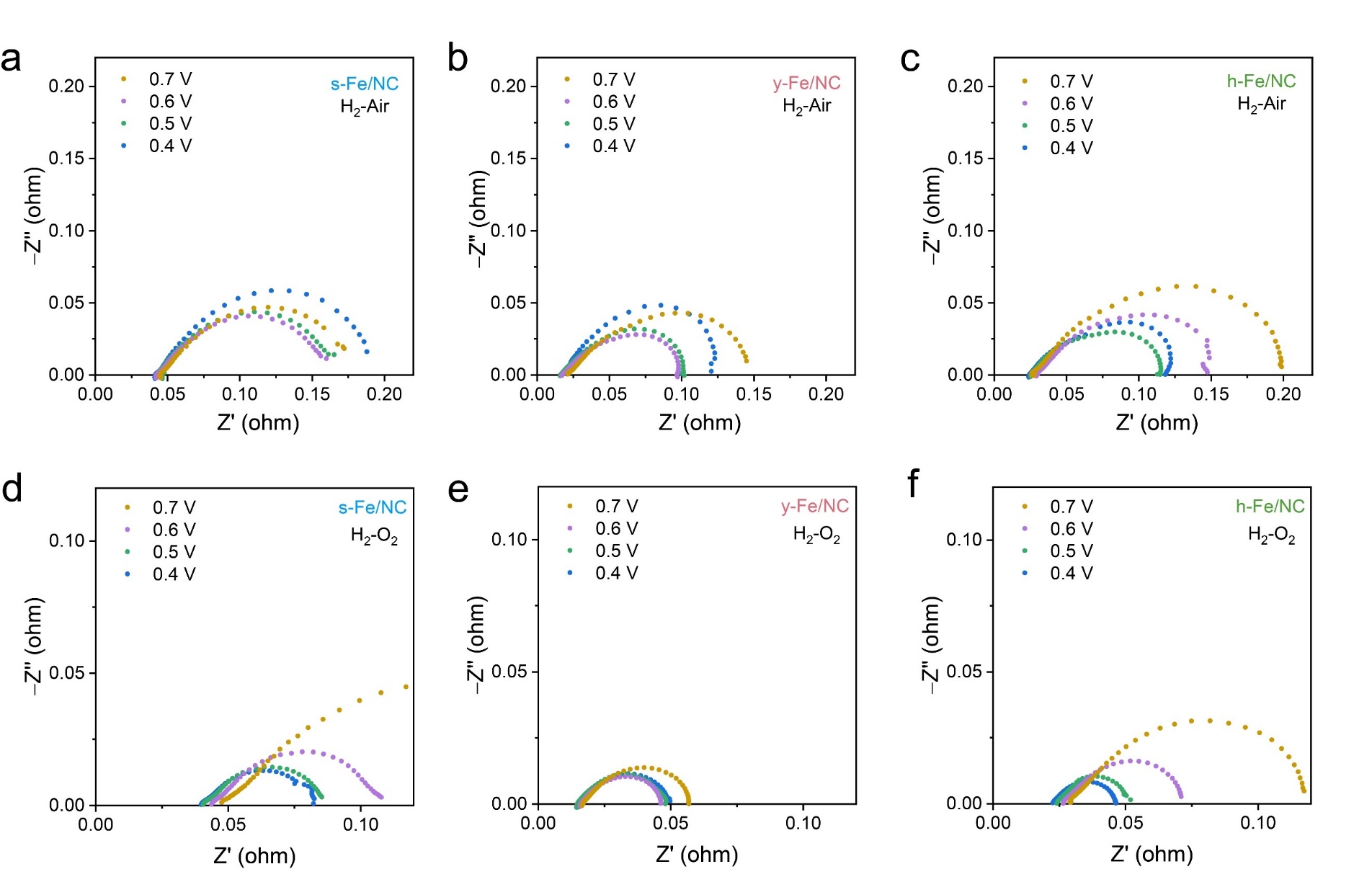


Fig. S54. Nyquist plot analysis.

Nyquist diagram for the fuel cell impedance measured of (a, d) s-Fe/NC, (b, e) y-Fe/NC, and (c, f) h-Fe/NC in (a-c) H_2_-Air and (d-f) H_2_-O_2_ fuel cells at different potentials.


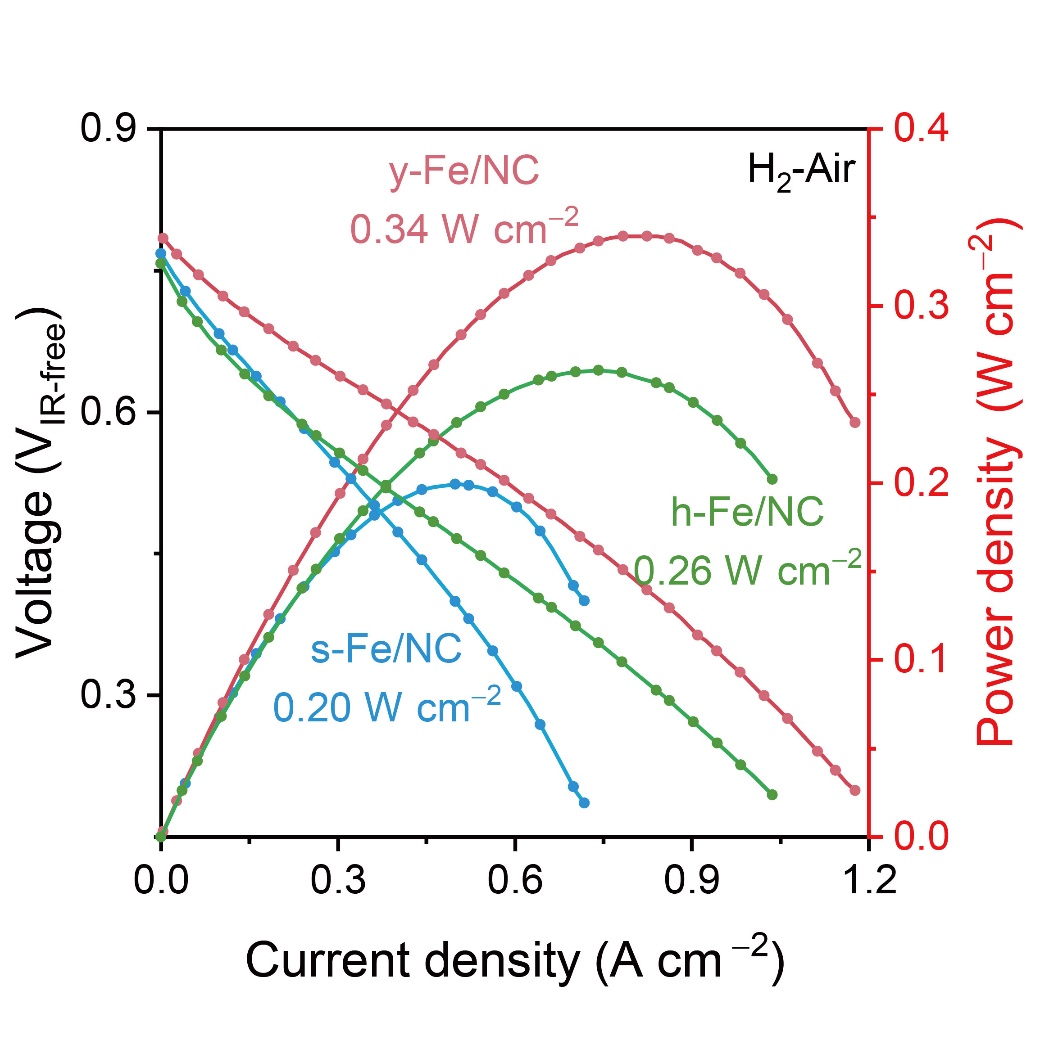


Fig. S55. PEMFC measurement.

Polarization and power density curves of Pt/C and Fe/NC nanoreactors under 2 bar H_2_ and air. Test conditions: catalysts loading 6 mg cm^–2^ for Fe/NC nanoreactors and 0.1 mgPt cm^−2^ for Pt/C.


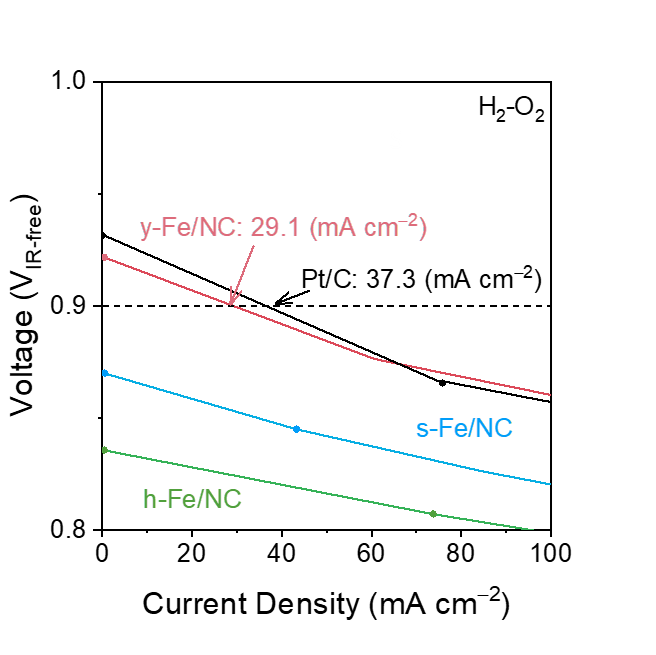


Fig. S56. Kinetic properties of PEMFC.

Zoomed in polarization curves of Pt/C and Fe/NC nanoreactors under 2 bar H_2_ and O_2_, where the red and black arrows indicate the current density of y-Fe/NC and Pt/C at 0.9 V_iR-free_.

Supplementary Tables:

Table S1. Metal loadings of Fe/NC determined by ICP-AES.

| sample | Fe (wt.%) |
| --- | --- |
| s-Fe/NC | 1.40 |
| y-Fe/NC | 1.36 |
| h-Fe/NC | 1.32 |
| y-Fe/NC after ADT | 1.33 |

Table S2. EXAFS fitting parameters at the Fe K-edge.

| Sample | Path | *C.N.* | *R* (Å) | *σ^2^*×10^3^(Å^2^) | *ΔE* (eV) | *R* factor |
| --- | --- | --- | --- | --- | --- | --- |
| s-Fe/NC | Fe-N | 4.03±1.4 | 2.03±0.03 | 6.53±8.8 | 6.65±5.1 | 0.020 |
| y-Fe/NC | Fe-N | 4.02±1.7 | 2.03±0.03 | 6.64±6.3 | 4.15±3.9 | 0.011 |
| h-Fe/NC | Fe-N | 4.02±1.8 | 2.03±0.03 | 7.47±6.9 | 4.67±3.9 | 0.012 |
| Fe foil | Fe-Fe_1_ | 8* | 2.46±0.03 | 4.89±2.7 | 7.54±4.8 | 0.004 |
|  | Fe-Fe_2_ | 6* | 2.85±0.03 | 3.79±4.6 |  |  |
| FePc | Fe-N | 3.98±0.8 | 1.91±0.03 | 3.96±2.5 | 4.33±2.6 | 0.021 |
|  | Fe-C | 8.12±4.9 | 3.17±0.03 | 2.56±3.2 |  |  |
| Fe_2_O_3_ | Fe-O | 6.57±1.0 | 2.01±0.03 | 5.62±2.2 | 2.54±1.4 | 0.027 |
|  | Fe-Fe | 6.40±1.7 | 3.07±0.03 | 6.30±1.9 |  |  |

*C.N.*: coordination number; *R*: distance between absorber and backscatter atoms; *σ^2^*: Debye-Waller factor to account for both thermal and structural disorders; *ΔE*: the inner potential correction. *R* factor (%): goodness of fit. *: The experimental EXAFS fit of the metal foil by fixing *C.N.* as the known crystallographic value. *S_0_^2^* was fixed to 0.8 as determined from Fe foil fitting. Fitting range: 3.0≤*k* (Å^−1^)≤10 and 1≤*R* (Å)≤2.5.

Table S3. Elemental contents of Fe/NC determined by XPS.

| sample | Fe (at.%) | N (at.%) | C (at.%) |
| --- | --- | --- | --- |
| s-Fe/NC | 0.78 | 8.78 | 90.44 |
| y-Fe/NC | 0.62 | 8.80 | 90.59 |
| h-Fe/NC | 0.76 | 8.66 | 90.58 |

Table S4. Performance comparison for ORR catalysts in acid.

| Catalysts | 900 rpm | | 1600 rpm | | Ref. |
| --- | --- | --- | --- | --- | --- |
|  | E_1/2_ (V) | *j_d_* (mA cm^−2^) | E_1/2_ (V) | *j_d_* (mA cm^−2^) |  |
| s-Fe/NC | 0.79 | 3.81 | 0.78 | 5.16 | this work |
| y-Fe/NC | 0.81 | 4.95 | 0.82 | 7.66 | this work |
| h-Fe/NC | 0.78 | 4.08 | 0.80 | 5.67 | this work |
| Pt/C | - | - | 0.86 | 5.05 | this work |
| Pt-PIL@HCS |  |  | 0.85 | 10.3 | *J. Am. Chem. Soc*. **2025**, 147, 3421 |
| F-Fe-N-C | - | - | 0.83 | ~6.2 | *ACS Energy Lett.* **2025**, 10, 2743 |
| FeSA−N/TC | - | - | 0.82 | ~6.0 | *Angew. Chem. Int. Ed.* **2024**, 64, e202415691 |
| FeN_x_Se_y_ | - | - | 0.89 | ~6.0 | *Angew. Chem. Int. Ed.* **2024**, 64, e202419501 |
| FeAC-N-SC | - | - | 0.82 | ~5.0 | *J. Am. Chem. Soc.* **2025** |
| Fe–S–NC/Fe_3_C | - | - | 0.80 | ~6.0 | *Energy Environ. Sci.* **2024**, 17, 5941 |
| TPI@Z8(SiO_2_)-  650-C | - | - | 0.82 | ~5.5 | *Nat. Catal.* **2019**, 2, 259 |
| Fe-N_4_-0.5 | ~0.82 | ~5.1 | ~0.81 | ~6.6 | *Nat. Catal.* **2021**, 4, 615 |
| FeNC-CVD-750 | 0.85 | ~3.8 | - | - | *Nat. Mater.* **2021**, 20, 1385 |
| Fe-NC^Δ-DCDA^ | 0.82 | ~3.8 | - | - | *Nat. Catal.* **2022**, 5, 311 |
| (CM+PANI)Fe-C | 0.80 | ~3.0 | - | - | *Science* **2017,** 357, 479 |
| FeCl_2_-NC-1000 | - | - | 0.80 | ~5.5 | *J. Am. Chem. Soc.* **2020,** 142, 1417 |

Table S5. BET surface area and the pore volume of Fe/NCs.

| Sample | BET surface area (m^2^ g^−1^) | Volume of micropores (cm^3^ g^−1^) | Volume of mesopores (cm^3^ g^−1^) |
| --- | --- | --- | --- |
| s-Fe/NC | 892.7 | 0.28 | 0.25 |
| y-Fe/NC | 677.5 | 0.13 | 0.94 |
| h-Fe/NC | 528.2 | 0.10 | 0.83 |

Table S6. Summary of the surface area of all Fe/NCs.

| Sample | GEO area (cm^2^) | BET surface area (m^2^ g^−1^) | ECSA (cm^2^_ECSA_) |
| --- | --- | --- | --- |
| s-Fe/NC | 0.0707 | 892.7 | 405 |
| y-Fe/NC | 0.0707 | 677.5 | 1190 |
| h-Fe/NC | 0.0707 | 528.2 | 758 |

Table S7. CO adsorption capacity.

| Peak number | Cumulative quantity (cm^3^ g^−1^ STP) | | |
| --- | --- | --- | --- |
|  | s-Fe/NC | y-Fe/NC | h-Fe/NC |
| 1 | 1.35 | 1.63 | 1.30 |
| 2 | 1.47 | 1.76 | 1.37 |
| 3 | 1.49 | 1.82 | 1.41 |
| 4 | 1.50 | 1.86 | 1.43 |
| 5 | 1.51 | 1.86 | 1.55 |
| 6 | 1.51 | 1.86 | 1.58 |

Table S8. Summary of MSD of all Fe/NCs.

| Sample | ICP (sites g^−1^) | CO (sites g^−1^) | NS (sites g^−1^) |
| --- | --- | --- | --- |
| s-Fe/NC | 1.51×10^20^ | 4.06×10^19^ | 8.75×10^18^ |
| y-Fe/NC | 1.47×10^20^ | 5.00×10^19^ | 3.39×10^19^ |
| h-Fe/NC | 1.42×10^20^ | 4.26×10^19^ | 1.98×10^19^ |

Table S9. EIS fitting results.

| Sample | pressure | R (Ω) | CPE-T | CPE-P | R_ct_ (Ω) | CPE-T | CPE-P | R_mt_ (Ω) |
| --- | --- | --- | --- | --- | --- | --- | --- | --- |
| s-Fe/NC | 0 bar | 0.041 | 0.023 | 0.985 | 0.010 | 0.276 | 0.809 | 0.082 |
|  | 1 bar | 0.042 | 0.181 | 0.777 | 0.009 | 0.232 | 0.886 | 0.065 |
|  | 2 bar | 0.039 | 0.147 | 0.829 | 0.007 | 0.287 | 0.880 | 0.066 |
|  | 3 bar | 0.041 | 0.063 | 0.899 | 0.007 | 0.231 | 0.874 | 0.060 |
| y-Fe/NC | 0 bar | 0.016 | 0.269 | 0.848 | 0.003 | 0.595 | 0.814 | 0.020 |
|  | 1 bar | 0.017 | 0.111 | 1.037 | 0.003 | 0.526 | 0.854 | 0.015 |
|  | 2 bar | 0.017 | 0.174 | 0.986 | 0.003 | 0.681 | 0.861 | 0.013 |
|  | 3 bar | 0.017 | 0.124 | 1.036 | 0.002 | 0.707 | 0.850 | 0.011 |
| h-Fe/NC | 0 bar | 0.016 | 0.621 | 0.763 | 0.004 | 1.209 | 0.837 | 0.049 |
|  | 1 bar | 0.014 | 0.857 | 0.772 | 0.004 | 1.314 | 0.842 | 0.035 |
|  | 2 bar | 0.014 | 0.643 | 0.819 | 0.004 | 1.397 | 0.832 | 0.026 |
|  | 3 bar | 0.014 | 0.706 | 0.834 | 0.003 | 1.677 | 0.808 | 0.026 |

Table S10. PEMFC performance comparison.

| Catalyst | Loading (mg cm^−2^) | Backpressure (bar) | Peak power density (W cm^−2^) | Reference |
| --- | --- | --- | --- | --- |
| y-Fe/NC | 6 | 2 | 1.03 | This work |
| s-Fe/NC | 6 | 2 | 0.69 | This work |
| h-Fe/NC | 6 | 2 | 0.86 | This work |
| OP-Fe-NC | 4 | 2 | 0.94 | *J. Am. Chem. Soc.* **2023**, 145, 27262 |
| F-Fe-N-C | 3 | 1 | 0.9 | *ACS Energy Lett.* **2025**, 10, 2743 |
| FeSA/FeAC-2DNPC | 1.5 | 2 | 0.94 | *Nat. Commun.* **2022**, 13, 2963 |
| Co(mIm)-NC(1.0) | 6.3 | 3 | 0.64 | *Nat. Catal.* **2020**, 3, 1044 |
| TPI@Z8(SiO_2_)-650-C | 2.7 | 2.5 | 1.18 | *Nat. Catal.* **2019**, 2, 259 |
| (CM+PANI)-Fe-C | 4 | 2 | 0.94 | *Science* **2017**, 357, 479 |
| Fe/N/CF | 2 | 2 | 0.90 | *Proc. Natl. Acad. Sci.* **2015**, 112, 10629 |
| sur-FeN_4_-HPC | 4 | 2 | 0.79 | *Energy Environ. Sci.* **2022**, 15, 2619 |
| Fe–S–NC/Fe_3_C | 3 | 2 | 0.84 | *Energy Environ. Sci.* **2024**, 17, 5941 |
| 1.5Fe-ZIF | 4 | 1 | 0.67 | *Energy Environ. Sci.* **2019**, 12, 2548 |
| FeSA−N/TC | 4 | 1.5 | 1.10 | *Angew. Chem. Int. Ed.* **2024**, 64, e202415691 |
| ZIF-NC-0.5Fe-700 | 3.5 | 1 | 0.73 | *Angew. Chem. Int. Ed.* **2019**, 58, 18971 |
| 0.17CVD/Fe-NC-*kat* | 4 | 1 | 0.70 | *Angew. Chem. Int. Ed.* **2020**, 59, 21698 |
| FeN_x_Se_y_ | 3 | 1 | 0.72 | *Angew. Chem. Int. Ed.* **2024**, 64, e202419501 |
| FeAC-N-SC | 4 | 1.5 | 1.10 | *J. Am. Chem. Soc.* **2025** |

References

1. S. Brunauer, P. H. Emmett, E. Teller, *J. Am. Chem. Soc.* **1938**, 60, 309.

2. E. P. Barrett, L. G. Joyner, P. P. Halenda, *J. Am. Chem. Soc.* **1951**, 73, 373.

3. B. Ravel, M. Newville, *J. Synchrotron Radiat.* **2005**, 12, 537.

4. H. Fei, J. Dong, Y. Feng, C. S. Allen, C. Wan, B. Volosskiy, M. Li, Z. Zhao, Y. Wang, H. Sun, P. An, W. Chen, Z. Guo, C. Lee, D. Chen, I. Shakir, M. Liu, T. Hu, Y. Li, A. I. Kirkland, X. Duan, Y. Huang, *Nat. Catal.* **2018**, 1, 63.

5. A. L. Ankudinov, B. Ravel, J. J. Rehr, S. D. Conradson, *Phys. Rev. B* **1998**, 58, 7565.

6. a) H. Funke, A. C. Scheinost, M. Chukalina, *Phys. Rev. B* **2005**, 71, 094110; b) H. Funke, M. Chukalina, A. C. Scheinost, *J. Synchrotron Radiat.* **2007**, 14, 426.

7. a) Y. Joly, *Phys. Rev. B* **2001**, 63, 125120; b) O. Bunău, Y. Joly, *J. Phys. Condens. Matter* **2009**, 21, 345501; c) J. J. Rehr, R. C. Albers, *Rev. Mod. Phys.* **2000**, 72, 621.

8. W. Xu, Z. Liang, S. Gong, B. Zhang, H. Wang, L. Su, X. Chen, N. Han, Z. Tian, T. Kallio, L. Chen, Z. Lu, X. Sun, *ACS Sustain. Chem. Eng.* **2021**, 9, 7120.

9. T. Zhang, F. Wang, C. Yang, X. Han, C. Liang, Z. Zhang, Y. Li, A. Han, J. Liu, B. Liu, *Chem Catal.* **2022**, 2, 836.

10. L. Jiao, J. Li, L. L. Richard, Q. Sun, T. Stracensky, E. Liu, M. T. Sougrati, Z. Zhao, F. Yang, S. Zhong, H. Xu, S. Mukerjee, Y. Huang, D. A. Cullen, J. H. Park, M. Ferrandon, D. J. Myers, F. Jaouen, Q. Jia, *Nat. Mater.* **2021**, 20, 1385.

11. L. Osmieri, A. H. A. Monteverde Videla, P. Ocón, S. Specchia, *J. Phys. Chem. C* **2017**, 121, 17796.

12. N. Wakabayashi, M. Takeichi, M. Itagaki, H. Uchida, M. Watanabe, *J. Electroanal. Chem.* **2005**, 574, 339.

13. X. Han, T. Zhang, X. Wang, Z. Zhang, Y. Li, Y. Qin, B. Wang, A. Han, J. Liu, *Nat. Commun.* **2022**, 13, 2900.

14. V. D. M. Bonazzoli, I. G. Graham, E. A. Spence, P.-H. Tournier, *Math. Comp.* **2019**, 88, 2559.

15. G. Kresse, D. Joubert, *Phys. Rev. B* **1999**, 59, 1758.

16. X. Ge, C. Li, Z. Li, L. Yin, *Electrochim. Acta* **2018**, 281, 700.

17. W. Chen, Q. Xiang, T. Peng, C. Song, W. Shang, T. Deng, J. Wu, *iScience* **2020**, 23, 101532.

18. E. Higuchi, H. Uchida, M. Watanabe, *J. Electroanal. Chem.* **2005**, 583, 69.

19. Y. Garsany, I. L. Singer, K. E. Swider-Lyons, *J. Electroanal. Chem.* **2011**, 662, 396.

20. K. J. J. Mayrhofer, D. Strmcnik, B. B. Blizanac, V. Stamenkovic, M. Arenz, N. M. Markovic, *Electrochim. Acta* **2008**, 53, 3181.

21. S. Treimer, A. Tang, D. C. Johnson, *Electroanalysis* **2002**, 14, 165.

22. G. Zhong, S. Xu, L. Liu, C. Z. Zheng, J. Dou, F. Wang, X. Fu, W. Liao, H. Wang, *ChemElectroChem* **2020**, 7, 1107.

23. a) J. O. M. Bockris, Z. Nagy, *J. Chem. Educ.* **1973**, 50, 839; b) J. Wang, C.-X. Zhao, J.-N. Liu, D. Ren, B.-Q. Li, J.-Q. Huang, Q. Zhang, *Nano Mater. Sci.* **2021**, 3, 313; c) J. Chen, M. Aliasgar, F. B. Zamudio, T. Zhang, Y. Zhao, X. Lian, L. Wen, H. Yang, W. Sun, S. M. Kozlov, W. Chen, L. Wang, *Nat. Commun.* **2023**, 14, 1711; d) S. Xu, Y. Kim, D. Higgins, M. Yusuf, T. F. Jaramillo, F. B. Prinz, *Electrochim. Acta* **2017**, 255, 99.

24. M. H. Seo, S. M. Choi, H. J. Kim, W. B. Kim, *Electrochem. Commun.* **2011**, 13, 182.

25. B. Nam, R. T. Bonnecaze, *J. Electrochem. Soc.* **2007**, 154, F191.

26. J. Visuvasam, A. Molina, E. Laborda, L. Rajendran, *Int. J. Electrochem. Sci.* **2018**, 13, 9999.

27. R. T. Bonnecaze, N. Mano, B. Nam, A. Heller, *J. Electrochem. Soc.* **2007**, 154, F44.

28. C. Weidenthaler, *Nanoscale* **2011**, 3, 792.

29. T. A. Greszler, D. Caulk, P. Sinha, *J. Electrochem. Soc.* **2012**, 159, F831.

30. F. Yin, Y. Liu, C. Wang, H. Liu, *Phys. Chem. Chem. Phys.* **2018**, 20, 16159.

31. W. Henry, J. Banks, *Philos. Trans. Royal Soc.* **1997**, 93, 29.

32. a) T. S. Chow, *J. Phys. Condens. Matter* **1998**, 10, L445; b) W. Xu, Z. Lu, X. Sun, L. Jiang, X. Duan, *Acc. Chem. Res.* **2018**, 51, 1590.

33. J. Wang, Q. Yang, M. Wang, C. Wang, L. Jiang, *Soft Matter* **2012**, 8, 2261.

34. J. Wang, Y. Zheng, F.-Q. Nie, J. Zhai, L. Jiang, *Langmuir* **2009**, 25, 14129.

35. M. Li, W. Xu, D. Zhou, Y. Zhang, Y. Kuang, H. Liu, X. Wang, Y. Zhong, Z. Zhuang, H. Li, L. Luo, X. Sun, *Chem Catal.* **2023**, 3, 100769.
